# DNA methylation signatures to predict the cervicovaginal microbiome status

**DOI:** 10.1186/s13148-020-00966-7

**Published:** 2020-11-23

**Authors:** Nuno R. Nené, James Barrett, Allison Jones, Iona Evans, Daniel Reisel, John F. Timms, Tobias Paprotka, Andreas Leimbach, Dorella Franchi, Nicoletta Colombo, Line Bjørge, Michal Zikan, David Cibula, Martin Widschwendter

**Affiliations:** 1 Department of Women’s Cancer, EGA Institute for Women’s Health, University College London, London, UK; 2 Department of Mathematics, University College London, London, UK; 3 European Translational Oncology Prevention and Screening (EUTOPS) Institute, 6060 Hall in Tirol, Austria; 4 Research Institute for Biomedical Aging Research, Universität Innsbruck, 6020 Innsbruck, Austria; 5 Eurofins Genomics Europe Sequencing, Constance, Germany; 6 Europeo Di Oncologia, IRCCS, Milan, Italy; 7 University of Milano-Bicocca, Milan, Italy; 8 Department of Obstetrics and Gynecology, Haukeland University Hospital, Bergen, Norway; 9 Centre for Cancer Biomarkers, Department of Clinical Science, CCBIO, University of Bergen, Bergen, Norway; 10 Hospital Na Bulovce, Prague, Czech Republic; 11 Department of Obstetrics and Gynecology, General University Hospital in Prague, First Faculty of Medicine, Charles University, Prague, Czech Republic

**Keywords:** Cervicovaginal microbiome, DNA methylation, Epigenome–microbiome interaction, Penalized regression

## Abstract

**Background:**

The composition of the microbiome plays an important role in human health and disease. Whether there is a direct association between the cervicovaginal microbiome and the host’s epigenome is largely unexplored.

**Results:**

Here we analyzed a total of 448 cervicovaginal smear samples and studied both the DNA methylome of the host and the microbiome using the Illumina EPIC array and next-generation sequencing, respectively. We found that those CpGs that are hypo-methylated in samples with non-lactobacilli (O-type) dominating communities are strongly associated with gastrointestinal differentiation and that a signature consisting of 819 CpGs was able to discriminate lactobacilli-dominating (L-type) from O-type samples with an area under the receiver operator characteristic curve (AUC) of 0.84 (95% CI = 0.77–0.90) in an independent validation set. The performance found in samples with more than 50% epithelial cells was further improved (AUC 0.87) and in women younger than 50 years of age was even higher (AUC 0.91). In a subset of 96 women, the buccal but not the blood cell DNA showed the same trend as the cervicovaginal samples in discriminating women with L- from O-type cervicovaginal communities.

**Conclusions:**

These findings strongly support the view that the epithelial epigenome plays an essential role in hosting specific microbial communities.

## Background

The microbiome plays an essential role in human health and disease, with its composition being one of the most important factors. An ‘imbalanced’ microbiome, such as that in bacterial vaginosis or clostridium difficile infection, can be treated with variable success by directly interfering with its composition (i.e., by applying antibiotics and transplanting the microbiome from healthy individuals) [1, 2]. There is conclusive evidence, particularly in microbiome studies in the gut, that it is not the host’s genetic ancestry, but rather environmental factors such as diet and drugs, that shape the microbiome and account for most of the inter-individual variability [3]. Whether environmental factors impact directly on the microbiome or indirectly via alterations of the host’s cells is unknown. However, factors that are known to shape the microbiome such as age [4], body mass index [3], smoking [5] and nonsteroidal anti-inflammatory drugs [6] are also known to impact on the host’s epigenome [7–10] and therefore its cell identity and function [11]. The epigenetic landscape of the host cells and its contribution to the composition of the microbiome has not yet been studied in any depth. Here, we assessed whether the host’s DNA methylome is associated with the cervicovaginal microbiome and whether any such relationship depends on the host cell type and age.

The physiological cervicovaginal microbiome is dominated by four types of Lactobacilli: *L crispatus, L gasseri, L iners and L jensenii* [12, 13]. These Lactobacilli are associated with a substantially lower vaginal pH [12], potentially decreasing the risk of ascending infections. In a previous study, we classified samples according to their proportion of Lactobacilli; samples where at least 50% of the cervicovaginal microbiota belonged to the group of Lactobacilli highlighted above were labeled as having L community-type and samples with less than 50% as O community-type (Other) [4]. The presence of ovarian cancer or factors that have been proven to affect the risk of this cancer, such as *BRCA1* germline mutations, were significantly associated with the community-type O cervicovaginal microbiota [4].

## Results

Here we analyzed the DNA methylation of 448 cervicovaginal smear samples for which we had microbiome data available [4] (see Additional file 1: Table S1 for the association between covariates and community-type). We split samples into a training (n = 311, 161 L-type, 150 O-type) and a validation set (n = 137, 71 L-type, 66 O-type) prior to analysis. These were stratified for age, immune cell proportion, and community-type (Additional file 1: Fig. S1, see also Additional file 1: Fig. S2 for overall species abundance). Previously, we found that methylation differences vary due to immune cell-type composition in cases compared to controls [14, 15], and it is therefore important to assess the level of cell-type heterogeneity in each cervical smear sample as a first step in the analysis pathway. This was accomplished by applying EpiDISH [16], an algorithm that infers the relative proportion of epithelial cells, fibroblasts, and seven subtypes of immune cells (ICs) in each sample. The estimated cell-type distributions were broadly similar between microbiota community-types (L and O) (Additional file 1: Fig. S3). Although we found eosinophils to be higher in O- compared to L-type samples, 95% and 99% of O- and L-type samples in the training set and 91% and 100% of O- and L-type samples in the validation set contained no eosinophils. Moreover, eosinophil cell proportions were a poor predictor of microbiota community-type under a logistic regression model trained in the training set (ROC AUC in the validation set was 0.54, 95% CI = 0.51–0.58). The total immune cell proportion was also not associated with microbiota community-type (ROC AUC in the validation set was 0.53, 95% CI = 0.43–0.63).

**Fig. 1:**
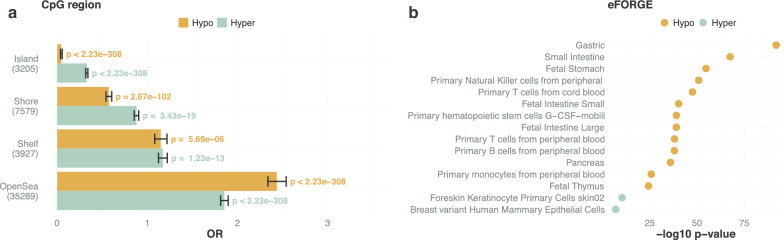
Enrichment of input CpG feature space. **a** Input feature pool enrichment for CpG region. **b** Input feature pool enrichment for cell-type (eFORGE). See “Methods” section for details. See also Additional file 1: Fig. S4

Using the training set, we assessed the number of CpGs which were significantly differentially methylated in samples classified as community-type L and community-type O, by applying a logistic regression model adjusted for age and proportion of immune cells (IC, see “Methods” section). After adjustment for multiple comparisons (with false discovery rate*, qvalue* R package, version 2.16.0), 173,245 CpGs showed a significant difference between L- and O-type samples; 109,500 were hyper- and 63,745 were hypo-methylated in O-type samples. From this ranked list, the optimum input pool size of features for a linear classifier, determined under a penalized logistic regression model (see “Methods” section), was significantly enriched for CpGs that were the furthest away from CpG islands with a considerable over-representation of open sea CpGs (Fig. 1a). We further utilized the eFORGE tool [17] in order to search for enrichment of cell-type specific CpGs in the top 1,000 hyper- and hypo-methylated CpGs. The strongest enrichment was observed in hypo-methylated CpGs for cells that are part of the gastrointestinal tract (Fig. 1b). This suggests that women with a predominant O-type microbiota exhibit a cervicovaginal epigenome reflective of gastrointestinal differentiation that is less supportive of Lactobacilli colonization. Applying gene set enrichment analysis (GSEA, see “Methods” section), hyper-methylated CpGs were enriched for cancer-associated terms (Additional file 1: Fig. S4).

**Fig. 2:**
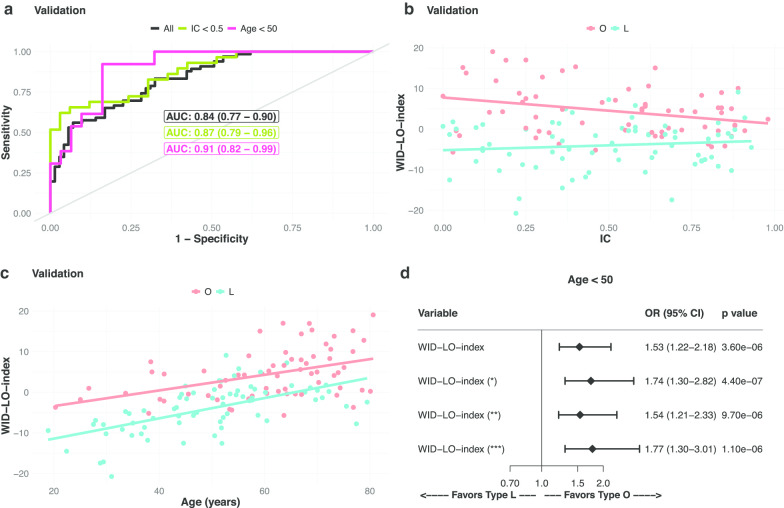
WID-LO-index performance in cervical samples. **a** Receiver operating characteristic curves for the linear classifier in the validation set. **b**, **c** WID-LO-index in the validation set, trend with IC (**b**) and with age (**c**). **d** Adjusted odds ratios for the association of WID-LO-index with community-type determined from sample microbiota proportions, Age < 50 years subgroup. See also Additional file 1: Fig. S7. (*) corresponds to adjustment for Age. (**) corresponds to adjustment for IC. (***) corresponds to adjustment for age and IC. Odds ratios, 95% confidence intervals and p values were calculated under a logistic regression model with a bias reduction method. IC = immune cell proportion. The WID-LO-index was generated with 819 selected CpGs. See “Methods” section for details

In order to derive a diagnostic DNAme signature, termed the WID-LO-index (Women’s risk IDentification Lactobacilli or Other index), we used elastic-net, ridge, and lasso generalized linear models to classify individuals as community-type L or O (see “Methods” section). The classifiers, which included only a linear combination of features, were trained on the training set, which was used for both the optimization of hyper-parameters and optimization of the pool size of input CpG under a cross-validation resampling strategy (Additional file 1: see Figs. S5, S6 and S7) and the ROC AUC was used as a measure of performance. Using the training set, the WID-LO-index was developed consisting of 819 CpGs (565 hyper- and 254 hypo-methylated), which were selected by elastic-net logistic regression (see “Methods” section). In the independent validation set (see Fig. 2a), the WID-LO-index achieved a performance of 0.84 (95% CI = 0.77–0.90). In samples with a high epithelial proportion (i.e., immune cell proportion < 0.5) the AUC was 0.87 (95% CI = 0.79–0.96, see Fig. 2a), and in those with a proportion ≥ 0.5 the AUC was 0.81 (95% CI = 0.71–0.91; Additional file 1: Fig. S8), suggesting that the main discriminatory signal originates from the epithelial component of the sample (Fig. 2b). The WID-LO-index was not associated with IC fraction in L-type samples (linear regression model − 5.2 + 2.4 × IC, *p*_IC_ = 0.36, Fig. 2b), but a significant negative trend was observed in O-type samples (linear regression model − 7.77 − 6.49 × IC, p_IC_ = 0.014, Fig. 2b). In both L- and O-type women, the WID-LO-index increased with age (WID-LO-index ~ − 16.41 + 0.25 × AGE, *p*_AGE_ = 3.89 × 10^–8^ for L-type and ~ − 7.24 + 0.19 × AGE, *p*_AGE_ = 1.85 × 10^–4^ for O-type, see Fig. 2c).

The performance of the WID-LO-index was slightly better in women < 50 years of age (AUC of 0.91, 95% CI = 0.82–0.99), compared to older women (AUC of 0.79, 95% CI = 0.70–0.88) (Figs. 2a and Additional file 1: S8), which is an extremely relevant result since the younger group is where prediction of LO-type is the most clinically relevant [4]. The association between LO-type and the WID-LO-index was stable after adjustment for age and IC proportion in both age-groups of women (Fig. 2d, see also Additional file 1: Fig. S9). Yet, the 819 group of CpGs comprising the WID-LO index did not show any enrichment for terms under an eFORGE or GSEA. Regarding the association of the WID-LO index with gene region and CpG region, the trend seen for the optimal input feature size (Fig. 1a and Additional file 1: Fig. S4b) is mostly maintained, although hyper-methylated CpG enrichment for shore is lost and for shelf both hyper- and hypo-methylation are lost (Additional file 1: Fig. S5a and c). Regarding enrichment for gene region, we observe that the WID-LO index set of CpGs loses some associations that were verified for the 50,000 CpG input feature set (Additional file 1: Fig. S5b and d). This was to be expected given the lower cardinality of the 819 CpG set.

**Fig. 3:**
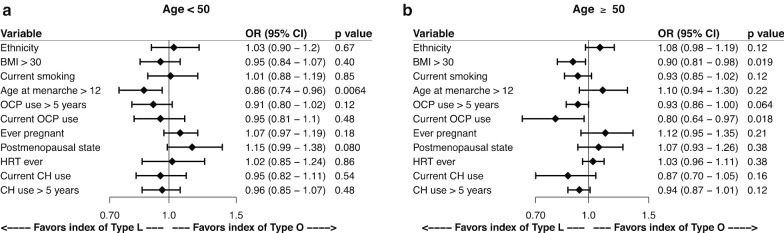
Association of the WID-LO-index with each of the additional covariates collected. **a** Age < 50 years. **b** Age ≥ 50 years. See “Methods” section for details. BMI = body mass index (kg/m^2^). OCP = oral contraceptive pill. HRT = hormone replacement therapy. CH = combined hormone. See also Additional file 1: Tables S5 and S6

Assessing the association between epidemiological factors and the WID-LO-index in all women (data not shown), we verified that if we adjust for those that show a significant association (age, postmenopausal state, age at menarche > 12, HRT ever, BMI, OCP use > 5 years, current OCP use, ever pregnant and current CH use), the WID-LO remains a good predictor of community-type (OR = 1.24, 95% CI = 1.12—1.38, p = 1.12 × 10^–6^). If we focus on age subgroups, i.e., in women < 50 (Fig. 3a) and ≥ 50 years of age (Fig. 3b) in the validation set, the same pattern is not verified but several significant associations still appear. In women < 50 years of age, a lower index was observed for women whose age at menarche was > 12 years. Adjusting for ‘age at menarche’, as we did for age and IC proportion (Fig. 2d), the WID-LO index was still a significant predictor of community-type (OR = 1.28, 95% CI = 1.17—1.42, p = 1.16 × 10^–11^). For women older than 50 years of age, a lower index was observed in women with a BMI > 30 or women who were still using the oral contraceptive pill, which potentially suggests that higher estrogen levels in women ≥ 50 years impact on the cervicovaginal epithelium epigenome that favors Lactobacilli colonization. In both age-groups, non-white women tended to have a higher WID-LO-index favoring O-type communities. The WID-LO-index also remained a significant predictor of community-type in women ≥ 50 years after adjustment for ‘BMI’ and ‘current OCP use’ (OR of 1.24 (95% CI = 1.12–1.41), *p* = 2.73 × 10^–6^).

**Fig. 4:**
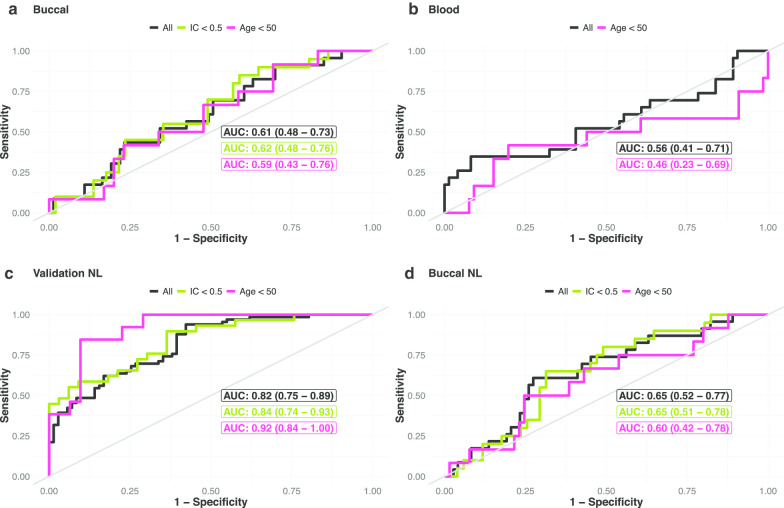
Validation in buccal and blood samples. **a**, **b** Receiver operating characteristic curves for the linear WID-LO-index classifier in buccal (**a**) and blood samples (**b**). **c**, **d** Receiver operating characteristic curves for the NL WID-LO-index, in the validation set (**c**) and in buccal samples (**d**). See also Additional file 1: Figs. S10, S11, S12 and S13. The WID-LO-index was generated with 819 CpGs. The NL WID-LO-index was generated with 1,162 features (**c**, **d**). IC = immune cell proportion. See “Methods” section for details

We next sought to assess whether the epigenetic signature, which was derived in cervical smear samples, is also able to correctly classify the cervicovaginal L- or O-type microbiome when analyzed in cells from other anatomical regions. We analyzed the WID-LO-index in buccal (Fig. 4a) and blood (Fig. 4b) samples of 96 women (ages ranging from 18.7 to 69.3 years, median of 38.45 years). As these women were younger, the L-type was more prevalent (76% L-type, 24% O-type) compared to the group of women in the training (52% L-type, 48% O-type) and validation sets (52% L-type, 48% O-type). Whereas in the blood samples the index was a poor predictor (AUC of 0.56, 95% CI = 0.41–0.71), there was a trend in buccal samples (AUC of 0.61, 95% CI = 0.48–0.73), albeit insignificant. This was more pronounced in samples with a low IC proportion (Fig. 4a, see also Additional file 1: Fig. S10).

In defining the WID-LO-index, we included only those CpGs that remained significant after a logistic regression adjusted for age and IC proportion. We did not, however, consider other methods with which to identify informative CpGs such as those associated with outliers, which we have demonstrated to provide independent information [18, 19] and potentially could capture further systemic epigenetic alterations. Hence, we revisited the training set, applied various ranking algorithms (see “Methods” section) and took the geometric mean across the ranked lists. This final ordered pool was used to once again apply the same three penalized regression strategies, but with high-order terms included (see “Methods” section). This independent classifier called nonlinear (NL) WID-LO-index, which provides a similar performance in the cervicovaginal samples of the validation set (see Fig. 4c, Additional file 1: Figs. S11 and S12), was also able to better discriminate the cervicovaginal L- and O-type based on buccal samples (AUC of 0.65, 95% CI = 0.52–0.77, see Fig. 4d, Additional file 1: Fig. S13a, b and c). Again, this was not the case in peripheral blood samples (Additional file 1: see Fig. S13d, e and f).

## Discussion

Most studies exploring the epigenome–microbiome interaction have focused on the gut and have shown, to an extent, that the microbiome influences the host’s local epigenetics and intestinal homeostasis [20]. Whether microbial signals directly affect and independently regulate the epigenome through DNA methylation and histone modification [21] has not been completely clarified. Here, we approached this subject from a clinical perspective and looked at systemic epigenetic alterations in different tissues. We developed a linear methylation index (WID-LO-index) in cervicovaginal samples that predict the prevalent type of microbial communities. Although we did not explore the molecular mechanisms driving the epigenome–microbiome strong local interaction, we showed, with a nonlinear index based on the same principles of the WID-LO-index, that the predictive methylation signal identified in cervicovaginal samples is present in a completely different and unrelated region, which is naturally subjected to different local stressors. This finding enhances the likelihood of a potential underlying systemic causal link, possibly shaped by environmental and hormonal factors, with the epigenome having a clear role in shifting the host cell differentiation toward an environment that facilitates the growth of lactobacilli or non-lactobacilli community-types. In order to test the direct causal relationship between each of the variables identified in our study, a clinical longitudinal assessment is necessary to ascertain whether the presence of certain methylation patterns favors the growth of specific microbial communities. Clarification of this causal network is fundamental to establishing under what circumstances intervention at the level of the microbiome is successful [1, 22, 23] by reinstating protective lactobacilli species, and whether it could reduce the risk of diseases which are associated with an abnormal vaginal microbiome.

Recent data in mice demonstrated that the microbiome impacts on the DNA methylome of the host [20]. Future research will need to assess further the interaction between the epigenome and the microbiome in order to find novel strategies for disease prevention.

## Conclusions

We have demonstrated that a strong interaction exists between the microbiome and the epigenome. Our data are consistent with the view that ethnical, developmental and environmental factors contribute to epigenetic alterations in the host that might lead to subtle changes in cell identity and function, which, eventually, could favor the growth of a specific microbiome community-type. Our findings highlight that the host epigenome is centrally involved in facilitating growth of specific microbial communities. Our results also show that future studies should consider the host epigenome when assessing resistance mechanisms—this is of particular importance when considering individuals who do not adequately respond to microbiota transplantation [23].

## Methods

### Study design and participants

This work was conducted as part of a multicenter study (the FORECEE [4C] Program) involving several recruitment sites in five European countries (i.e., the UK, Czech Republic, Italy, Norway and Germany) [4]. Participants were aged > 18 years. Prior to taking part, each prospective study volunteer was given a participant information sheet as well as a consent form, and the rationale for the study was explained. Additional resources, including an explanatory video and further online resources, were also made available. Women were approached during outpatient hospital clinics or via outreach campaigns and public engagement. Prospective participants completed an epidemiological questionnaire, as well as a feedback form after their participation [4]. For further details, e.g., vaginal sample collection and transportation, sample matching, the consent process and wet laboratory processing of cervicovaginal samples, we refer the reader to the previously published work [4]. For information pertaining to any of the covariates characterizing the samples in the training and validation sets, see Additional file 1: Tables S1–S4.

### DNA extraction and 16S rRNA amplicon sequencing

Total DNA extraction from cervical swabs was performed with the QIAsymphony DSP Virus/Pathogen kit according to the manufacturer’s instructions (Qiagen, Hilden Germany). Sequencing and taxonomical classification of bacterial species in the cervical swab samples were performed by Eurofins Genomics Europe Sequencing (Constance, Germany) following a formerly described approach [24]. Further details related to this section are provided in great detail in previously published work, including fundamental settings for reproducibility [4].

### Processing of 16S sequencing data

The demultiplexed sequencing reads were quality checked, trimmed and filtered (Sickle v1.33 [25]) and adapters and primers removed (Cutadapt v1.10 [26]). Overlapping paired-end reads were merged for full 12 length V1–V3 16S amplicons (FLASh v1.2.11) [27], clustered (CD-HIT v4.6) [28], and chimeric sequences removed (UCHIME v4.2.40) [29]. Operational taxonomic units (OTUs) were assigned with BLASTN+ (v2.4.0) [30] via a non-redundant 16S rRNA reference database from the Ribosomal Database Project (RDP, Release 11) [31] and filtered for high quality. Taxonomic classification was based on the NCBI Taxonomy [32]. Please check previously published work for further details [4].

### Clinical/reproductive and microbial data

The choice of the threshold of 50 years for the division of the cohort into separate age-groups in Fig. 3 follows the rationale previously reported [4]. This value was determined to be between the upper limit for the recruitment of volunteers, 45 years [12] and 55.2 years, the latter value was determined by fitting a univariate logistic regression model to controls with community-type as the response variable and age as the sole predictor. Receiver operator characteristic (ROC) curve analysis and the Youden’s J statistic were used to calculate the optimal age threshold [4]. In this work, the classifiers for community-type generated from the methylation matrices were, nevertheless, trained without dividing the training set into age subgroups.

Regarding the separation of cervicovaginal samples into dominant microbiota community-types, we followed the same rationale as that outlined in [4]. We collapsed four Lactobacillus community groups, previously identified by Ravel and colleagues (groups I, II, III and V [12]) into one microbial community, seen as the prevalent community in a ‘healthy’ microbiome. This community, which we referred to as community-type L, is comprised of four types of Lactobacillus, *L crispatus*, *L gasseri*, *L iners*, and *L jensenii*, and they are associated with a substantially lower vaginal pH, which has the potential to reduce the risk of ascending infections. In contrast to this community-type, we used an additional one (group IV in [12]), here referred to as community-type O, containing higher proportions of typical obligate and facultative anaerobe genera (such as Gardnerella or Atopobium species), which are associated with aerobic vaginitis and bacterial vaginosis [12] and are highly diverse. The loss of Lactobacillus species, which utilize glycogen, deposited at high levels in vaginal epithelium cells by estrogen action, has been reported to be associated with a reduction in estrogen concentrations in postmenopausal women [12, 33–35].

We divided the samples used in our analysis into people whose cervicovaginal microbiota consisted of at least 50% community-type L and those whose microbiota consisted of less than 50% community-type L (community-type O). A heatmap showing the abundance patterns across all subjects in the training is shown in Additional file 1: Fig. S2.

### Preparation of samples and DNA methylation analysis

DNA was isolated from cervical, buccal and blood cells using AllPrep DNA/RNA Mini Kits (#80204, Qiagen Ltd), following the manufacturer’s protocol. DNA concentration and quality absorbance ratios were measured using a Nanodrop-8000 (Thermo Scientific Inc). Extracted DNA was stored at -80˚C until further analysis. DNA was normalized to 25 ng/µl, and 500 ng total DNA was bisulfite-modified using the EZ-96 DNA Methylation-Lightning kit (Zymo Research Corp, cat #D5047) on a Hamilton Star Liquid handling platform. Eight microliters of modified DNA was subjected to methylation analysis on the Illumina Infinium MethylationEPIC BeadChip microarray (Illumina, CA, USA) at UCL Genomics according to the manufacturer’s standard protocol.

### Methylation data preprocessing

All methylation microarray data were processed through the same standardized pipeline. Raw data were loaded using the R package *minfi*. Any samples with median methylated and unmethylated intensities < 9.5 were removed. Any probes with a detection *p* value > 0.01 were regarded as failed. Any samples with > 10% failed probes and any probes with > 10% failure rate were removed from the dataset. Beta values from failed probes (approximately 0.001% of the dataset) were imputed using the *impute.knn* function as part of the *impute* R package.

Non-CpG probes (2932), SNP-related probes as identified by Zhou et.al. [36] (82,108), and chromosome Y probes were removed from the dataset. An additional 6,102 previously identified probes that followed a trimodal methylation pattern characteristic of an underlying SNP were removed.

Background intensity correction and dye bias correction were performed using the *minfi* single sample *preprocessNoob* function. Probe bias correction was performed using the beta mixture quantile normalization (BMIQ) algorithm.

The fraction of immune cell contamination and the relative proportions of different immune cell subtypes in each sample were estimated using the EpiDISH [16] algorithm using the epithelial, fibroblast and immune cell reference datasets. The top 1000 most variable probes (ranked by standard deviation) were used in a principal component analysis. Statistical tests were performed in order to identify any anomalous associations between plate, sentrix position, date of array processing, date of DNA creation, study center, immune contamination fraction, age, type (case versus control), and the top ten principal components.

### Statistical analysis

For the work presented here, we used samples (n = 448) from subjects for which both microbiome and methylation data were available for the case–control study previously reported [4]. We divided this joint set into a training (2/3) and validation (1/3) set, by stratifying by age, immune cell proportion (determined by EpiDISH [16]), and community-type (L or O). The resulting distributions can be seen in Additional file 1: Fig. S1. In order to evaluate the association between each of the clinical covariates and the classification type of microbiota in each sample, we resorted to the logistic regression model implemented in the *logistf* R package (version 1.23). This approach fits a logistic regression model using Firth’s bias reduction method. The reported confidence intervals and tests were based on the profile-penalized log likelihood and incorporate the ability to perform tests where contingency tables are asymmetric or contain zeros. This was used to evaluate the association between each covariate and the community-type determined from sequencing data (Additional file 1: Tables S1–S4), as was the case for work previously reported [4], as well as the index determined from the methylation patterns associated with each sample (Additional file 1: Tables S5 and S6). The missing values for epidemiological or clinical covariates were omitted in each independent fitting, but not across the whole study as they amounted to a small percentage for each variable (Additional file 1: see Table S2).

The linear WID-LO-index was developed by combining a ranking method, based on a logistic regression model adjusted for age and estimated immune cell fraction, of the CpGs associated with community-type and an elastic-net regularization path for logistic regression approach for feature extraction (*glmnet* R package, version 2.0.18). The adjustment for age and IC is fundamental while ranking CpGs; age and menopausal status are the two strongest associations with community-type, in the training set, *p* = 1.64 × 10^–10^ and 1.38 × 10^–11^, respectively (see also Additional file 1: Tables S1, S3 and S4).

The best classifiers were determined by scanning the ranked list of CpGs (from top to lower rank) and gradually adding a larger input pool of features. For each pool size, we tested 11 values for the *glmnet* hyper-parameter *α*, ranging from 0 to 1. For the hyper-parameter *λ*, we followed the default settings of the package. From the performance profile in the training set, we chose the best with a tenfold cross-validation resampling algorithm (see, for example, Additional file 1: Fig. S6). The best performance during cross-validation was achieved with a pool of 50,000 input CpGs (35,186 hyper- and 14,814 hypo-methylated), which resulted in only 819 having nonzero regression coefficients (*glmnet* hyper-parameter *α* = 0.3, see also Additional file 1: Fig. S6b). The model with 819 selected CpGs (see Additional file 1: Table S7) was the one tested in the validation set and in the buccal and blood samples.

We also developed a nonlinear index, here referred to as NL WID-LO-index, which includes a differently ranked pool of input CpGs; in this case, the ranks were calculated by the geometric means of the ranks found by five ranking methods, i.e., Welch’s test, Bartlett’s test, adjusted logistic regression test (Age + IC), Δ*β* method test (described below) and the CellDMC test [37]. The geometric mean of all five methods provided a measure of consistency highlighting different distinguishing features, from association with epithelial or immune cells (CellDMC test [37]) to differential variability between community-types (Bartlett’s test). In addition to the methylation values, this classifier also incorporates nonlinear terms of second order characterized by the product of the original *β* methylation values and the estimated immune cell proportion calculated with EpiDISH [16] for each subject. A similar parameter scanning strategy used to select the optimal linear classifier described above was also employed for the nonlinear case, i.e., the NL WID-LO-index.

The WID-LO-index and the NL WID-LO-index share 573 CpGs associated with first-order terms. The NL WID-LO-index uses 1,162 features (from an optimal input space of 60,000 unique CpGs plus nonlinear terms, with *α* = 0.2) of which 104 are higher-order terms with 51 involving CpGs that are not included in the linear terms. Thirty-seven of the 104 CpGs, which are included in higher-order terms in the NL WID-LO-index, are also present in the WID-LO-index. The model with 1,162 selected features (detailed signature not provided), which includes 1,109 unique CpGs, i.e., certain CpGs are used both in linear and in nonlinear terms, was the one tested in the validation set and in the buccal samples (see, for example, Fig. 4c, d).

The AUC for the ROC curves was used as the performance metric. ROC curves were generated with the *pROC* R package (version 1.15.3). 95% CI for AUCs were determined by stratified bootstrapping (DeLong’s method).

The Δ*β* ranking method identifies CpGs associated with a signal stemming from epithelial cells by ranking them according to |Δ*β*|. Δ*β* corresponds to the difference in methylation, for a specific CpG, between the y-intercepts at IC = 0 for linear regression models generated for the community-type O subgroup and the community-type L subgroup, independently.

The forest plots presented in Figs. 2d, 3a and b, and Additional file 1: Fig. S9 were created with the *forestplot* R package (version 1.9).

The abundance patterns plotted in Additional file 1: Fig. S2 within each age-group and each microbiota community-type were clustered by a hierarchical clustering algorithm, *hclust*, in R, by employing the Ward’s method. The patterns were scaled column-wise. The species selected for the heatmaps correspond to those belonging to community-type L, i.e., *L crispatus*, *L iners*, *L gasseri*, or *L jensenii*, in addition to those that ranked highest in terms of average. For all species analyzed, please see the data availability statement.

For considerations related to power calculation, please see details in the work previously published [4].

## Supplementary information

**Additional file 1.** Supplementary figures and tables addressing stratification of training and validation sets, overall species abundance per sample, cell type proportion differences between subjects, optimization and performance of classifiers, gene set enrichment analysis, eFORGE analysis and CpGs comprising the WID-LO-index.

## Data Availability

The original data, including all clinical, epidemiological, microbiome, and methylation data used in this work are not yet publicly available as this is part of a larger study; those data which are not identifying individuals will become available via the European Genome-phenome Archive after signing a data access agreement. All packages used in the preprocessing of data and subsequent analysis have been identified in order to secure full reproducibility.
