# Supplementary material for: DNA methylation signatures to predict the cervicovaginal microbiome status
Source: Clin Epigenetics. 2020 Nov 23;12:180. doi: 10.1186/s13148-020-00966-7 (PMC7686703; doi:10.1186/s13148-020-00966-7)
Supplement: Supplementary file 1 — Additional file 1. Supplementary figures and tables addressing stratification of training and validation sets, overall species abundance per sample, cell type proportion differences between subjects, optimization and performance of classifiers, gene set enrichment analysis, eFORGE analysis and CpGs comprising the WID-LO-index. [file 13148_2020_966_MOESM1_ESM.docx]

Supplementary information

**DNA methylation signatures to predict the cervicovaginal microbiome status**

Nuno R. Nené, James Barrett, Allison Jones, Iona Evans, Daniel Reisel, John F. Timms, Tobias Paprotka, Andreas Leimbach, Dorella Franchi, Nicoletta Colombo, Line Bjorge, Michal Zikan, David Cibula, Martin Widschwendter

In this supplementary information we provide the additional tables and figures cited in the main text.

**Supplementary Figures**

**Fig. S1 Distribution of subjects in the training and validation sets according to microbiota community-type L/O. a, d, g,** Contour plots for the distribution of subjects with age and immune cell proportion for all subjects (**a**), the training set (**d**) and the validation set (**g**). **b, e, h,** Density with age and community-type for all subjects (**b**), the training set (**e**) and the validation set (**h**). **c, f, i,** Density with immune cell proportion and type for all subjects (**c**), the training set (**f**) and the validation set (**i**). In addition to the histograms and the fitted distributions in the density plots, the mean for each community-type (L or O) distribution is also provided (dashed lines in b, c, e, f, h, i). IC= Immune cell proportion.


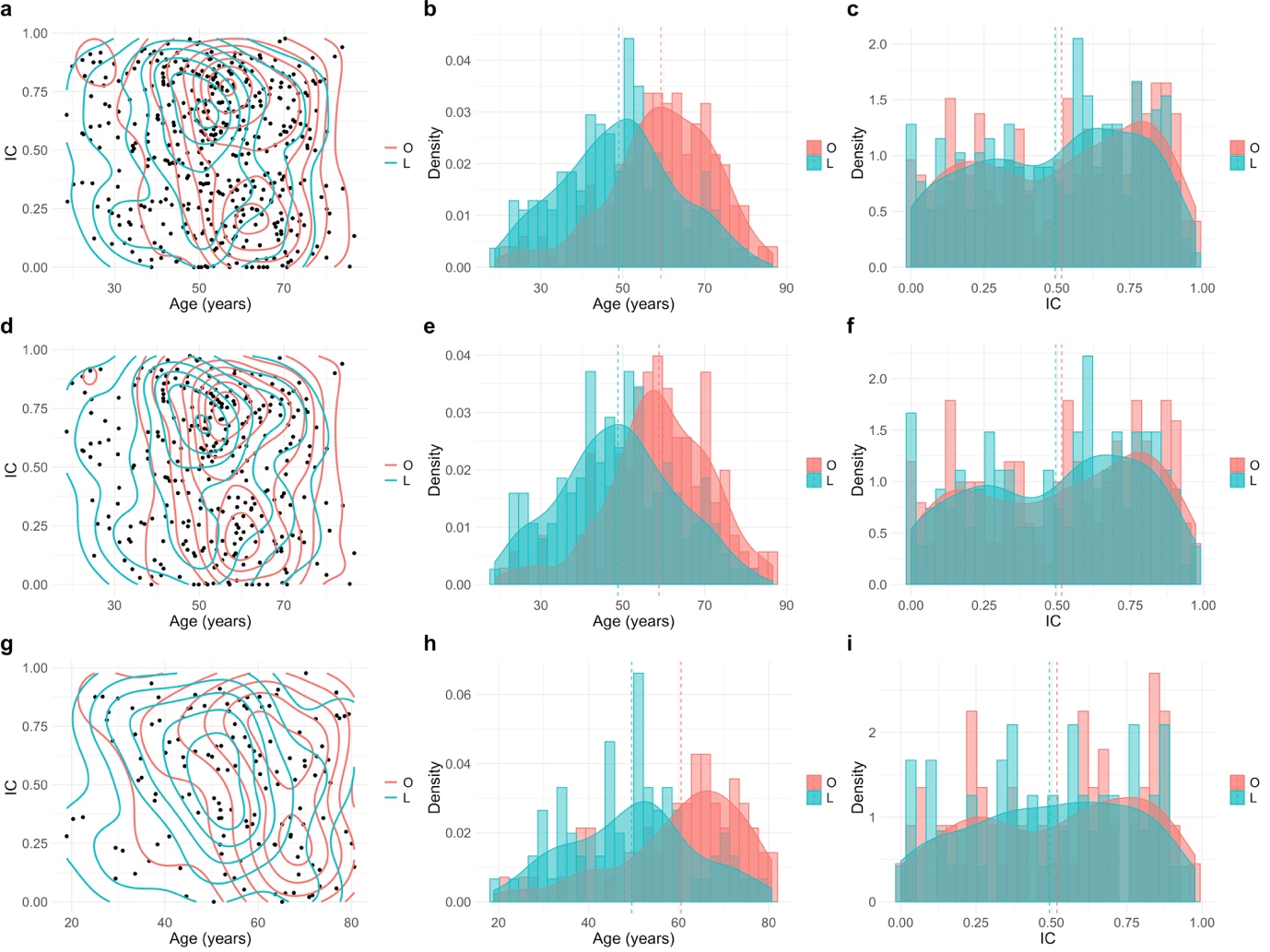


**Fig. S2 Overall species abundance per subject for the training set.** Heatmap grayscale (see lefthand side) is proportional to abundance in each subject. Abundances have been column-wise normalized for the purpose of enhancing the underlying patterns. The legend represents membership to community-types L or O. <50 and ≥ 50 indicates the age bracket of the individuals, younger or older than 50 years. Here we plot only the top abundant species. For the purposes of developing a classifier, samples were divided into women whose cervicovaginal microbiota consisted of at least 50% community type L (*L. crispatus*, *L. iners*, *L. gasseri* and *L. jensenii,* highlighted in red) and those whose microbiota consisted of less than 50% community type L, which we referred to as community type O. For the full list of species making community-type O, see the data availability statement.


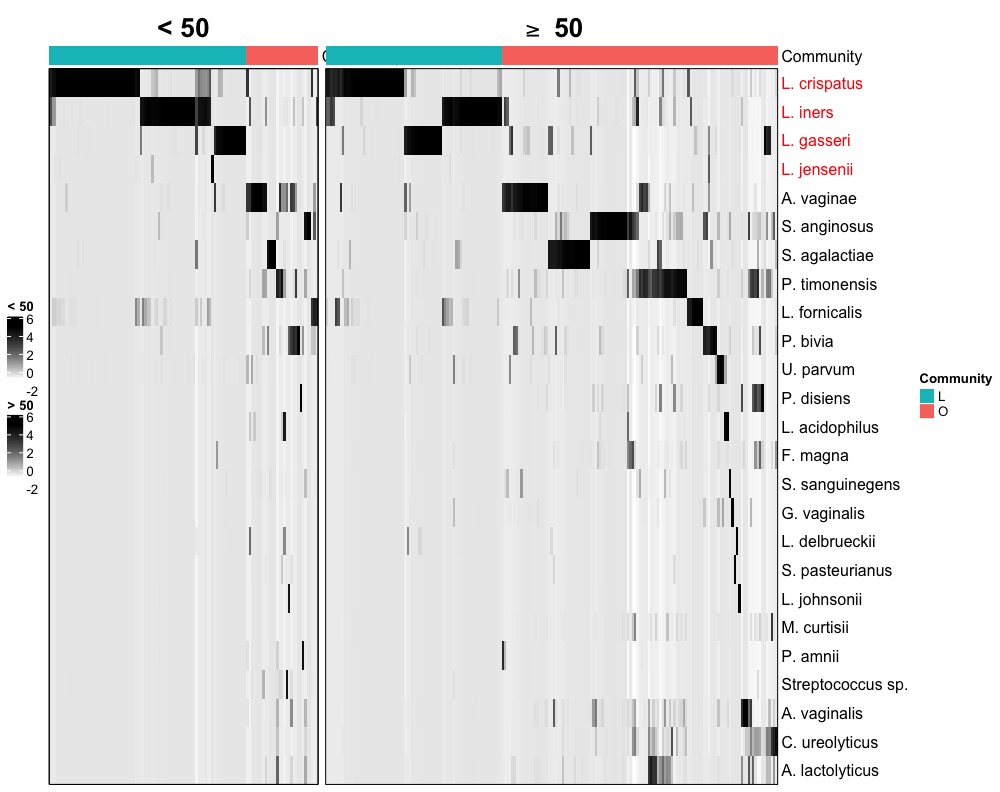


**Fig. S3 Cell type proportion differences between subjects with community-type. a,** Training set. **b,** Validation set. **c,** Buccal samples. **d,** Blood samples. Cell proportions were determined with EpiDISH (see Methods). Significant differences between the distributions for each community-type (L or O) are identified by * (p < 0.05) and ** (p < 0.01). P values were calculated with a Wilcoxon-test. Epi- epithelial cells. Fib- fibroblasts. B- B lymphocytes. NK- natural killer cells. CD4T-T helper cells. CD8T- T killer cells. Mono- monocytes. Neutro- neutrophils. Eosino- Eosinophils.


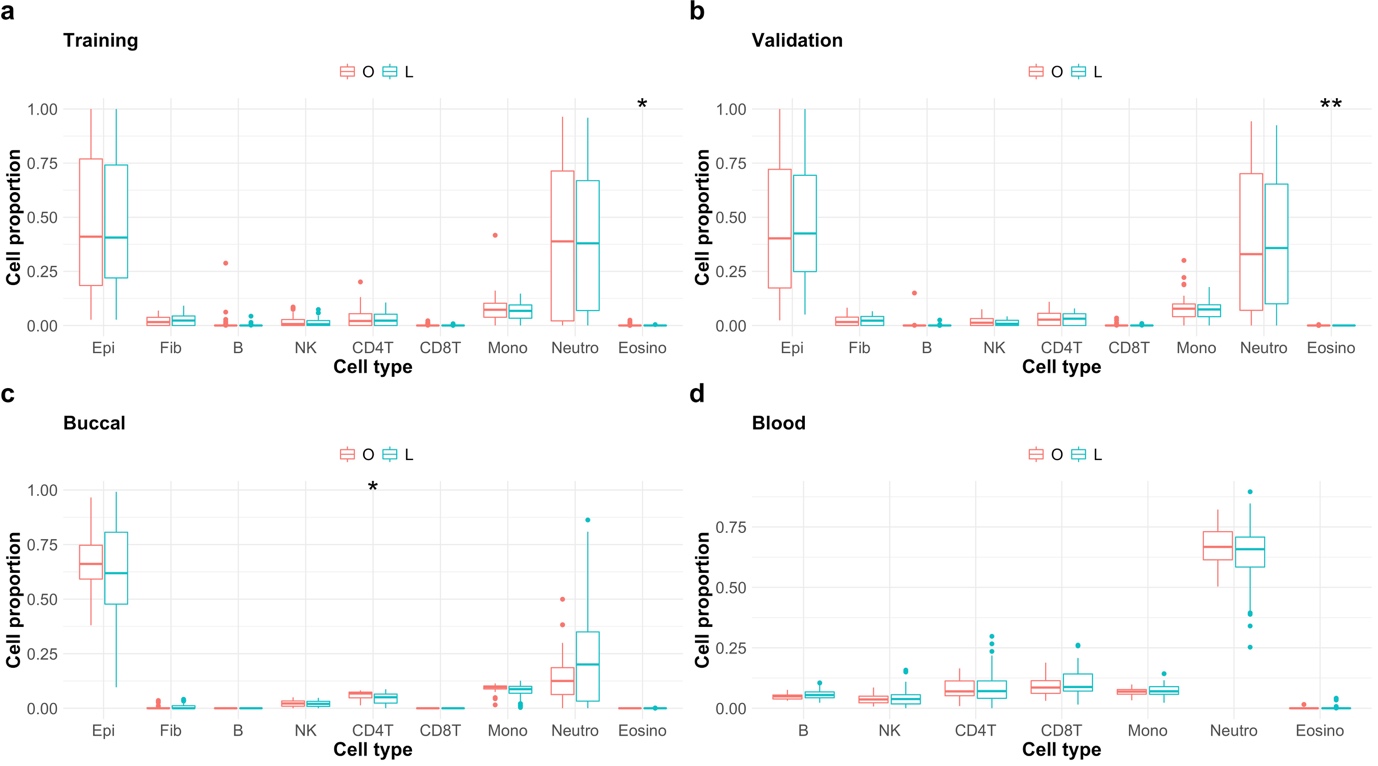


**Fig. S4 Association of top CpGs with gene region and gene set enrichment analysis, linear classifier (WID-LO-index). a, b,** Association of top 50,000 CpGs (absolute numbers) ranked according to a logistic regression model adjusted for age and immune cell proportion with gene region (**a**) and CpG region (**b**). This was the optimum number of input CpGs for the best classifier. **c,** Odds ratios for (**a**), where the p-values were calculated via the exact Fisher method (two-sided) against the proportions in the entirety of the Illumina InfiniumMethylation EPIC BeadChip array. **d**, Gene enrichment analysis for the same 50,000 CpGs, performed with an empirical Bayes algorithm in the *ChAMP* R package (version 2.14.0). Best classifier in the training set was the Elastic Net, with 819 selected CpGs from a pool of 50,000 input CpGs, with α=0.3 (see also Fig. S6 and Table S7).


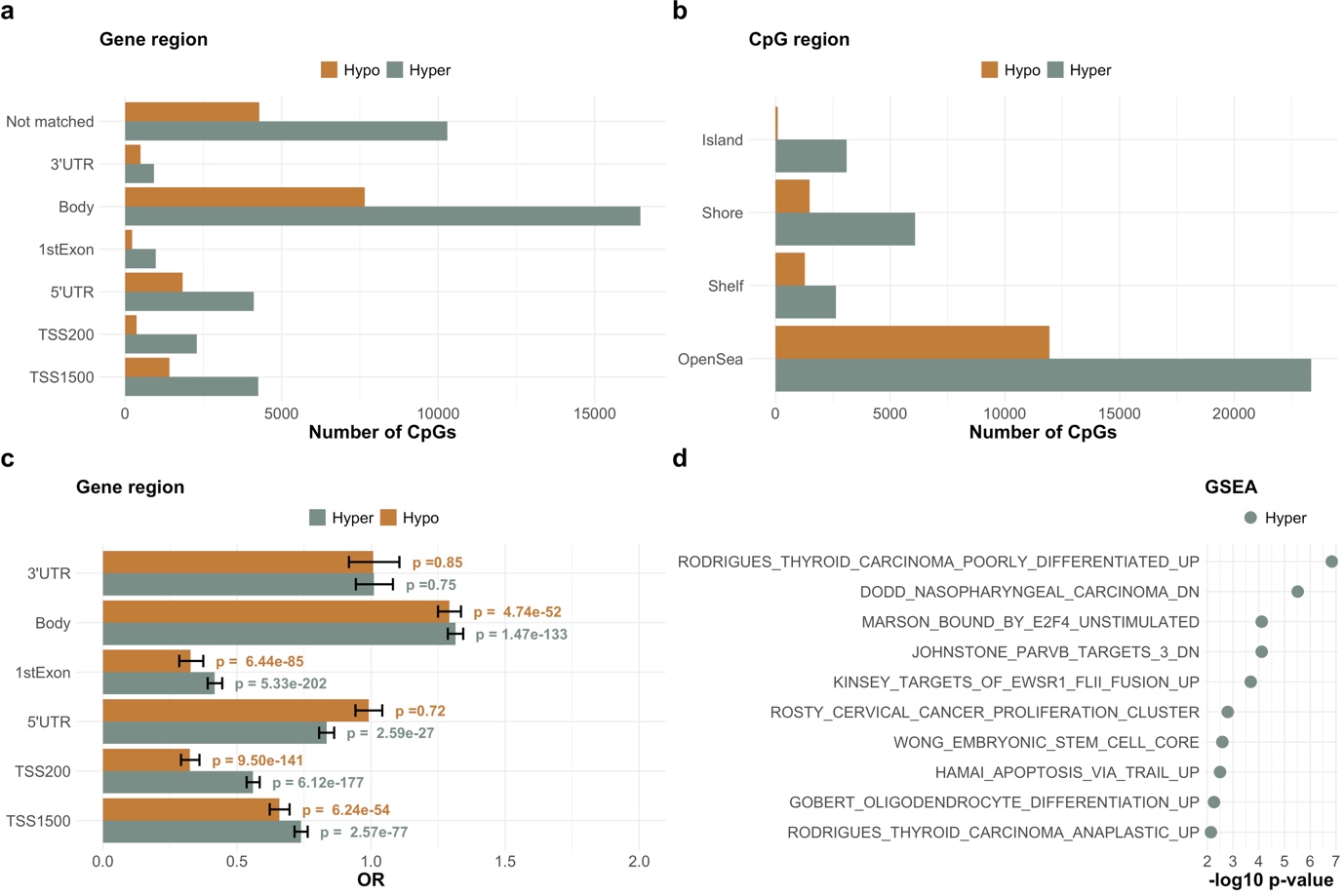


**Fig. S5 Association of the linear WID-LO-index 819 CpGs with CpG and gene region. a,** GpG region. **b,** gene region (**b**). **c,** Odds ratios for (**a**), where the p-values were calculated via the exact Fisher method (two-sided) against the proportions in the entirety of the Illumina InfiniumMethylation EPIC BeadChip array. **d**, Odds ratios for (**b**). See also Fig. S6 and Table S7.

**
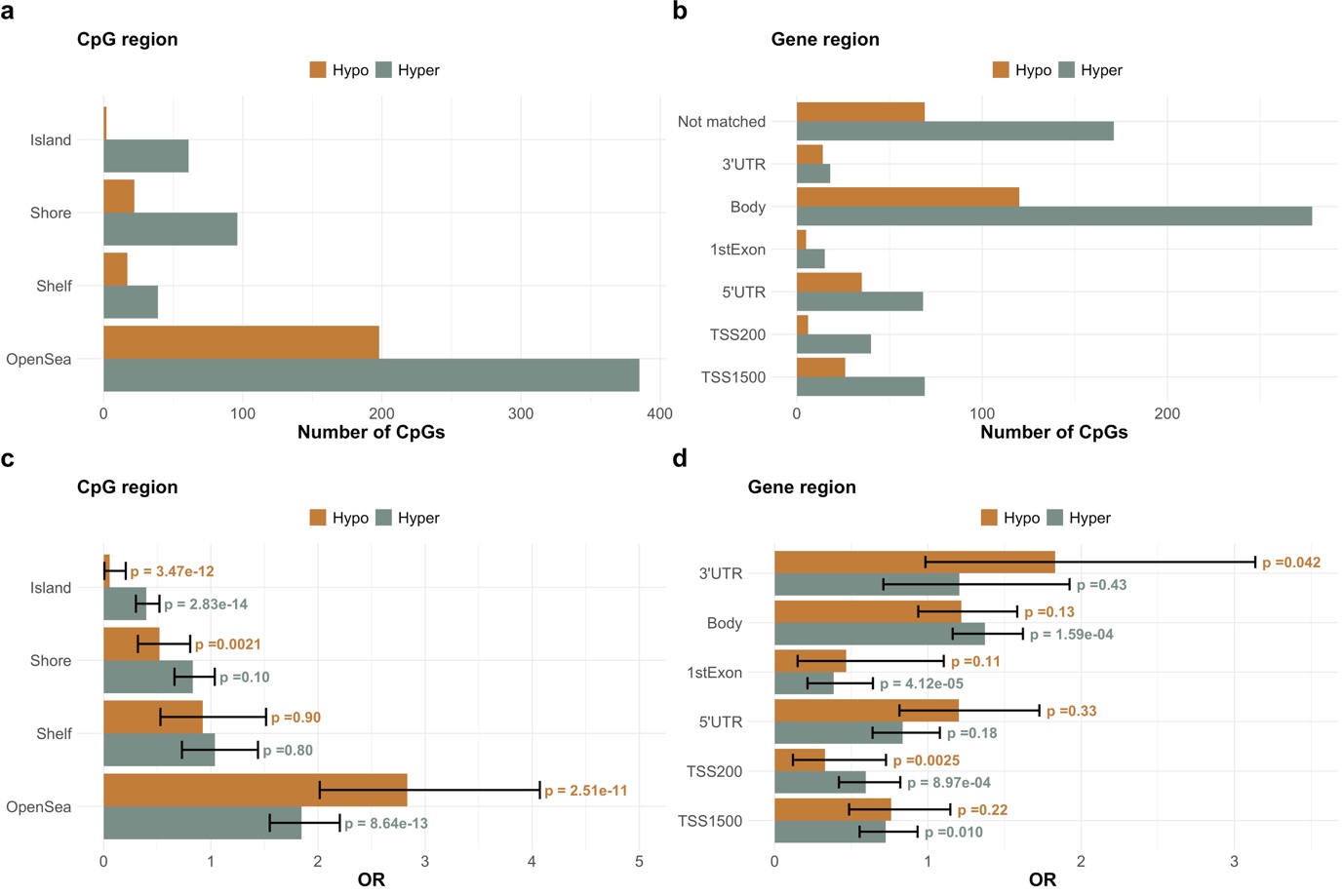
**

**Fig. S6 Performance profile of the linear classifier (WID-LO-index) in the training set. a,** Average performance across training folds. Best classifier in the training set was the Elastic Net (ElNet), with an input pool of 50,000 CpGs, hyperparameter α=0.3, resulting in 819 non-zero coefficients (selected CpGs). For comparison purposes, we provide the Ridge and Lasso regression results. **b,** Number of selected CpGs at each input pool size. Best performer displayed in (**a**) has 819 selected CpGs (indicated). Due to the discrepancy between the number of selected CpGs in ElNet, Lasso and Ridge, we only display the first two algorithms in (**b**). See also Methods section in the main text and Table S7.


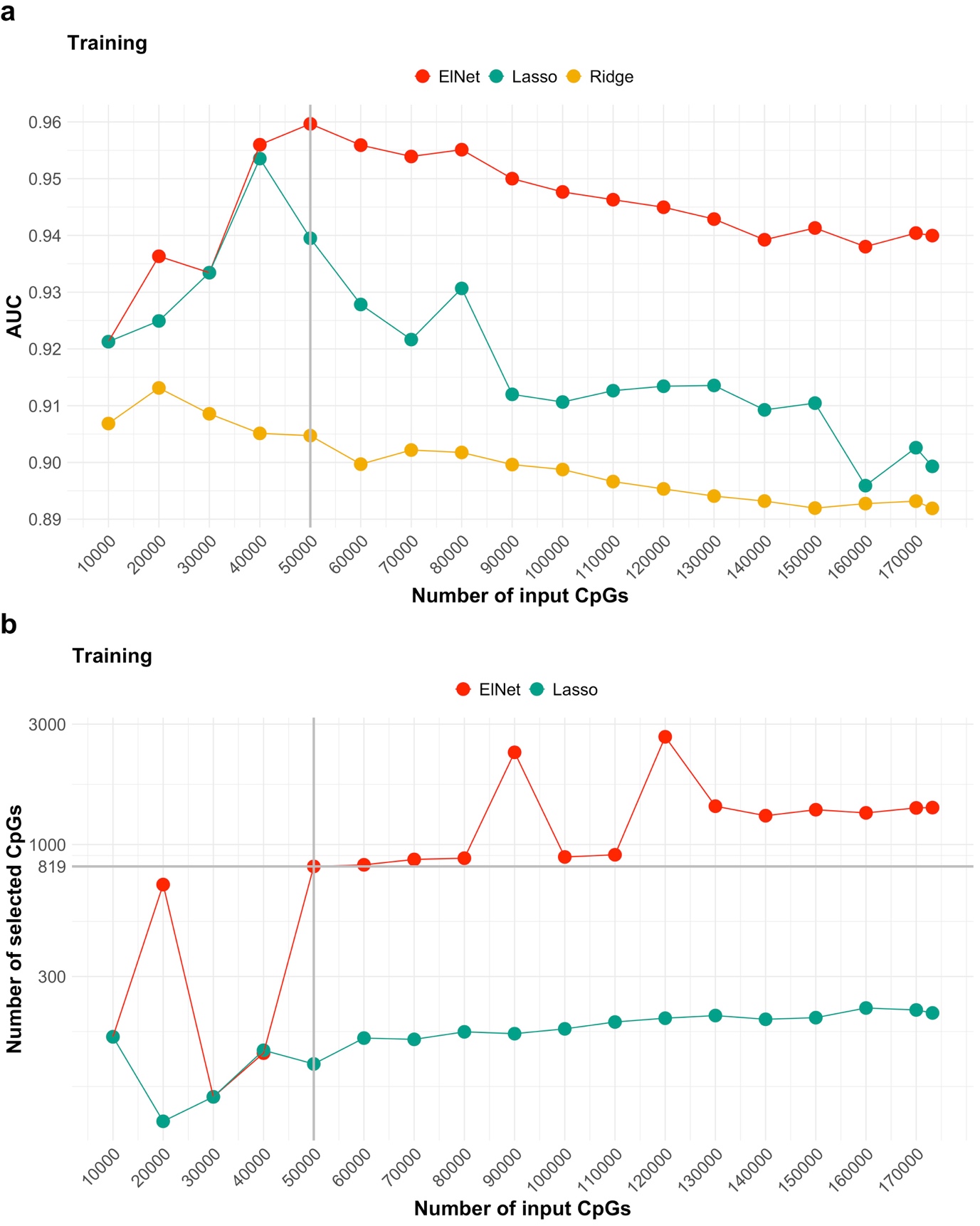


**Fig. S7 WID-LO-index in the training set. a,** Linear index scatter plot with IC. **b,** Linear index scatter plot with Age. See also Fig. 2 in the main text. This linear classifier involves 819 CpGs. See also Fig. S6 and Table S7. IC=Immune Cell proportion.


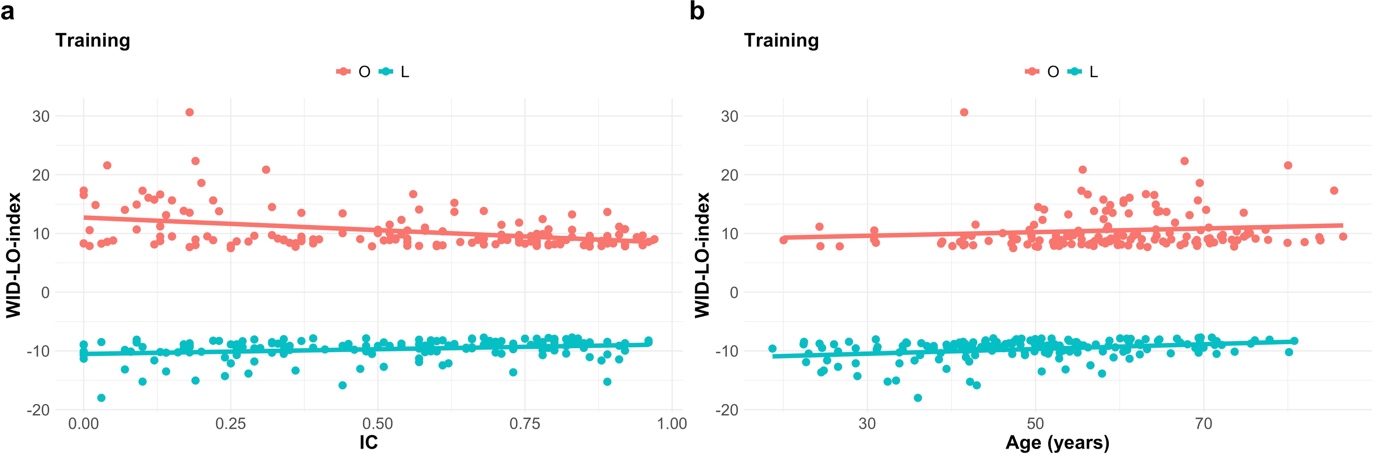


**Fig. S8 Performance of the WID-LO-index in the validation set.** This linear classifier involves 819 CpGs. See also Fig. S6 and Table S7. IC=Immune Cell proportion.


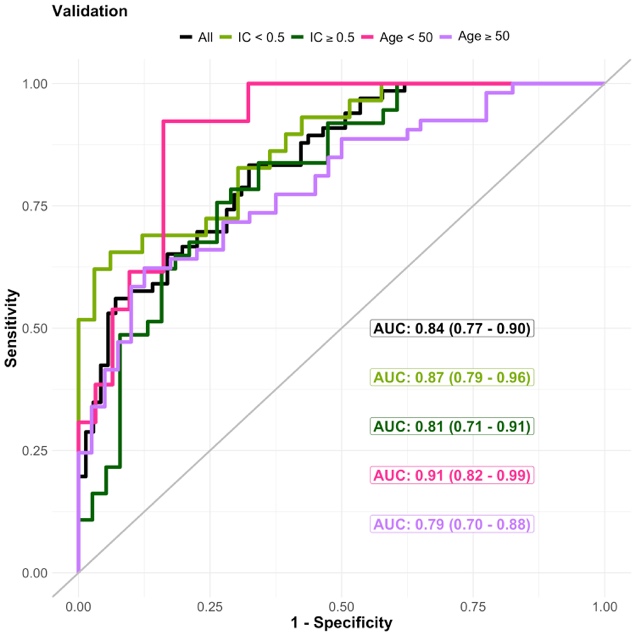


**Fig. S9 Odds-ratios and p-values for the association between community-type LO and the WID-LO-index. a,** All ages. **b,** Age ≥ 50 (years). See also Tables S5 and S6 for the epidemiological covariates. (*) corresponds to adjustment for Age. (**) corresponds to adjustment for IC. (***) corresponds to adjustment for Age and IC. IC- Immune Cell proportion. Odds-ratios, 95% Confidence Intervals and p values were calculated under a logistic regression model with a bias reduction method. See also Fig. 2d for results in the sub-group Age < 50 years. This linear classifier involves 819 CpGs. See also Fig. S6 and Table S7.

**
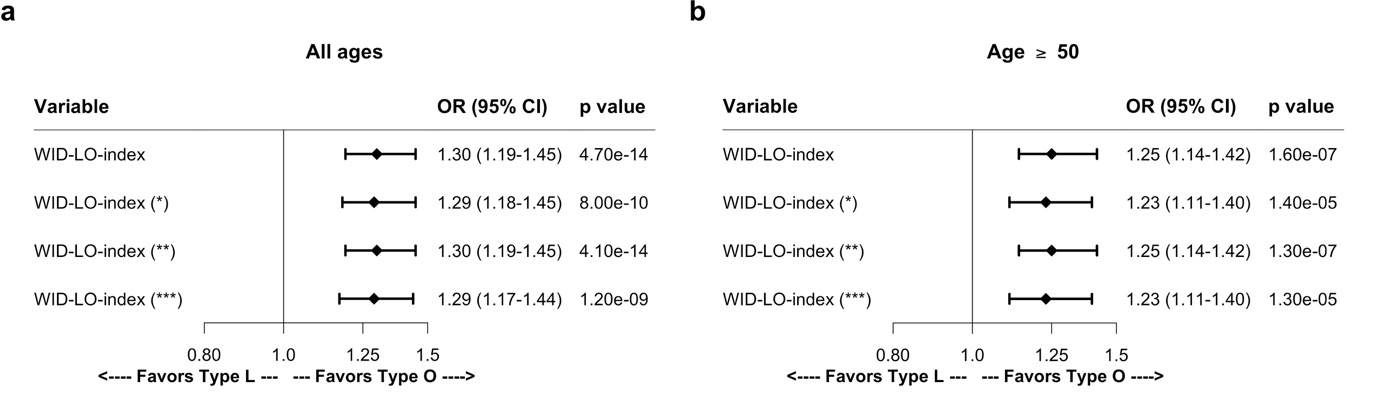
**

**Fig. S10 WID-LO-index for Buccal and Blood samples. a,** Performance in buccal samples. **b,** **c,** Index in buccal samples with IC (**b)** and Age (**c). d,** Performance in blood samples versus granulocyte proportion. **e, f,** Index in blood samples versus IC (**e**) and Age (**f**). See also Fig. 4 in the main text. This linear classifier involves 819 CpGs. See also Fig. S6 and Table S7. IC=Immune Cell proportion.


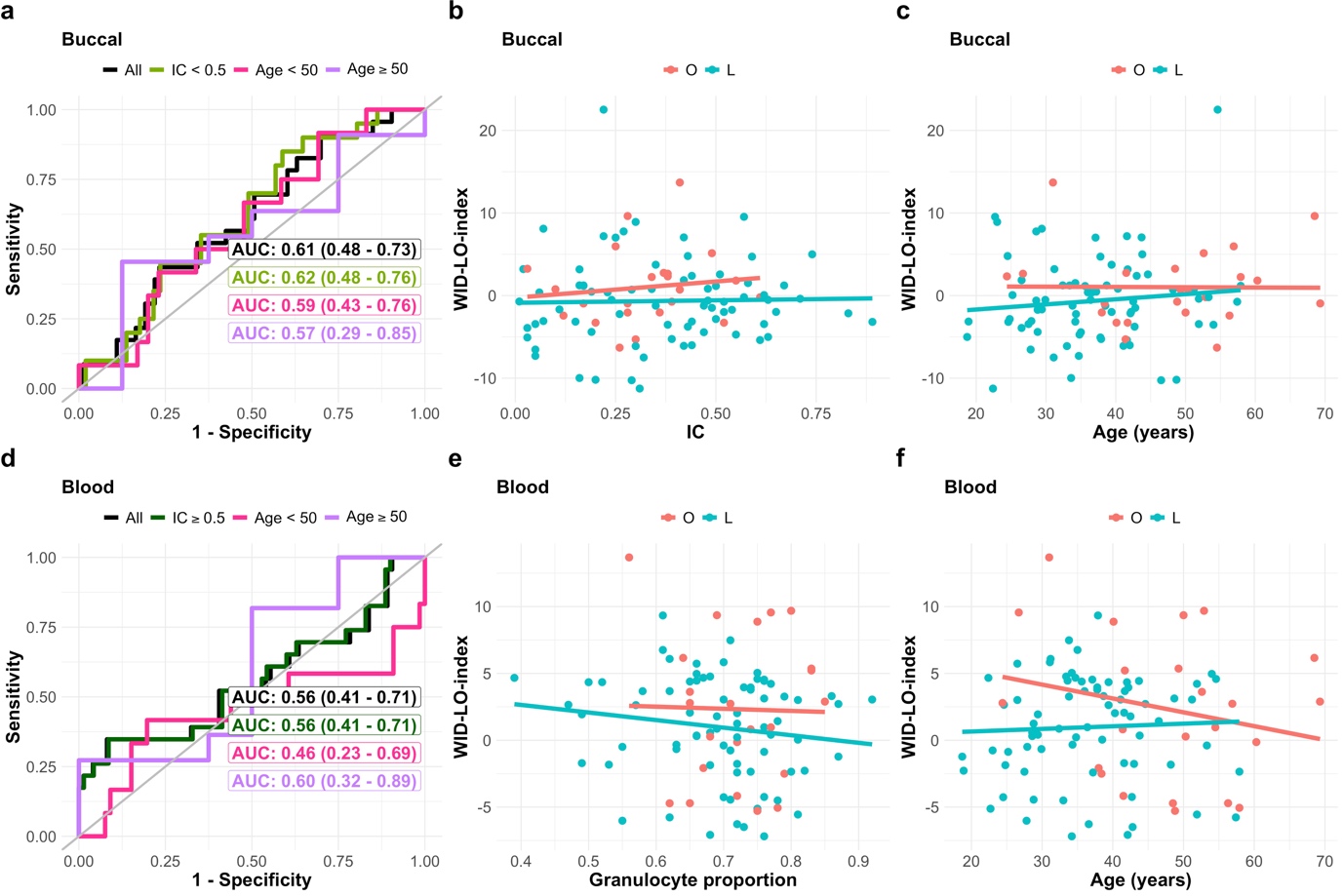


**Fig. S11 Performance of the non-linear classifier (NL WID-LO-index) in the training and validation sets. a, d,** ROC curves and AUC in the training set (**a**) and validation set (**d**). **b, e,** Non-linear index scatter plot with IC, in the training (**b**) and validation set (**e**). **c, f,** Non-linear index scatter plot with Age, in the training (**c**) and validation set (**f**). For the methods used to rank CpGs see Methods section. This best non-linear classifier was achieved with an input pool 60,000 CpGs and α=0.2. Only 1,162 features were selected, of which 104 are higher order terms with a group of CpGs of which 51 are not included in the linear terms. It was the 1,162 features signature that we applied in the validation set. IC = Immune Cell proportion.


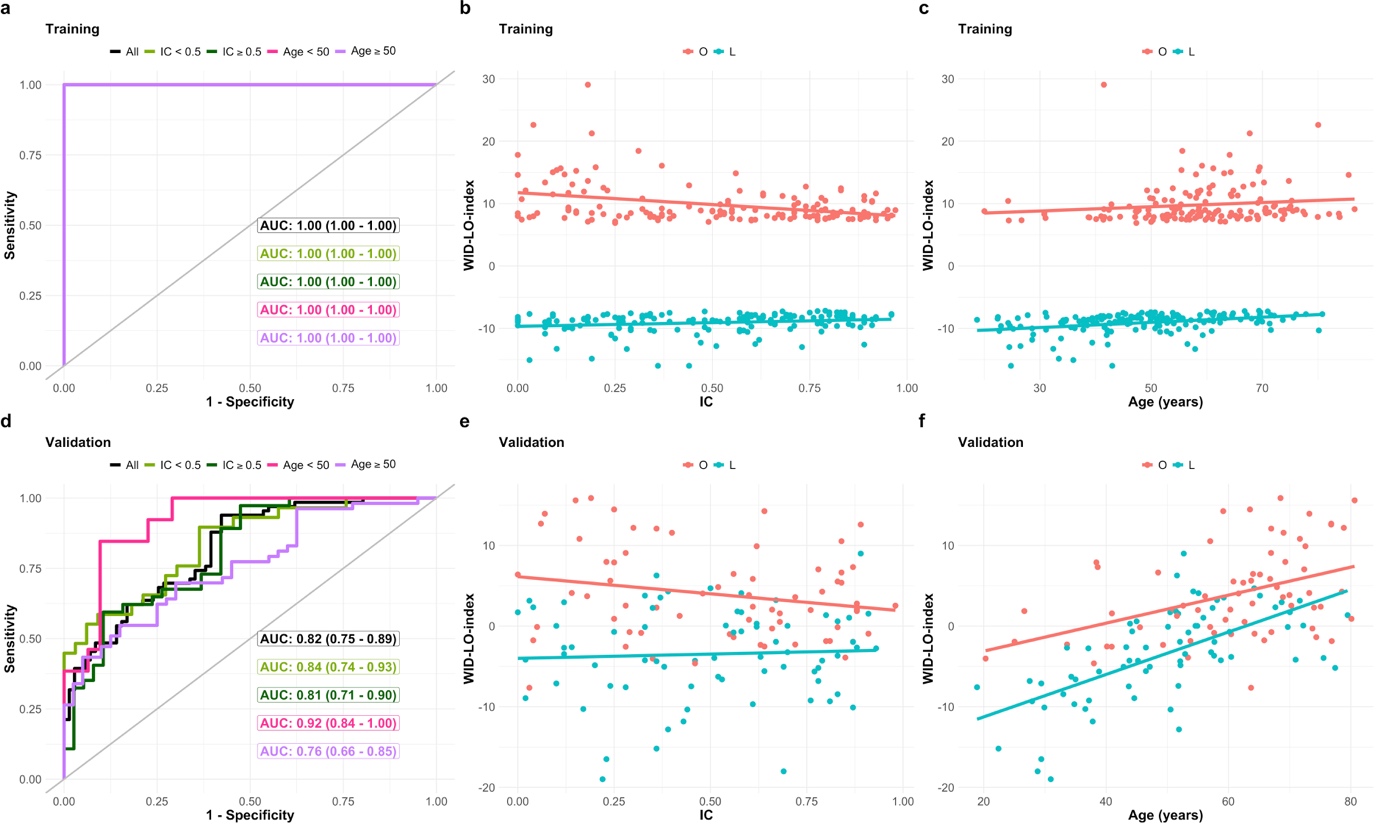


**Fig. S12 Association of top CpGs with gene region, CpG region, eFORGE analysis and gene set enrichment analysis, non-linear classifier (NL WID-LO-index). a, b,** Association of the top 60,000 CpGs (absolute numbers) ranked according to the geometric mean across 5 ranking algorithms, with CpG region (**a**) and gene region (**b**). **c,** Odds ratios for (**a**), where the p-values were calculated via the exact Fisher method (two-sided) against the proportions in the entirety of the EPIC array. **e,** eFORGE analysis of the top 1,000 CpGs in the 60,000 input CpG pool. **f,** Gene enrichment analysis for (**b**) via an empirical Bayes algorithm available in the *ChAMP* R package (version 2.14.0). For the methods used to rank CpGs see Methods section. This non-linear classifier was achieved with an input pool of 60,000 CpGs and α=0.2. Only 1,162 features were selected, of which 104 are higher order terms with a group of CpGs of which 51 are not included in the linear terms. It was the 1,162 features signature that we applied in the buccal and blood samples. IC = Immune Cell proportion. See Methods section.


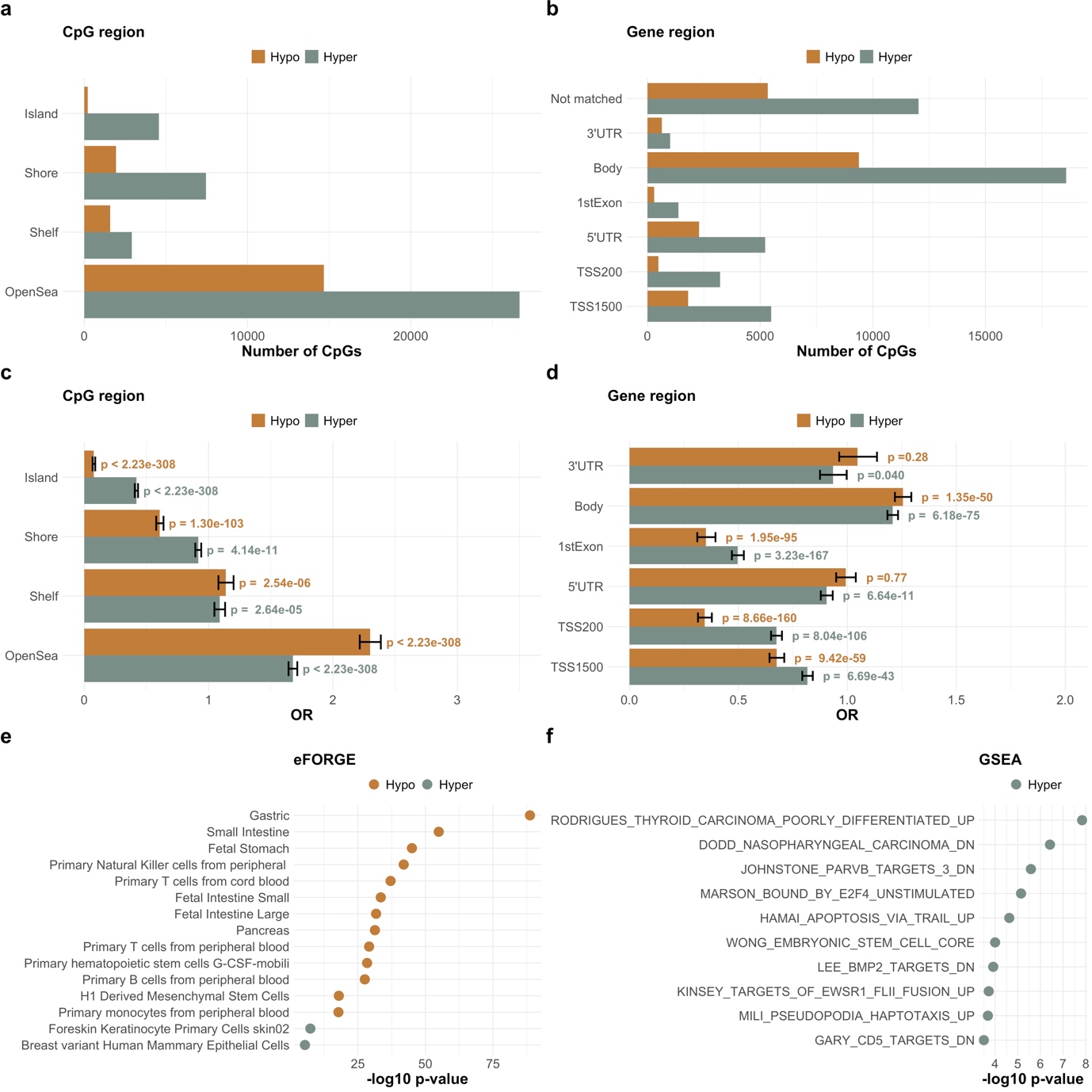


**Fig. S13 Performance of the non-linear classifier (NL WID-LO-index) in buccal and blood samples.** **a, d,** ROC curves and AUC in buccal (**a**) and blood (**d**). **b, e,** Non-linear index scatter plot with IC, in the buccal (**b**) and blood samples (**e**). **c, f,** Non-linear index scatter plot with Age, in buccal (**c**) and blood samples (**f**). For the methods used to rank CpGs see Methods section. This non-linear classifier was achieved with an input pool of 60,000 CpGs and α=0.2. Only 1,162 features were selected, of which 104 are higher order terms with a group of CpGs of which 51 are not included in the linear terms. It was the 1,162 features signature that we applied in the buccal and blood samples. IC = Immune Cell proportion.

**
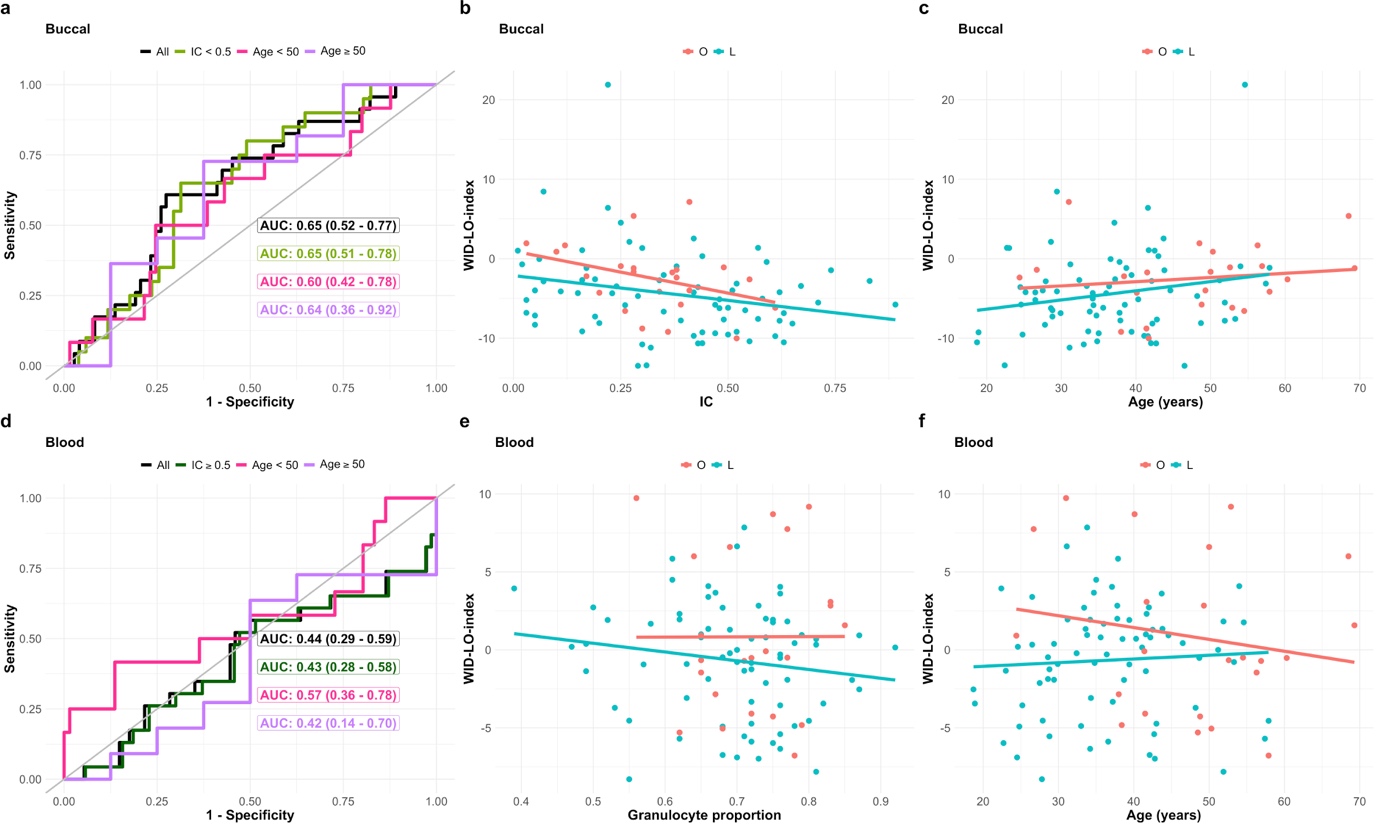
**

**Supplementary Tables**

**Table S1. Percentages and p-values for the association of each covariate and community-type determined from sequencing data for the training and validation sets.**  BMI=Body Mass Index (kg/m^2^). OCP=Oral Contraceptive Pill. HRT=Hormone Replacement Therapy. See Table S2 for number of missing values per covariate. p values were calculated under a logistic regression model with a bias reduction method (see Methods).

|  |  |  | **Training set** | | |  | **Validation set** | | |
| --- | --- | --- | --- | --- | --- | --- | --- | --- | --- |
|  | **Value** |  | **Community-type** | | **p value** |  | **Community-type** | | **p value** |
|  |  |  | **L** | **O** |  |  | **L** | **O** |  |
| **Ethnicity** | White |  | 151 (94%) | 139 (93%) | 0.86 |  | 65 (92%) | 56 (85%) | 0.23 |
|  | Non-White |  | 10 (6%) | 11 (7%) |  |  | 6 (8%) | 10 (15%) |  |
| **BMI** | ≤ 30 |  | 133 (83%) | 114 (76%) | 0.15 |  | 54 (76%) | 57 (86%) | 0.13 |
|  | > 30 |  | 28 (17%) | 36 (24%) |  |  | 17 (24%) | 9 (14%) |  |
| **Current smoking** | No |  | 141 (89%) | 132 (92%) | 0.39 |  | 57 (80%) | 57 (89%) | 0.17 |
|  | Yes |  | 18 (11%) | 12 (8%) |  |  | 14 (20%) | 7 (11%) |  |
| **Age at Menarche** | ≤ 12 |  | 134 (83%) | 142 (95%) | **0.0014** |  | 55 (79%) | 63 (95%) | **0.0030** |
|  | > 12 |  | 27 (17%) | 8 (5%) |  |  | 15 (21%) | 3 (5%) |  |
| **Duration of OCP use (years)** | < 5 |  | 88 (60%) | 104 (73%) | **0.020** |  | 39 (59%) | 49 (79%) | **0.015** |
|  | ≥ 5 |  | 58 (40%) | 38 (27%) |  |  | 27 (41%) | 13 (21%) |  |
| **Current OCP use** | No |  | 130 (89%) | 128 (91%) | 0.50 |  | 57 (86%) | 61 (98%) | **0.011** |
|  | Yes |  | 16 (11%) | 12 (9%) |  |  | 9 (14%) | 1 (2%) |  |
| **Ever pregnant** | No |  | 46 (29%) | 24 (16%) | **0.0079** |  | 17 (24%) | 7 (11%) | **0.046** |
|  | Yes |  | 114 (71%) | 125 (84%) |  |  | 54 (76%) | 58 (89%) |  |
| **Menopausal status** | Pre |  | 89 (55%) | 28 (19%) | **1.38E-11** |  | 31 (44%) | 12 (18%) | **0.0013** |
|  | Post |  | 72 (45%) | 122 (81%) |  |  | 40 (56%) | 54 (82%) |  |
| **HRT ever** | No |  | 138 (86%) | 121 (81%) | 0.23 |  | 59 (83%) | 50 (76%) | 0.29 |
|  | Yes |  | 22 (14%) | 28 (19%) |  |  | 12 (17%) | 16 (24%) |  |
| **Current combined hormone use** | No |  | 141 (91%) | 136 (95%) | 0.17 |  | 60 (88%) | 62 (98%) | **0.022** |
|  | Yes |  | 14 (9%) | 7 (5%) |  |  | 8 (12%) | 1 (2%) |  |
| **Duration of combined hormone use (years)** | < 5 |  | 99 (64%) | 105 (73%) | 0.11 |  | 42 (62%) | 51 (84%) | **0.0057** |
|  | ≥ 5 |  | 55 (36%) | 39 (27%) |  |  | 26 (38%) | 10 (16%) |  |

**Table S2. Number of missing values per covariate.** BMI=Body Mass Index (kg/m^2^). OCP=Oral Contraceptive Pill. HRT=Hormone Replacement Therapy.

| **Variable** | **Training set (n=311)** | **Validation set (n=137)** |
| --- | --- | --- |
| **Ethnicity** | 1 (0.32%) | 0 (0%) |
| **AGE (years)** | 0 (0%) | 0 (0%) |
| **BMI** | 0 (0%) | 0 (0%) |
| **Current smoking** | 8 (2.57%) | 2 (1.46%) |
| **Age at Menarche (years)** | 3 (0.96%) | 2 (1.46%) |
| **Duration of OCP use (years)** | 23 (7.40%) | 9 (6.57%) |
| **Current OCP use** | 25 (8.03%) | 9 (6.57%) |
| **Ever pregnant** | 2 (0.64%) | 1 (0.73%) |
| **Menopausal status** | 0 (0%) | 0 (0%) |
| **HRT ever** | 2 (0.64%) | 0 (0%) |
| **Current combined hormone use** | 13 (4.18%) | 6 (4.38%) |
| **Duration of combined hormone use (years)** | 13 (4.18%) | 8 (5.84%) |

**Table S3. Percentages and p-values for the association of each covariate and community-type determined from sequencing data for the training and validation sets, Age < 50 years.**  BMI=Body Mass index (kg/m^2^). OCP=Oral Contraceptive Pill. HRT=Hormone Replacement Therapy. p values were calculated under a logistic regression model with a bias reduction method (see Methods).

|  |  |  | **Training set** | | |  | **Validation set** | | |
| --- | --- | --- | --- | --- | --- | --- | --- | --- | --- |
|  | **Value** |  | **Community-type** | | **p value** |  | **Community-type** | | **p value** |
|  |  |  | **L** | **O** |  |  | **L** | **O** |  |
| **Ethnicity** | White |  | 81 (95%) | 29 (97%) | 0.93 |  | 27 (87%) | 11 (85%) | 0.74 |
|  | Non-White |  | 4 (5%) | 1 (3%) |  |  | 4 (13%) | 2 (15%) |  |
| **BMI** | ≤ 30 |  | 76 (89%) | 25 (83%) | 0.35 |  | 24 (77%) | 12 (92%) | 0.30 |
|  | > 30 |  | 9 (11%) | 5 (17%) |  |  | 7 (23%) | 1 (8%) |  |
| **Current smoking** | No |  | 74 (87%) | 26 (90%) | 0.81 |  | 27 (87%) | 12 (92%) | 0.75 |
|  | Yes |  | 11 (13%) | 3 (10%) |  |  | 4 (13%) | 1 (8%) |  |
| **Age at Menarche** | ≤ 12 |  | 62 (73%) | 26 (87%) | 0.14 |  | 16 (53%) | 12 (92%) | **0.013** |
|  | > 12 |  | 23 (27%) | 4 (13%) |  |  | 14 (47%) | 1 (8%) |  |
| **Duration of OCP use (years)** | < 5 |  | 39 (53%) | 18 (62%) | 0.40 |  | 16 (57%) | 9 (75%) | 0.31 |
|  | ≥ 5 |  | 35 (47%) | 11 (38%) |  |  | 12 (43%) | 3 (25%) |  |
| **Current OCP use** | No |  | 61 (82%) | 24 (86%) | 0.76 |  | 23 (82%) | 11 (92%) | 0.54 |
|  | Yes |  | 13 (18%) | 4 (14%) |  |  | 5 (18%) | 1 (8%) |  |
| **Ever pregnant** | No |  | 35 (41%) | 6 (20%) | **0.037** |  | 15 (48%) | 5 (42%) | 0.71 |
|  | Yes |  | 50 (59%) | 24 (80%) |  |  | 16 (52%) | 7 (58%) |  |
| **Menopausal status** | Pre |  | 79 (93%) | 22 (73%) | **0.0076** |  | 27 (87%) | 11 (85%) | 0.74 |
|  | Post |  | 6 (7%) | 8 (27%) |  |  | 4 (13%) | 2 (15%) |  |
| **HRT ever** | No |  | 80 (94%) | 29 (97%) | 0.59 |  | 28 (90%) | 13 (100%) | 0.38 |
|  | Yes |  | 5 (6%) | 1 (3%) |  |  | 3 (10%) | 0 (0%) |  |
| **Current combined hormone use** | No |  | 71 (86%) | 25 (86%) | 0.93 |  | 25 (83%) | 11 (92%) | 0.60 |
|  | Yes |  | 12 (14%) | 4 (14%) |  |  | 5 (17%) | 1 (8%) |  |
| **Duration of combined hormone use (years)** | < 5 |  | 49 (59%) | 18 (60%) | 0.93 |  | 19 (63%) | 9 (75%) | 0.51 |
|  | ≥ 5 |  | 34 (41%) | 12 (40%) |  |  | 11 (37%) | 3 (25%) |  |

**Table S4. Percentages and p-values for the association of each covariate and community-type determined from sequencing data for the training and validation sets, Age ≥ 50 years.**  BMI=Body Mass Index (kg/m^2^). OCP=Oral Contraceptive Pill. HRT=Hormone Replacement Therapy. p values were calculated under a logistic regression model with a bias reduction method (see Methods).

|  |  |  | **Training set** | | |  | **Validation set** | | |
| --- | --- | --- | --- | --- | --- | --- | --- | --- | --- |
|  | **Value** |  | **Community-type** | | **p value** |  | **Community-type** | | **p value** |
|  |  |  | **L** | **O** |  |  | **L** | **O** |  |
| **Ethnicity** | White |  | 70 (92%) | 110 (92%) | 0.90 |  | 38 (95%) | 45 (85%) | 0.13 |
|  | Non-White |  | 6 (8%) | 9 (8%) |  |  | 2 (5%) | 8 (15%) |  |
| **BMI** | ≤ 30 |  | 57 (75%) | 89 (74%) | 0.91 |  | 30 (75%) | 45 (85%) | 0.24 |
|  | > 30 |  | 19 (25%) | 31 (26%) |  |  | 10 (25%) | 8 (15%) |  |
| **Current smoking** | No |  | 67 (91%) | 106 (92%) | 0.67 |  | 30 (75%) | 45 (88%) | 0.11 |
|  | Yes |  | 7 (9%) | 9 (8%) |  |  | 10 (25%) | 6 (12%) |  |
| **Age at Menarche** | ≤ 12 |  | 71 (95%) | 114 (97%) | 0.49 |  | 38 (97%) | 51 (96%) | 0.83 |
|  | > 12 |  | 4 (5%) | 4 (3%) |  |  | 1 (3%) | 2 (4%) |  |
| **Duration of OCP use (years)** | < 5 |  | 49 (68%) | 86 (76%) | 0.23 |  | 23 (61%) | 40 (80%) | **0.047** |
|  | ≥ 5 |  | 23 (32%) | 27 (24%) |  |  | 15 (39%) | 10 (20%) |  |
| **Current OCP use** | No |  | 69 (96%) | 104 (93%) | 0.45 |  | 34 (89%) | 50 (100%) | **0.024** |
|  | Yes |  | 3 (4%) | 8 (7%) |  |  | 4 (11%) | 0 (0%) |  |
| **Ever pregnant** | No |  | 11 (15%) | 18 (15%) | 0.96 |  | 2 (5%) | 2 (4%) | 0.75 |
|  | Yes |  | 64 (85%) | 101 (85%) |  |  | 38 (95%) | 51 (96%) |  |
| **Menopausal status** | Pre |  | 10 (13%) | 6 (5%) | **0.046** |  | 4 (10%) | 1 (2%) | 0.10 |
|  | Post |  | 66 (87%) | 114 (95%) |  |  | 36 (90%) | 52 (98%) |  |
| **HRT ever** | No |  | 58 (77%) | 92 (77%) | 0.98 |  | 31 (78%) | 37 (70%) | 0.42 |
|  | Yes |  | 17 (23%) | 27 (23%) |  |  | 9 (22%) | 16 (30%) |  |
| **Current combined hormone use** | No |  | 70 (97%) | 111 (97%) | 0.88 |  | 35 (92%) | 51 (100%) | **0.057** |
|  | Yes |  | 2 (3%) | 3 (3%) |  |  | 3 (8%) | 0 (0%) |  |
| **Duration of combined hormone use (years)** | < 5 |  | 50 (70%) | 87 (76%) | 0.37 |  | 23 (61%) | 42 (86%) | **0.0080** |
|  | ≥ 5 |  | 21 (30%) | 27 (24%) |  |  | 15 (39%) | 7 (14%) |  |

**Table S5 Odds-ratios and p-values for the association between each covariate and the WID-LO-index, Age < 50 years.** BMI=Body Mass Index (kg/m^2^). OCP=Oral Contraceptive Pill. HRT=Hormone Replacement Therapy. Odds ratio, confidence intervals and p values were calculated under a logistic regression model with a bias reduction method (see Methods). This linear classifier involves 819 CpGs. See also Fig. S6 and Table S7.

|  |  |  | **Training set** | | |  | **Validation set** | |
| --- | --- | --- | --- | --- | --- | --- | --- | --- |
|  | **Value** |  | **OR (95% CI)** | **p value** | |  | **OR (95% CI)** | **p value** |
| **Ethnicity** | White vs Non-White |  | 1.00 (0.89 – 1.09) | 0.97 |  | | 1.03 (0.90 – 1.20) | 0.67 |
| **BMI** | ≤ 30 vs >30 |  | 1.02 (0.96 – 1.08) | 0.46 |  | | 0.95 (0.84 - 1.07) | 0.40 |
| **Current smoking** | No vs Yes |  | 0.99 (0.93 - 1.05) | 0.86 |  | | 1.01 (0.88 - 1.19) | 0.85 |
| **Age at Menarche** | ≤ 12 vs >12 |  | 0.93 (0.87 – 0.99) | **0.020** |  | | 0.86 (0.74 - 0.96) | **0.0064** |
| **Duration of OCP use (years)** | ≤ 5 vs >5 |  | 0.98 (0.94 - 1.02) | 0.27 |  | | 0.91 (0.80 – 1.02) | 0.12 |
| **Current OCP use** | No vs Yes |  | 0.98 (0.92 - 1.04) | 0.51 |  | | 0.95 (0.81 - 1.10) | 0.48 |
| **Ever pregnant** | No vs Yes |  | 1.06 (1.01 - 1.12) | **0.020** |  | | 1.07 (0.97 – 1.19) | 0.18 |
| **Menopausal status** | Pre vs Post |  | 1.08 (1.02 - 1.14) | **0.0071** |  | | 1.15 (0.99 – 1.38) | 0.080 |
| **HRT ever** | No vs Yes |  | 0.99 (0.89 - 1.07) | 0.87 |  | | 1.02 (0.85 – 1.24) | 0.86 |
| **Current combined hormone use** | No vs Yes |  | 0.99 (0.93 - 1.05) | 0.79 |  | | 0.95 (0.82 – 1.11) | 0.54 |
| **Duration of combined hormone use (years)** | ≤ 5 vs >5 |  | 0.99 (0.95 - 1.03) | 0.75 |  | | 0.96 (0.85 - 1.07) | 0.48 |

**Table S6 Odds-ratios and p-values for the association between each covariate (response) and the WID-LO-index, Age ≥ 50 years.** BMI=Body Mass Index (kg/m^2^). OCP=Oral Contraceptive Pill. HRT=Hormone Replacement Therapy. Odds ratio, confidence intervals and p values were calculated under a logistic regression model with a bias reduction method (see Methods). This linear classifier involves 819 CpGs. See also Fig. S6 and Table S7.

|  |  |  | **Training set** | |  | **Validation set** | |
| --- | --- | --- | --- | --- | --- | --- | --- |
|  | **Value** |  | **OR (95% CI)** | **p value** |  | **OR (95% CI)** | **p value** |
| **Ethnicity** | White vs Non-White |  | 0.99 (0.94 - 1.04) | 0.63 |  | 1.08 (0.98 - 1.19) | 0.12 |
| **BMI** | ≤ 30 vs >30 |  | 1.01 (0.98 - 1.04) | 0.69 |  | 0.90 (0.81 - 0.98) | **0.019** |
| **Current smoking** | No vs Yes |  | 0.99 (0.94 - 1.04) | 0.66 |  | 0.93 (0.85 - 1.02) | 0.12 |
| **Age at Menarche** | ≤ 12 vs >12 |  | 0.98 (0.91 - 1.05) | 0.50 |  | 1.10 (0.94 – 1.30) | 0.22 |
| **Duration of OCP use (years)** | ≤ 5 vs >5 |  | 0.99 (0.96 - 1.02) | 0.23 |  | 0.93 (0.86 - 1.00) | 0.064 |
| **Current OCP use** | No vs Yes |  | 1.02 (0.96 - 1.02) | 0.45 |  | 0.80 (0.64 – 0.97) | **0.018** |
| **Ever pregnant** | No vs Yes |  | 1.01 (0.97 - 1.05) | 0.64 |  | 1.12 (0.95 - 1.35) | 0.21 |
| **Menopausal status** | Pre vs Post |  | 1.06 (1.01 - 1.12) | **0.030** |  | 1.07 (0.93 - 1.26) | 0.38 |
| **HRT ever** | No vs Yes |  | 1.00 (0.96 - 1.03) | 0.84 |  | 1.03 (0.96 - 1.11) | 0.38 |
| **Current combined hormone use** | No vs Yes |  | 0.98 (0.90 - 1.07) | 0.69 |  | 0.87 (0.70 - 1.05) | 0.16 |
| **Duration of combined hormone use (years)** | ≤ 5 vs >5 |  | 0.99 (0.96 - 1.02) | 0.57 |  | 0.94 (0.87 - 1.01) | 0.12 |

**Table S7. Elastic net WID-LO-index in the training set**. Δβ corresponds to the difference in the estimated y-intercept of two independent linear fits β vs Immune Cell fraction, i.e. one to the subjects of community-type L and another to the subjects of community-type O. The p values were calculated under a logistic regression model adjusted for Age and Immune Cell fraction. The q values were determined with the *qvalue* package (version 2.16.0) in R by calculating the False Discovery Rate. In this table, the CpGs were ranked according to the q values. **‘**WID-LO-index coef’ corresponds to the CpG’s coefficients in the classifier developed with the *glmnet* R package (version version 2.0.18). The best classifier in the training set was the Elastic Net, with an input pool of 50,000 CpGs, hyperparameter α=0.3, resulting in 819 non-zero components (selected CpGs) which we present here. This was the linear signature applied in the validation set and in the buccal and blood samples. See also Fig. S6.

| **IlumnID** | **UCSC_RefGene_Name** | **UCSC_RefGene_Region** | **CpG**  **region** | **Mean β**  **type L** | **Mean β**  **type O** | **Δβ** | **p value** | **q value** | **WID-LO-index coef** |
| --- | --- | --- | --- | --- | --- | --- | --- | --- | --- |
| cg06209197 | PEF1 | Body | N_Shelf | 6.16E-01 | 7.51E-01 | 2.11E-01 | 2.72E-11 | 8.04E-06 | 9.32E-02 |
| cg10160988 | SCART1 | Body | S_Shore | 8.63E-01 | 9.08E-01 | 9.28E-02 | 5.92E-10 | 1.70E-05 | 1.69E+00 |
| cg08768753 |  |  | OpenSea | 8.50E-01 | 8.96E-01 | 9.16E-02 | 2.77E-09 | 2.88E-05 | 1.97E+00 |
| cg13465252 |  |  | OpenSea | 8.92E-01 | 9.32E-01 | 9.90E-02 | 7.22E-09 | 3.63E-05 | 3.85E-01 |
| cg20068402 |  |  | OpenSea | 5.23E-01 | 6.41E-01 | 2.24E-01 | 7.86E-09 | 3.63E-05 | 7.56E-01 |
| cg05080979 | BTBD11 | Body | OpenSea | 1.59E-01 | 2.40E-01 | 2.11E-01 | 1.49E-08 | 4.86E-05 | 7.29E-01 |
| cg12065406 | QSOX1;FLJ23867 | Body;TSS1500 | OpenSea | 9.77E-01 | 9.83E-01 | 9.55E-03 | 4.67E-08 | 7.01E-05 | 1.38E+01 |
| cg17751982 |  |  | OpenSea | 2.85E-01 | 2.22E-01 | -6.98E-02 | 6.76E-08 | 7.98E-05 | -2.28E+00 |
| cg21692534 |  |  | N_Shelf | 7.33E-01 | 6.47E-01 | -1.54E-01 | 7.70E-08 | 8.45E-05 | -3.86E-01 |
| cg25395997 | OXER1 | TSS1500 | OpenSea | 9.70E-01 | 9.77E-01 | 1.00E-02 | 7.87E-08 | 8.54E-05 | 2.46E-01 |
| cg10891673 |  |  | OpenSea | 1.05E-01 | 1.41E-01 | 3.44E-02 | 8.66E-08 | 8.77E-05 | 4.16E-02 |
| cg19625573 | SULT4A1 | Body | OpenSea | 9.10E-01 | 9.26E-01 | 1.71E-02 | 9.18E-08 | 8.77E-05 | 3.42E+00 |
| cg06649805 | RGS7 | Body | OpenSea | 6.53E-01 | 7.82E-01 | 1.74E-01 | 9.47E-08 | 8.87E-05 | 2.07E+00 |
| cg24402534 | PTK2B | ExonBnd;Body | OpenSea | 9.18E-01 | 9.37E-01 | 2.55E-02 | 1.05E-07 | 8.91E-05 | 7.28E-01 |
| cg02162745 |  |  | OpenSea | 7.09E-01 | 8.09E-01 | 2.10E-01 | 1.05E-07 | 8.91E-05 | 1.37E-01 |
| cg04424643 | ESRRB | 5'UTR | OpenSea | 5.86E-01 | 7.21E-01 | 1.84E-01 | 1.26E-07 | 9.47E-05 | 2.84E+00 |
| cg12037784 | SH3BP4 | 3'UTR | OpenSea | 9.35E-01 | 9.47E-01 | 1.18E-02 | 1.29E-07 | 9.53E-05 | 1.22E+01 |
| cg26502105 |  |  | OpenSea | 7.24E-01 | 7.69E-01 | 8.89E-02 | 1.30E-07 | 9.53E-05 | 4.56E-02 |
| cg13572584 | BIN1 | Body | OpenSea | 9.65E-01 | 9.76E-01 | 1.12E-02 | 1.61E-07 | 1.02E-04 | 1.64E+01 |
| cg14700035 | PFKP | Body | OpenSea | 9.00E-01 | 9.26E-01 | 5.38E-02 | 1.83E-07 | 1.08E-04 | 1.82E-01 |
| cg12939918 | TSNARE1 | Body | S_Shore | 9.17E-01 | 9.32E-01 | 1.56E-02 | 2.22E-07 | 1.14E-04 | 5.66E+00 |
| cg20901711 |  |  | OpenSea | 9.34E-01 | 9.49E-01 | 1.33E-02 | 2.73E-07 | 1.23E-04 | 1.12E+00 |
| cg08068630 |  |  | OpenSea | 8.44E-01 | 9.70E-01 | 1.66E-01 | 2.99E-07 | 1.24E-04 | 4.39E+00 |
| cg15483225 | NUBP1 | Body | OpenSea | 8.50E-01 | 7.80E-01 | -1.63E-01 | 4.13E-07 | 1.40E-04 | -6.63E-01 |
| cg21902975 |  |  | OpenSea | 8.86E-01 | 7.98E-01 | -2.80E-01 | 4.45E-07 | 1.44E-04 | -6.05E-01 |
| cg20664654 | AVPR2 | TSS1500;Body | OpenSea | 6.91E-01 | 6.43E-01 | -5.11E-02 | 4.86E-07 | 1.47E-04 | -2.64E+00 |
| cg26078475 |  |  | OpenSea | 3.53E-01 | 4.31E-01 | 1.83E-01 | 5.94E-07 | 1.56E-04 | 1.12E+00 |
| cg01015210 |  |  | OpenSea | 2.91E-01 | 2.34E-01 | -1.21E-01 | 6.05E-07 | 1.57E-04 | -1.40E+00 |
| cg24205662 | SLC22A23 | Body | OpenSea | 9.62E-01 | 9.71E-01 | 7.21E-03 | 6.75E-07 | 1.65E-04 | 3.59E-03 |
| cg15360480 | SLC22A7 | Body | OpenSea | 9.12E-01 | 9.31E-01 | 2.46E-02 | 6.94E-07 | 1.67E-04 | 2.77E-01 |
| cg24587280 |  |  | OpenSea | 7.91E-02 | 9.50E-02 | 5.23E-02 | 7.63E-07 | 1.73E-04 | 2.16E+00 |
| cg20722210 |  |  | N_Shore | 4.53E-01 | 5.00E-01 | 1.01E-01 | 9.42E-07 | 1.86E-04 | 1.51E-01 |
| cg14204372 | LOC339862 | Body | OpenSea | 9.13E-01 | 9.31E-01 | 3.02E-02 | 1.13E-06 | 2.01E-04 | 3.43E+00 |
| cg15434778 | NRXN3 | Body | OpenSea | 8.70E-01 | 8.44E-01 | -3.45E-02 | 1.18E-06 | 2.04E-04 | -3.71E+00 |
| cg01535205 |  |  | OpenSea | 8.52E-01 | 8.88E-01 | 6.77E-02 | 1.36E-06 | 2.16E-04 | 9.58E-01 |
| cg10412231 |  |  | Island | 9.69E-01 | 9.77E-01 | 7.34E-03 | 1.44E-06 | 2.21E-04 | 2.01E+01 |
| cg26647238 | CRADD;LOC101928731 | Body | OpenSea | 5.71E-01 | 4.78E-01 | -1.99E-01 | 1.66E-06 | 2.34E-04 | -1.65E-01 |
| cg27412310 | GDPD5 | Body | OpenSea | 9.42E-01 | 9.57E-01 | 2.56E-02 | 1.69E-06 | 2.36E-04 | 4.66E+00 |
| cg21061317 |  |  | OpenSea | 4.88E-01 | 6.01E-01 | 2.34E-01 | 1.69E-06 | 2.36E-04 | 2.60E-02 |
| cg12955870 | ABCB11 | TSS200 | OpenSea | 7.46E-01 | 7.82E-01 | 5.23E-02 | 1.89E-06 | 2.49E-04 | 6.00E-01 |
| cg08716255 |  |  | OpenSea | 5.91E-01 | 4.54E-01 | -3.08E-01 | 1.95E-06 | 2.52E-04 | -1.48E-01 |
| cg20991141 | RGL4;GUSBP11 | Body | OpenSea | 9.73E-01 | 9.81E-01 | 1.16E-02 | 2.01E-06 | 2.55E-04 | 5.62E-03 |
| cg24935031 |  |  | OpenSea | 9.26E-01 | 9.52E-01 | 3.25E-02 | 2.10E-06 | 2.61E-04 | 6.63E+00 |
| cg14600855 | CLIC5 | Body | OpenSea | 9.60E-01 | 9.21E-01 | -8.20E-02 | 2.11E-06 | 2.61E-04 | -1.27E-01 |
| cg11065065 |  |  | OpenSea | 3.98E-01 | 4.34E-01 | 2.11E-02 | 2.27E-06 | 2.71E-04 | 7.88E-01 |
| cg04560630 | EPS8L2 | Body | Island | 2.14E-01 | 2.75E-01 | 1.06E-01 | 2.32E-06 | 2.74E-04 | 7.32E-01 |
| cg22667358 | PFKP | Body | OpenSea | 6.79E-01 | 7.23E-01 | 6.04E-02 | 2.39E-06 | 2.78E-04 | 3.08E-01 |
| cg15656004 |  |  | OpenSea | 5.26E-01 | 5.50E-01 | 8.15E-03 | 2.52E-06 | 2.84E-04 | 2.06E+00 |
| cg00602326 | RNASEN | Body | OpenSea | 7.84E-01 | 8.38E-01 | 8.62E-02 | 2.71E-06 | 2.94E-04 | 1.28E+00 |
| cg07270545 | UNC13B | Body | OpenSea | 8.63E-01 | 8.22E-01 | -3.36E-02 | 2.79E-06 | 2.98E-04 | -4.56E-01 |
| cg02193580 | FHL1 | TSS200;Body;5'UTR;TSS1500 | OpenSea | 4.44E-01 | 4.67E-01 | 2.78E-02 | 2.81E-06 | 2.99E-04 | 7.67E-01 |
| cg19580156 | IQCA1 | Body | OpenSea | 9.61E-01 | 9.69E-01 | 3.27E-03 | 3.31E-06 | 3.25E-04 | 1.76E+00 |
| cg18842178 |  |  | OpenSea | 6.53E-01 | 7.23E-01 | 1.17E-01 | 3.46E-06 | 3.32E-04 | 1.27E-01 |
| cg13178170 | TGFBR3 | Body | OpenSea | 5.94E-01 | 6.66E-01 | 1.43E-01 | 3.60E-06 | 3.39E-04 | 1.26E-01 |
| cg22238104 | HIVEP3 | Body | OpenSea | 9.30E-01 | 9.65E-01 | 4.43E-02 | 3.62E-06 | 3.40E-04 | 1.43E+01 |
| cg12901589 | PSAPL1;SORCS2 | 1stExon;Body | OpenSea | 9.18E-01 | 9.38E-01 | 1.58E-02 | 3.71E-06 | 3.44E-04 | 1.44E-01 |
| cg20948431 | C4orf10;NOP14 | Body | OpenSea | 6.55E-01 | 7.18E-01 | 9.08E-02 | 3.73E-06 | 3.45E-04 | 1.53E-01 |
| cg21911868 | ESPNL | Body | N_Shelf | 9.76E-01 | 9.82E-01 | 6.60E-03 | 3.77E-06 | 3.46E-04 | 6.15E+00 |
| cg13214286 | PPP6R1 | 5'UTR | OpenSea | 4.12E-01 | 3.37E-01 | -1.53E-01 | 4.19E-06 | 3.63E-04 | -9.73E-01 |
| cg03750993 | ACSS1 | TSS1500 | S_Shore | 8.77E-01 | 9.02E-01 | 4.41E-02 | 4.33E-06 | 3.69E-04 | -8.45E-01 |
| cg18472448 |  |  | OpenSea | 1.34E-01 | 9.11E-02 | -9.42E-02 | 4.34E-06 | 3.69E-04 | -2.91E-01 |
| cg12749719 | ARHGAP1 | Body | OpenSea | 9.41E-01 | 9.55E-01 | 1.78E-02 | 5.00E-06 | 3.97E-04 | 4.02E+00 |
| cg14779065 | DPP4 | TSS1500 | S_Shore | 9.37E-01 | 9.68E-01 | 6.86E-02 | 5.27E-06 | 4.08E-04 | 3.67E+00 |
| cg19983527 | PDE6C | Body | OpenSea | 9.35E-01 | 9.16E-01 | -2.91E-02 | 5.52E-06 | 4.15E-04 | 4.41E-01 |
| cg15929513 | TMCC2 | Body | Island | 9.01E-01 | 9.27E-01 | 1.88E-02 | 6.10E-06 | 4.38E-04 | 2.29E+00 |
| cg22511153 | LOC729987 | TSS1500 | OpenSea | 8.68E-01 | 8.39E-01 | -1.60E-02 | 6.19E-06 | 4.41E-04 | -9.02E-02 |
| cg19735563 | ITIH6 | Body | OpenSea | 9.32E-01 | 9.51E-01 | 1.72E-02 | 6.24E-06 | 4.43E-04 | 2.81E-01 |
| cg02344373 | POLE | Body | OpenSea | 8.45E-01 | 8.59E-01 | 1.65E-02 | 6.33E-06 | 4.47E-04 | 2.18E+00 |
| cg11772416 |  |  | OpenSea | 5.51E-01 | 5.92E-01 | 6.05E-02 | 6.45E-06 | 4.51E-04 | 1.80E+00 |
| cg03033953 | TSHZ2 | Body | OpenSea | 4.63E-02 | 5.96E-02 | 3.52E-02 | 6.74E-06 | 4.62E-04 | 1.47E+00 |
| cg09740350 |  |  | OpenSea | 7.03E-01 | 7.34E-01 | -3.05E-03 | 6.78E-06 | 4.63E-04 | 2.06E+00 |
| cg13330574 | SLC12A8 | Body | OpenSea | 9.19E-01 | 9.34E-01 | 1.30E-02 | 7.18E-06 | 4.77E-04 | 6.24E-01 |
| cg11749817 | ARFGEF2 | Body | OpenSea | 1.23E-01 | 2.14E-01 | 9.95E-02 | 7.35E-06 | 4.83E-04 | 7.60E-01 |
| cg22664993 | HEPACAM | Body | S_Shelf | 9.80E-01 | 9.86E-01 | 8.56E-03 | 7.57E-06 | 4.89E-04 | 7.54E+00 |
| cg19373635 |  |  | OpenSea | 8.17E-02 | 1.07E-01 | 2.74E-02 | 7.68E-06 | 4.93E-04 | 1.53E+00 |
| cg01959919 |  |  | OpenSea | 9.22E-01 | 9.52E-01 | 6.06E-02 | 7.76E-06 | 4.95E-04 | 4.59E+00 |
| cg11461359 | EYA2 | 5'UTR | OpenSea | 8.98E-01 | 8.76E-01 | -9.81E-03 | 7.99E-06 | 5.03E-04 | -1.96E-01 |
| cg12111783 | BCOR | 5'UTR | Island | 9.90E-02 | 1.39E-01 | 5.81E-02 | 8.57E-06 | 5.23E-04 | 1.47E-01 |
| cg11787522 | STRA6 | 1stExon;5'UTR | OpenSea | 3.14E-01 | 2.84E-01 | -6.12E-02 | 9.00E-06 | 5.38E-04 | -3.23E+00 |
| cg21490179 |  |  | Island | 1.78E-02 | 2.10E-02 | 3.24E-03 | 9.70E-06 | 5.60E-04 | 9.26E+00 |
| cg16170924 |  |  | OpenSea | 8.58E-01 | 9.05E-01 | 9.31E-02 | 1.06E-05 | 5.88E-04 | -2.52E+00 |
| cg25914941 | MATN2 | Body | OpenSea | 9.78E-01 | 9.83E-01 | 3.78E-03 | 1.09E-05 | 5.95E-04 | 4.22E+00 |
| cg16572050 |  |  | OpenSea | 9.31E-01 | 9.47E-01 | 2.87E-02 | 1.09E-05 | 5.96E-04 | -1.84E+00 |
| cg00315525 | PREX1 | Body | OpenSea | 6.74E-01 | 7.15E-01 | 6.76E-02 | 1.12E-05 | 6.05E-04 | -6.49E-01 |
| cg20703684 | MAGI1 | Body | OpenSea | 2.37E-01 | 2.62E-01 | 2.83E-02 | 1.15E-05 | 6.12E-04 | 1.72E+00 |
| cg06150764 |  |  | Island | 3.12E-02 | 4.12E-02 | 4.60E-03 | 1.16E-05 | 6.16E-04 | 9.83E+00 |
| cg11728201 | AKAP12 | Body | OpenSea | 9.51E-01 | 9.62E-01 | 1.17E-02 | 1.18E-05 | 6.20E-04 | -3.08E+00 |
| cg23371476 |  |  | OpenSea | 6.53E-01 | 7.03E-01 | 4.87E-02 | 1.21E-05 | 6.30E-04 | 1.07E+00 |
| cg20806130 | ATAD2B | TSS1500 | S_Shore | 1.96E-02 | 2.30E-02 | 2.78E-03 | 1.23E-05 | 6.36E-04 | 2.00E+01 |
| cg02699703 | COQ9 | Body | N_Shelf | 9.62E-01 | 9.71E-01 | 7.89E-03 | 1.25E-05 | 6.41E-04 | 6.17E+00 |
| cg06720632 | NUMA1 | Body;5'UTR | OpenSea | 2.55E-01 | 3.47E-01 | 1.96E-01 | 1.27E-05 | 6.49E-04 | 5.85E-01 |
| cg21421846 |  |  | OpenSea | 9.47E-01 | 9.61E-01 | 1.87E-02 | 1.32E-05 | 6.61E-04 | -4.00E+00 |
| cg23248654 | DRAM1 | Body | OpenSea | 1.00E-01 | 1.30E-01 | 3.76E-02 | 1.36E-05 | 6.75E-04 | -4.90E-01 |
| cg02999834 | GABBR2 | Body | OpenSea | 9.08E-01 | 9.28E-01 | 1.56E-02 | 1.38E-05 | 6.81E-04 | 1.46E-01 |
| cg11417165 |  |  | OpenSea | 9.33E-01 | 9.54E-01 | 5.73E-02 | 1.40E-05 | 6.86E-04 | 2.23E-01 |
| cg19907776 |  |  | OpenSea | 1.18E-01 | 1.61E-01 | 3.14E-02 | 1.40E-05 | 6.87E-04 | 2.02E+00 |
| cg12390003 | IGSF8 | TSS1500 | S_Shore | 2.19E-01 | 2.67E-01 | 3.29E-02 | 1.44E-05 | 6.96E-04 | 2.50E+00 |
| cg19750120 |  |  | OpenSea | 9.27E-01 | 9.47E-01 | 3.49E-02 | 1.52E-05 | 7.21E-04 | 9.51E-01 |
| cg15972516 | TRRAP | Body | OpenSea | 9.86E-01 | 9.89E-01 | 1.40E-03 | 1.54E-05 | 7.26E-04 | 1.34E+01 |
| cg10903916 | ASAP2 | Body | N_Shore | 9.79E-01 | 9.74E-01 | -7.09E-03 | 1.56E-05 | 7.32E-04 | -3.55E+00 |
| cg13984005 | ALDH1B1 | 5'UTR | S_Shelf | 9.26E-01 | 9.38E-01 | 1.41E-02 | 1.65E-05 | 7.57E-04 | -5.78E-02 |
| cg18573877 | MSI2 | TSS1500;TSS200 | Island | 3.62E-02 | 4.26E-02 | 5.71E-03 | 1.74E-05 | 7.77E-04 | 4.10E+00 |
| cg16172001 |  |  | S_Shelf | 8.66E-01 | 8.86E-01 | 3.77E-02 | 1.75E-05 | 7.80E-04 | -2.62E-01 |
| cg04801326 | FBXL19 | ExonBnd;Body | OpenSea | 3.82E-02 | 3.28E-02 | -6.38E-03 | 1.76E-05 | 7.83E-04 | -2.25E+01 |
| cg07232789 | PLXND1 | Body | OpenSea | 8.44E-01 | 9.03E-01 | 1.18E-01 | 1.77E-05 | 7.85E-04 | 3.36E-01 |
| cg07608728 | CCDC3 | Body | OpenSea | 9.64E-01 | 9.71E-01 | 5.62E-03 | 1.82E-05 | 7.99E-04 | 6.55E+00 |
| cg13545236 | PITPNM3 | Body | OpenSea | 7.80E-01 | 8.33E-01 | 1.13E-01 | 1.86E-05 | 8.07E-04 | 1.45E+00 |
| cg07975242 | SCLY;UBE2F-SCLY | Body | OpenSea | 9.73E-01 | 9.79E-01 | 7.98E-03 | 1.87E-05 | 8.11E-04 | 3.52E+00 |
| cg15296871 |  |  | OpenSea | 9.34E-01 | 9.49E-01 | 1.83E-02 | 1.87E-05 | 8.11E-04 | 6.11E-01 |
| cg21940081 | IMP4 | Body | S_Shelf | 9.68E-01 | 9.57E-01 | -1.41E-02 | 1.91E-05 | 8.22E-04 | -4.70E+00 |
| cg13916255 | CALCR | 5'UTR;TSS1500 | OpenSea | 7.47E-01 | 7.89E-01 | 8.59E-02 | 2.08E-05 | 8.63E-04 | 3.88E-01 |
| cg09179713 | FLI1 | 5'UTR;Body | OpenSea | 9.79E-01 | 9.87E-01 | 8.97E-03 | 2.19E-05 | 8.90E-04 | 3.75E+00 |
| cg26437845 | C1orf187 | Body | N_Shore | 2.81E-01 | 2.46E-01 | -6.38E-02 | 2.23E-05 | 8.98E-04 | -3.18E-01 |
| cg06196147 | KIAA1257 | TSS200 | Island | 1.61E-02 | 1.95E-02 | 4.09E-03 | 2.28E-05 | 9.10E-04 | 9.12E+00 |
| cg01397223 |  |  | OpenSea | 8.95E-01 | 8.72E-01 | -5.19E-02 | 2.31E-05 | 9.18E-04 | -2.15E+00 |
| cg15765936 | C14orf132 | 3'UTR | OpenSea | 8.34E-01 | 8.54E-01 | 3.85E-02 | 2.33E-05 | 9.22E-04 | -2.41E+00 |
| cg25301776 | WWOX | Body | OpenSea | 9.06E-01 | 9.24E-01 | 1.66E-02 | 2.35E-05 | 9.25E-04 | 2.23E+00 |
| cg22972126 | SLC35F5 | TSS200 | Island | 4.24E-02 | 5.33E-02 | 9.40E-03 | 2.37E-05 | 9.30E-04 | 2.51E+00 |
| cg18840214 |  |  | OpenSea | 9.32E-01 | 9.61E-01 | 7.04E-02 | 2.40E-05 | 9.38E-04 | 6.22E-03 |
| cg05236660 | MARVELD3 | TSS200 | Island | 6.20E-02 | 7.89E-02 | 1.21E-02 | 2.43E-05 | 9.44E-04 | 9.02E+00 |
| cg19719150 | LPIN3;LPIN3 | Body | OpenSea | 9.61E-01 | 9.70E-01 | 5.61E-03 | 2.48E-05 | 9.56E-04 | 5.99E+00 |
| cg21312906 | SLC5A11 | 5'UTR;TSS200 | OpenSea | 8.37E-01 | 8.74E-01 | 4.98E-02 | 2.48E-05 | 9.56E-04 | 4.61E-02 |
| cg20826740 |  |  | N_Shore | 6.39E-02 | 1.22E-01 | 1.23E-01 | 2.51E-05 | 9.63E-04 | 6.25E-01 |
| cg09685173 |  |  | OpenSea | 7.60E-01 | 7.97E-01 | 2.65E-02 | 2.53E-05 | 9.68E-04 | 2.37E+00 |
| cg17893520 | GPR82;CASK | TSS1500;Body | OpenSea | 5.22E-01 | 4.65E-01 | -6.34E-02 | 2.62E-05 | 9.86E-04 | -1.21E+00 |
| cg14022913 | NEIL3 | TSS1500 | N_Shore | 1.48E-01 | 1.83E-01 | 3.37E-02 | 2.67E-05 | 9.97E-04 | -8.36E-02 |
| cg14910288 |  |  | S_Shore | 7.36E-01 | 6.90E-01 | 5.03E-03 | 2.68E-05 | 9.99E-04 | -1.38E-01 |
| cg05143886 |  |  | OpenSea | 7.58E-01 | 7.96E-01 | 5.09E-02 | 2.71E-05 | 1.01E-03 | 3.25E-01 |
| cg14776780 | CNOT4 | Body | OpenSea | 9.63E-01 | 9.76E-01 | 2.28E-02 | 2.75E-05 | 1.02E-03 | 5.50E+00 |
| cg22574280 | ACTG2 | TSS1500 | OpenSea | 9.62E-01 | 9.73E-01 | 2.95E-02 | 2.77E-05 | 1.02E-03 | 2.27E-02 |
| cg04998798 | TFDP1 | Body | OpenSea | 9.16E-01 | 9.32E-01 | 1.05E-02 | 2.81E-05 | 1.03E-03 | 8.09E-01 |
| cg05421651 | SNORD115-9;SNORD115-5;  SNORD115-12;SNORD115-10 | TSS1500 | OpenSea | 8.77E-01 | 9.12E-01 | 6.72E-02 | 2.82E-05 | 1.03E-03 | 1.10E+00 |
| cg17104512 | WDR60 | Body | N_Shelf | 5.66E-01 | 6.05E-01 | 6.38E-02 | 2.89E-05 | 1.05E-03 | 7.88E-02 |
| cg24886799 |  |  | OpenSea | 9.64E-01 | 9.58E-01 | -9.36E-03 | 2.89E-05 | 1.05E-03 | -1.04E+01 |
| cg19233880 | CAMK2G | Body | N_Shore | 9.82E-01 | 9.87E-01 | 1.07E-02 | 2.97E-05 | 1.06E-03 | 7.19E-01 |
| cg11740845 | CCDC42;CCDC42 | Body | OpenSea | 9.84E-01 | 9.88E-01 | 3.44E-03 | 3.03E-05 | 1.08E-03 | 2.06E+01 |
| cg02133183 | FAM190B | TSS200 | N_Shore | 3.35E-02 | 3.91E-02 | 6.67E-03 | 3.04E-05 | 1.08E-03 | 5.77E+00 |
| cg04243215 |  |  | OpenSea | 9.56E-01 | 9.37E-01 | -2.48E-02 | 3.18E-05 | 1.11E-03 | -1.76E+00 |
| cg20976673 |  |  | OpenSea | 4.76E-01 | 3.93E-01 | -1.96E-01 | 3.23E-05 | 1.12E-03 | -6.44E-01 |
| cg07383415 | FAM49A | 5'UTR | OpenSea | 3.95E-01 | 2.98E-01 | -2.25E-01 | 3.26E-05 | 1.13E-03 | -4.83E-02 |
| cg09970539 | CCR2;CCR2 | 5'UTR | OpenSea | 9.56E-01 | 9.41E-01 | -2.77E-02 | 3.31E-05 | 1.14E-03 | -2.21E+00 |
| cg01715379 | ZNF783 | Body | Island | 9.54E-01 | 9.63E-01 | 7.65E-03 | 3.40E-05 | 1.16E-03 | 9.89E+00 |
| cg08241401 |  |  | N_Shelf | 8.85E-01 | 9.12E-01 | 7.54E-02 | 3.42E-05 | 1.16E-03 | 5.25E-01 |
| cg06080874 |  |  | Island | 8.92E-02 | 1.20E-01 | 2.87E-02 | 3.55E-05 | 1.19E-03 | 7.06E-01 |
| cg18177178 | OGG1 | 5'UTR;1stExon | Island | 1.77E-02 | 2.13E-02 | 3.19E-03 | 3.60E-05 | 1.20E-03 | 6.53E+00 |
| cg21770873 | ELP5 | Body | S_Shore | 9.52E-01 | 9.35E-01 | -4.30E-02 | 3.61E-05 | 1.20E-03 | -1.40E+00 |
| cg18717554 | STRA6 | 5'UTR;1stExon;Body | OpenSea | 6.51E-01 | 6.01E-01 | -1.23E-01 | 3.61E-05 | 1.20E-03 | -1.28E-01 |
| cg09947609 | FZD6 | TSS1500;5'UTR;1stExon | N_Shore | 2.14E-02 | 3.16E-02 | 6.93E-03 | 3.62E-05 | 1.20E-03 | 2.78E+00 |
| cg05884115 | PRKD2 | Body | Island | 9.84E-01 | 9.88E-01 | 3.51E-03 | 3.70E-05 | 1.22E-03 | 8.49E+00 |
| cg23588289 |  |  | OpenSea | 9.10E-01 | 9.29E-01 | 3.50E-02 | 3.74E-05 | 1.22E-03 | -2.92E+00 |
| cg10017964 |  |  | OpenSea | 6.34E-01 | 5.36E-01 | -1.31E-01 | 3.77E-05 | 1.23E-03 | -7.44E-02 |
| cg16885277 |  |  | OpenSea | 6.00E-01 | 6.20E-01 | 7.85E-03 | 3.77E-05 | 1.23E-03 | 4.37E-01 |
| cg06378607 | PUS1;PUS1;PUS1 | Body | N_Shelf | 8.68E-01 | 8.84E-01 | 1.66E-02 | 3.80E-05 | 1.24E-03 | 1.02E-01 |
| cg04568189 | CDK6;CDK6 | Body | OpenSea | 7.78E-01 | 7.30E-01 | -8.08E-02 | 3.80E-05 | 1.24E-03 | 3.15E-01 |
| cg07230975 |  |  | OpenSea | 9.19E-01 | 9.37E-01 | 2.95E-02 | 3.80E-05 | 1.24E-03 | -8.81E-01 |
| cg01126993 | LOC728730 | Body | OpenSea | 5.26E-01 | 4.61E-01 | -1.22E-01 | 3.86E-05 | 1.25E-03 | -8.17E-02 |
| cg04837991 | MFSD9 | Body | OpenSea | 9.79E-01 | 9.84E-01 | 4.12E-03 | 3.91E-05 | 1.26E-03 | 2.74E+00 |
| cg16473757 | PGBD2;PGBD2 | TSS1500 | N_Shore | 8.73E-01 | 8.49E-01 | -2.27E-02 | 4.03E-05 | 1.28E-03 | -2.57E-01 |
| cg10508185 | CHN1;CHN1 | Body | OpenSea | 8.47E-01 | 8.69E-01 | 4.01E-02 | 4.04E-05 | 1.28E-03 | -1.59E-01 |
| cg11007492 | DOCK1;FAM196A | Body | Island | 9.71E-01 | 9.75E-01 | 2.70E-03 | 4.04E-05 | 1.28E-03 | 1.44E+01 |
| cg21932190 | CDK11A;CDK11B | Body;5'UTR | S_Shore | 8.71E-01 | 8.98E-01 | 3.50E-02 | 4.10E-05 | 1.29E-03 | 4.75E+00 |
| cg13309406 | KIAA0802 | TSS1500 | OpenSea | 9.14E-01 | 9.29E-01 | 2.31E-02 | 4.12E-05 | 1.30E-03 | 4.39E+00 |
| cg01359995 |  |  | OpenSea | 8.43E-01 | 8.60E-01 | 2.12E-02 | 4.13E-05 | 1.30E-03 | 3.99E+00 |
| cg06030104 |  |  | OpenSea | 9.64E-01 | 9.73E-01 | 1.37E-02 | 4.16E-05 | 1.30E-03 | 3.85E+00 |
| cg15018149 | HTRA3;HTRA3 | Body | OpenSea | 7.96E-01 | 8.33E-01 | 6.22E-02 | 4.18E-05 | 1.31E-03 | 5.00E-01 |
| cg24229579 | NADSYN1 | Body | OpenSea | 9.08E-01 | 9.23E-01 | 1.65E-02 | 4.21E-05 | 1.31E-03 | 6.34E-01 |
| cg11848677 | POLA1 | Body | OpenSea | 9.44E-01 | 9.30E-01 | -2.73E-02 | 4.21E-05 | 1.31E-03 | -3.14E+00 |
| cg00577786 | C11orf60 | 5'UTR;1stExon | OpenSea | 1.69E-02 | 1.91E-02 | 2.66E-03 | 4.23E-05 | 1.32E-03 | 1.92E+01 |
| cg11964549 | ACY3 | Body | OpenSea | 9.79E-01 | 9.84E-01 | 4.92E-03 | 4.27E-05 | 1.33E-03 | 3.59E+00 |
| cg06783981 | KRTAP5-11;KRTAP5-11 | 1stExon;3'UTR | OpenSea | 7.65E-01 | 7.97E-01 | 4.93E-02 | 4.33E-05 | 1.34E-03 | -2.70E-01 |
| cg17472643 | CASK | Body | OpenSea | 4.64E-02 | 5.46E-02 | 1.97E-02 | 4.44E-05 | 1.36E-03 | 2.12E+00 |
| cg22350056 | HIST2H2AC;HIST2H2BE | TSS200;TSS1500 | Island | 1.99E-02 | 2.31E-02 | 4.28E-03 | 4.59E-05 | 1.38E-03 | 1.49E+01 |
| cg13723078 |  | 5'UTR | OpenSea | 6.99E-01 | 6.15E-01 | -1.43E-01 | 4.60E-05 | 1.38E-03 | -1.77E-01 |
| cg11620984 | PLSCR4 |  | N_Shore | 9.58E-02 | 1.23E-01 | 3.59E-02 | 4.79E-05 | 1.42E-03 | -1.46E+00 |
| cg13488811 |  |  | OpenSea | 1.72E-01 | 2.33E-01 | 1.03E-01 | 4.84E-05 | 1.42E-03 | 4.93E-01 |
| cg12940420 | HM13;HM13;HM13 | 3'UTR | N_Shelf | 9.62E-01 | 9.55E-01 | -1.76E-02 | 4.86E-05 | 1.43E-03 | -1.47E+01 |
| cg16471539 |  |  | S_Shore | 9.50E-01 | 9.23E-01 | -5.91E-02 | 4.88E-05 | 1.43E-03 | -2.13E+00 |
| cg02821342 | MKLN1;FLJ43663 | TSS1500;Body | N_Shore | 1.34E-01 | 8.62E-02 | -1.08E-01 | 4.88E-05 | 1.43E-03 | -7.43E-01 |
| cg13156086 | GREB1;GREB1 | ExonBnd;Body | OpenSea | 9.64E-01 | 9.74E-01 | 1.29E-02 | 4.91E-05 | 1.44E-03 | 2.22E+00 |
| cg19970124 | CLDN7;CLDN7;CLDN7 | TSS1500 | S_Shore | 2.21E-01 | 1.97E-01 | -4.02E-02 | 5.00E-05 | 1.45E-03 | -3.32E+00 |
| cg13834737 |  |  | OpenSea | 8.42E-01 | 8.70E-01 | 5.11E-02 | 5.05E-05 | 1.46E-03 | -4.49E-01 |
| cg01497311 | BIK | 5'UTR | S_Shelf | 8.17E-01 | 7.61E-01 | -1.19E-01 | 5.07E-05 | 1.46E-03 | -2.31E-01 |
| cg13608744 |  |  | OpenSea | 8.27E-01 | 8.56E-01 | 4.59E-02 | 5.18E-05 | 1.48E-03 | 4.10E-01 |
| cg08195383 |  |  | OpenSea | 1.28E-02 | 1.47E-02 | 2.90E-03 | 5.23E-05 | 1.49E-03 | 3.52E+01 |
| cg25818214 | PRICKLE2 | TSS200 | OpenSea | 5.86E-01 | 5.27E-01 | -1.45E-01 | 5.28E-05 | 1.50E-03 | -1.96E-01 |
| cg09638248 | ITGA8 | Body | OpenSea | 9.39E-01 | 9.51E-01 | 1.89E-02 | 5.37E-05 | 1.52E-03 | 4.36E+00 |
| cg08379158 |  |  | N_Shelf | 9.77E-01 | 9.82E-01 | 6.34E-03 | 5.38E-05 | 1.52E-03 | 1.92E+01 |
| cg04414026 |  |  | S_Shore | 5.69E-01 | 5.18E-01 | -3.03E-02 | 5.40E-05 | 1.52E-03 | -3.04E-02 |
| cg09667960 |  |  | OpenSea | 7.90E-01 | 8.28E-01 | 1.19E-01 | 5.41E-05 | 1.52E-03 | 5.08E-01 |
| cg04396421 | CYP4F3 | TSS1500 | OpenSea | 9.20E-01 | 9.00E-01 | -2.91E-03 | 5.58E-05 | 1.55E-03 | -2.33E+00 |
| cg03771939 | TBC1D12 | TSS1500 | N_Shore | 6.84E-01 | 7.38E-01 | 2.30E-02 | 5.62E-05 | 1.56E-03 | 2.83E-03 |
| cg20020221 |  |  | N_Shore | 9.65E-01 | 9.78E-01 | 3.43E-03 | 5.96E-05 | 1.61E-03 | 9.80E+00 |
| cg23284109 | STAC;STAC | TSS1500 | Island | 3.77E-01 | 4.13E-01 | 4.36E-02 | 5.96E-05 | 1.61E-03 | 3.19E-01 |
| cg05878311 | LRFN2 | 5'UTR | OpenSea | 9.07E-01 | 9.24E-01 | 2.73E-02 | 5.99E-05 | 1.61E-03 | 3.89E-01 |
| cg22467473 |  |  | OpenSea | 8.07E-01 | 7.49E-01 | -8.41E-02 | 6.14E-05 | 1.64E-03 | 9.43E-01 |
| cg05039538 |  |  | OpenSea | 8.48E-02 | 1.09E-01 | -4.90E-03 | 6.17E-05 | 1.65E-03 | 8.01E+00 |
| cg13421543 |  |  | OpenSea | 9.16E-01 | 9.37E-01 | 4.20E-02 | 6.29E-05 | 1.66E-03 | -1.34E+00 |
| cg09071762 | DPPA5 | TSS200 | Island | 8.09E-01 | 8.51E-01 | 5.78E-02 | 6.31E-05 | 1.67E-03 | 9.80E-01 |
| cg24641214 | MOGAT3 | 1stExon;5'UTR | N_Shore | 5.64E-01 | 5.82E-01 | 2.35E-02 | 6.33E-05 | 1.67E-03 | 1.97E+00 |
| cg23047271 | PRICKLE2;PRICKLE2 | 1stExon;5'UTR | OpenSea | 1.41E-01 | 1.05E-01 | -6.58E-02 | 6.52E-05 | 1.70E-03 | -6.30E-01 |
| cg06024252 | ENAM | Body | OpenSea | 6.72E-01 | 6.15E-01 | -6.13E-02 | 6.54E-05 | 1.70E-03 | 4.52E-01 |
| cg16638163 |  |  | OpenSea | 9.30E-01 | 9.46E-01 | 1.23E-02 | 6.61E-05 | 1.71E-03 | 2.14E+00 |
| cg13598366 | GREM2;MIR1273E | 5'UTR;Body | OpenSea | 8.23E-01 | 7.95E-01 | -9.47E-02 | 6.70E-05 | 1.73E-03 | -8.87E-01 |
| cg24510437 | SLC2A7 | Body | OpenSea | 9.63E-01 | 9.78E-01 | 1.54E-02 | 6.87E-05 | 1.75E-03 | 3.03E-01 |
| cg10809449 | COL6A1 | Body | Island | 8.60E-01 | 8.71E-01 | 1.90E-02 | 7.02E-05 | 1.78E-03 | 5.64E+00 |
| cg12436952 |  |  | OpenSea | 9.52E-01 | 8.88E-01 | -1.98E-01 | 7.07E-05 | 1.79E-03 | -1.16E-01 |
| cg08430662 |  |  | OpenSea | 9.43E-01 | 9.58E-01 | 1.40E-02 | 7.20E-05 | 1.80E-03 | 1.01E+00 |
| cg16883944 | PMP22 | Body | N_Shore | 4.23E-02 | 5.13E-02 | 1.60E-02 | 7.21E-05 | 1.81E-03 | -3.65E+00 |
| cg03323998 |  |  | S_Shelf | 6.50E-01 | 6.75E-01 | 3.68E-02 | 7.45E-05 | 1.84E-03 | 6.84E-01 |
| cg12221118 | ATP5A1 | Body | OpenSea | 9.81E-01 | 9.87E-01 | 8.49E-03 | 7.68E-05 | 1.88E-03 | 2.03E+01 |
| cg21737434 | CNTN1 | TSS1500;5'UTR | OpenSea | 6.26E-02 | 8.58E-02 | 4.28E-02 | 7.69E-05 | 1.88E-03 | 2.76E+00 |
| cg09356775 | CMTM8 | Body | OpenSea | 7.29E-01 | 6.70E-01 | -1.09E-01 | 7.80E-05 | 1.89E-03 | 4.23E-01 |
| cg07402639 | CCR4 | Body | OpenSea | 9.24E-01 | 9.04E-01 | -2.99E-02 | 7.97E-05 | 1.92E-03 | 1.94E+00 |
| cg04472373 |  |  | OpenSea | 8.41E-01 | 8.19E-01 | -2.49E-02 | 8.11E-05 | 1.94E-03 | 2.81E-01 |
| cg04652943 |  |  | N_Shore | 7.40E-01 | 7.88E-01 | 1.21E-02 | 8.13E-05 | 1.94E-03 | 1.07E+00 |
| cg23001647 | TMEM212-AS1 | TSS1500 | OpenSea | 9.69E-01 | 9.10E-01 | -2.16E-02 | 8.23E-05 | 1.96E-03 | -1.89E+00 |
| cg02959588 |  |  | OpenSea | 4.45E-02 | 5.04E-02 | 2.33E-02 | 8.23E-05 | 1.96E-03 | 3.22E+00 |
| cg22913070 |  |  | OpenSea | 9.38E-01 | 9.50E-01 | 2.48E-02 | 8.25E-05 | 1.96E-03 | -2.63E+00 |
| cg16375999 |  |  | OpenSea | 7.97E-01 | 8.35E-01 | 5.31E-02 | 8.41E-05 | 1.98E-03 | 6.25E-01 |
| cg11421827 | ASAP1;ASAP1 | 5'UTR;Body | OpenSea | 9.83E-01 | 9.86E-01 | 1.18E-03 | 8.56E-05 | 2.00E-03 | 1.20E+01 |
| cg04049894 | JOSD2;ASPDH | TSS1500;Body;ExonBnd | S_Shore | 9.46E-01 | 9.55E-01 | 7.03E-03 | 8.61E-05 | 2.01E-03 | 3.60E+00 |
| cg24550369 |  |  | OpenSea | 7.93E-01 | 8.21E-01 | 4.23E-02 | 8.62E-05 | 2.01E-03 | -1.51E+00 |
| cg03363012 | C20orf54 | 3'UTR | N_Shore | 9.35E-01 | 9.52E-01 | 2.54E-02 | 8.66E-05 | 2.01E-03 | 2.63E+00 |
| cg01446620 |  |  | N_Shelf | 3.05E-01 | 2.72E-01 | -2.40E-02 | 8.80E-05 | 2.04E-03 | -1.19E-01 |
| cg09700233 | CHAF1A | Body | Island | 1.75E-02 | 1.93E-02 | 1.91E-03 | 8.87E-05 | 2.04E-03 | 4.60E+01 |
| cg25843174 | TEAD1 | Body | OpenSea | 8.11E-01 | 8.38E-01 | 3.85E-02 | 8.91E-05 | 2.05E-03 | 1.97E+00 |
| cg16405492 | VSIG1;VSIG1 | TSS1500 | OpenSea | 5.72E-01 | 5.98E-01 | 4.90E-02 | 8.93E-05 | 2.05E-03 | 3.05E-01 |
| cg17737146 | LRRC34 | Body | Island | 9.81E-02 | 1.24E-01 | 2.83E-02 | 8.94E-05 | 2.05E-03 | 1.70E-01 |
| cg02922675 | ZNF423;ZNF423 | Body | OpenSea | 9.33E-01 | 9.46E-01 | 2.28E-02 | 9.12E-05 | 2.08E-03 | -4.90E+00 |
| cg25310906 | BRD3 | Body | N_Shore | 9.26E-01 | 9.37E-01 | 1.05E-02 | 9.13E-05 | 2.08E-03 | 1.17E+00 |
| cg25844969 | ZNF462;MIR548Q;ZNF462 | ExonBnd;Body | OpenSea | 9.60E-01 | 9.53E-01 | -9.44E-03 | 9.16E-05 | 2.09E-03 | -4.24E-01 |
| cg13212668 | CDH4 | Body | OpenSea | 4.80E-01 | 5.27E-01 | 1.76E-02 | 9.27E-05 | 2.10E-03 | 1.49E+00 |
| cg10526559 | SACS | Body | S_Shelf | 3.74E-02 | 4.82E-02 | 1.94E-02 | 9.32E-05 | 2.11E-03 | -2.20E+00 |
| cg21440284 |  |  | OpenSea | 9.54E-01 | 9.71E-01 | 2.41E-02 | 9.35E-05 | 2.11E-03 | 3.62E+00 |
| cg13753515 | SIGLEC1 | TSS200 | OpenSea | 9.08E-01 | 9.28E-01 | 3.22E-02 | 9.38E-05 | 2.12E-03 | -1.63E+00 |
| cg07368796 |  |  | N_Shore | 7.57E-02 | 8.38E-02 | 1.02E-02 | 9.39E-05 | 2.12E-03 | 6.15E-01 |
| cg13556110 |  |  | OpenSea | 3.90E-01 | 4.32E-01 | 3.33E-02 | 9.41E-05 | 2.12E-03 | 2.30E+00 |
| cg14018959 | EHD3 | Body | OpenSea | 9.38E-01 | 9.51E-01 | 2.34E-02 | 9.44E-05 | 2.12E-03 | 1.45E+00 |
| cg09173378 | KCNT2 | Body | OpenSea | 5.61E-01 | 4.99E-01 | -1.72E-01 | 9.86E-05 | 2.18E-03 | -1.27E+00 |
| cg19045293 |  |  | Island | 1.66E-01 | 2.16E-01 | 5.36E-02 | 9.91E-05 | 2.19E-03 | 1.68E+00 |
| cg23039250 | TLR5 | 5'UTR | OpenSea | 6.53E-01 | 7.17E-01 | 9.32E-02 | 9.99E-05 | 2.20E-03 | 3.27E-01 |
| cg08494871 | IQCK | Body | OpenSea | 8.63E-01 | 8.82E-01 | 2.34E-02 | 9.99E-05 | 2.20E-03 | -1.53E+00 |
| cg16430332 | C2orf74;C2orf74 | TSS1500;5'UTR | OpenSea | 9.73E-01 | 9.77E-01 | 6.69E-03 | 1.01E-04 | 2.21E-03 | 6.03E+00 |
| cg00669733 | SDK2 | Body | OpenSea | 9.82E-01 | 9.71E-01 | -5.59E-03 | 1.01E-04 | 2.22E-03 | -1.64E+00 |
| cg19817177 |  |  | OpenSea | 2.96E-02 | 3.86E-02 | 8.13E-03 | 1.02E-04 | 2.22E-03 | 1.82E+00 |
| cg19364311 | LOC101928402;STAG2 | TSS1500;5'UTR | S_Shore | 7.06E-01 | 6.60E-01 | -6.92E-02 | 1.03E-04 | 2.24E-03 | -4.01E-02 |
| cg26237631 | TRAPPC9;TRAPPC9 | Body | OpenSea | 9.55E-01 | 9.66E-01 | 1.48E-02 | 1.05E-04 | 2.26E-03 | -2.92E+00 |
| cg16768784 |  |  | OpenSea | 6.15E-01 | 5.61E-01 | -1.42E-01 | 1.05E-04 | 2.26E-03 | -1.25E-01 |
| cg01652244 | DPPA5 | TSS200 | Island | 8.85E-01 | 9.29E-01 | 5.94E-02 | 1.05E-04 | 2.26E-03 | 1.38E+00 |
| cg09964091 | COL13A1 | Body | OpenSea | 9.42E-01 | 9.57E-01 | 2.27E-02 | 1.05E-04 | 2.26E-03 | -4.47E+00 |
| cg07212142 |  |  | OpenSea | 7.97E-01 | 7.52E-01 | -7.68E-02 | 1.07E-04 | 2.29E-03 | -8.08E-01 |
| cg02857405 |  |  | S_Shelf | 7.95E-01 | 8.36E-01 | 5.75E-02 | 1.07E-04 | 2.29E-03 | 5.84E-01 |
| cg02323744 | ATP2C2;ATP2C2 | Body | S_Shelf | 6.13E-01 | 5.37E-01 | -2.11E-01 | 1.08E-04 | 2.30E-03 | -1.17E+00 |
| cg24162150 | STX16-NPEPL1;NPEPL1 | Body | OpenSea | 9.22E-01 | 9.35E-01 | 2.46E-02 | 1.08E-04 | 2.31E-03 | -4.59E-01 |
| cg02102700 |  |  | OpenSea | 9.43E-01 | 9.55E-01 | 1.93E-02 | 1.09E-04 | 2.32E-03 | 4.44E+00 |
| cg17037491 | LRRC16A | Body | S_Shore | 5.38E-02 | 6.34E-02 | 1.46E-02 | 1.11E-04 | 2.35E-03 | 8.11E+00 |
| cg03358982 | ANTXR2 | 5'UTR;1stExon | Island | 2.69E-02 | 3.06E-02 | 4.35E-03 | 1.12E-04 | 2.36E-03 | 1.46E+00 |
| cg18537687 | RALGAPA2 | Body | OpenSea | 8.27E-01 | 8.50E-01 | 6.00E-02 | 1.12E-04 | 2.36E-03 | -5.03E-01 |
| cg07499835 | RSRC1;RSRC1;RSRC1 | Body | OpenSea | 6.73E-01 | 6.20E-01 | -7.84E-02 | 1.12E-04 | 2.36E-03 | -6.83E-02 |
| cg17252538 | VIPR2;VIPR2;VIPR2 | Body | OpenSea | 5.69E-01 | 6.05E-01 | 2.14E-02 | 1.13E-04 | 2.38E-03 | 1.91E-01 |
| cg02949436 | TCF25 | Body | S_Shore | 1.25E-02 | 1.50E-02 | 3.19E-03 | 1.14E-04 | 2.39E-03 | 2.79E+01 |
| cg05652143 | LOC102467080;LOC102467080 | Body | OpenSea | 9.05E-01 | 8.66E-01 | -7.85E-02 | 1.16E-04 | 2.40E-03 | -8.77E-01 |
| cg27412899 |  |  | OpenSea | 1.22E-01 | 1.37E-01 | 2.05E-03 | 1.19E-04 | 2.44E-03 | 1.70E+00 |
| cg09025741 |  |  | OpenSea | 7.80E-01 | 7.30E-01 | 6.40E-03 | 1.19E-04 | 2.44E-03 | -2.00E-02 |
| cg02718199 | TBXAS1 | Body | OpenSea | 9.82E-01 | 9.76E-01 | -5.36E-03 | 1.20E-04 | 2.46E-03 | -5.97E+00 |
| cg23738770 | NFKB2;NFKB2;NFKB2 | TSS1500;5'UTR | Island | 1.20E-02 | 1.40E-02 | 2.18E-03 | 1.21E-04 | 2.47E-03 | 6.91E+01 |
| cg18638769 | PACS2;PACS2 | Body | OpenSea | 9.43E-01 | 9.38E-01 | -9.68E-03 | 1.22E-04 | 2.48E-03 | -2.02E+00 |
| cg11535059 |  |  | OpenSea | 5.91E-01 | 6.24E-01 | -9.62E-03 | 1.22E-04 | 2.48E-03 | 1.87E+00 |
| cg14051886 | C20orf114 | TSS1500 | OpenSea | 9.56E-01 | 9.67E-01 | 2.25E-02 | 1.23E-04 | 2.49E-03 | 4.48E+00 |
| cg05191175 |  |  | N_Shore | 2.20E-01 | 2.48E-01 | 6.96E-02 | 1.24E-04 | 2.51E-03 | 3.07E-01 |
| cg08614242 |  |  | S_Shore | 4.62E-01 | 5.00E-01 | 4.88E-02 | 1.24E-04 | 2.51E-03 | 1.58E+00 |
| cg26248430 | C2orf74;C2orf74 | Body | OpenSea | 9.74E-01 | 9.80E-01 | 5.32E-03 | 1.24E-04 | 2.51E-03 | 2.95E+00 |
| cg07627692 | NLGN1 | Body | OpenSea | 7.16E-01 | 6.44E-01 | -1.58E-01 | 1.26E-04 | 2.53E-03 | -1.38E-01 |
| cg02401149 | CRYBB3 | TSS1500 | OpenSea | 9.26E-01 | 9.39E-01 | 1.03E-02 | 1.27E-04 | 2.54E-03 | 2.20E+00 |
| cg22875643 | MIER1 | TSS1500;5'UTR;Body | N_Shore | 1.75E-02 | 2.38E-02 | 4.89E-03 | 1.27E-04 | 2.54E-03 | 7.36E+00 |
| cg04149356 | EBF1 | Body | OpenSea | 9.69E-01 | 9.59E-01 | -4.58E-03 | 1.28E-04 | 2.55E-03 | -4.61E+00 |
| cg22009500 |  |  | S_Shore | 7.10E-02 | 5.95E-02 | -2.23E-02 | 1.28E-04 | 2.55E-03 | -1.04E+01 |
| cg00807297 | CTDP1;CTDP1 | Body | N_Shelf | 9.17E-01 | 9.32E-01 | 1.96E-02 | 1.28E-04 | 2.56E-03 | -3.21E+00 |
| cg03433510 | BOLL;BOLL | Body;TSS200 | Island | 9.38E-01 | 9.56E-01 | 2.73E-02 | 1.32E-04 | 2.60E-03 | 9.28E-01 |
| cg14506567 |  |  | OpenSea | 7.02E-01 | 7.30E-01 | -3.59E-03 | 1.33E-04 | 2.61E-03 | 1.61E+00 |
| cg19892234 | IGF2BP2 | Body;5'UTR | OpenSea | 1.17E-01 | 8.86E-02 | -6.26E-02 | 1.33E-04 | 2.61E-03 | -3.79E-03 |
| cg16141421 |  |  | N_Shore | 9.10E-01 | 9.27E-01 | 1.95E-02 | 1.36E-04 | 2.64E-03 | 2.71E+00 |
| cg04288975 |  |  | S_Shelf | 9.74E-02 | 1.27E-01 | 3.31E-02 | 1.37E-04 | 2.66E-03 | -8.00E-01 |
| cg26647332 |  |  | N_Shore | 7.03E-02 | 8.57E-02 | 1.38E-02 | 1.37E-04 | 2.66E-03 | -1.92E+00 |
| cg19069039 |  |  | N_Shelf | 9.41E-01 | 9.51E-01 | 1.71E-02 | 1.39E-04 | 2.68E-03 | -6.85E-01 |
| cg04229103 | GTDC1 | TSS200 | Island | 5.25E-02 | 6.29E-02 | 6.35E-03 | 1.39E-04 | 2.68E-03 | 4.71E-01 |
| cg00169677 | ZC3H3 | Body | OpenSea | 9.18E-01 | 9.28E-01 | 7.42E-03 | 1.40E-04 | 2.69E-03 | 3.99E+00 |
| cg27041937 |  |  | OpenSea | 4.88E-01 | 4.48E-01 | 4.39E-03 | 1.41E-04 | 2.71E-03 | -1.18E+00 |
| cg19185932 | VWA3B | Body | OpenSea | 9.14E-01 | 9.40E-01 | 6.29E-02 | 1.44E-04 | 2.73E-03 | 1.66E+00 |
| cg15506591 |  |  | S_Shore | 2.23E-01 | 2.46E-01 | 3.52E-02 | 1.44E-04 | 2.74E-03 | 3.53E+00 |
| cg04444758 | DPP4 | Body | OpenSea | 7.92E-01 | 7.52E-01 | -5.90E-02 | 1.44E-04 | 2.74E-03 | 1.87E-01 |
| cg16247544 | CMSS1 | Body | OpenSea | 7.80E-01 | 7.21E-01 | -6.07E-02 | 1.45E-04 | 2.74E-03 | 3.31E-01 |
| cg18153747 |  |  | OpenSea | 9.50E-01 | 9.63E-01 | 2.55E-02 | 1.46E-04 | 2.76E-03 | 5.08E+00 |
| cg14778349 | PLAC1 | 5'UTR | OpenSea | 7.40E-01 | 7.77E-01 | 4.86E-02 | 1.50E-04 | 2.80E-03 | 2.30E+00 |
| cg26790897 | SUMF2 | 3'UTR | OpenSea | 4.60E-01 | 4.23E-01 | -7.95E-02 | 1.51E-04 | 2.82E-03 | -2.04E+00 |
| cg12444542 | CACNA2D4 | Body | OpenSea | 9.37E-01 | 9.51E-01 | 9.24E-03 | 1.53E-04 | 2.84E-03 | 9.70E-01 |
| cg06448666 | MID1 | 5'UTR;1stExon | OpenSea | 6.89E-01 | 6.59E-01 | -2.90E-02 | 1.53E-04 | 2.84E-03 | -5.79E-01 |
| cg14854573 | PAQR5 | Body | OpenSea | 9.85E-01 | 9.88E-01 | 3.55E-03 | 1.54E-04 | 2.85E-03 | 1.74E+00 |
| cg15717081 | PSD3 | Body | OpenSea | 3.00E-01 | 2.36E-01 | -8.57E-02 | 1.55E-04 | 2.86E-03 | -8.87E-01 |
| cg11548059 | TRERF1 | Body | N_Shore | 9.50E-01 | 9.39E-01 | -3.37E-02 | 1.55E-04 | 2.86E-03 | -5.25E+00 |
| cg23012886 | LOC283038 | Body | OpenSea | 3.32E-02 | 4.23E-02 | 2.55E-03 | 1.56E-04 | 2.87E-03 | 1.16E+00 |
| cg11426563 |  |  | Island | 1.18E-01 | 1.08E-01 | -1.05E-02 | 1.58E-04 | 2.89E-03 | -1.00E+00 |
| cg24355850 | LOC400752 | TSS200 | Island | 9.81E-02 | 1.19E-01 | 2.60E-02 | 1.58E-04 | 2.89E-03 | 1.23E+00 |
| cg22360089 |  |  | OpenSea | 9.63E-01 | 9.74E-01 | 1.62E-02 | 1.59E-04 | 2.90E-03 | 2.82E+00 |
| cg24600611 | HCG2040054;KCNK12 | Body | OpenSea | 8.81E-01 | 9.08E-01 | 4.99E-02 | 1.59E-04 | 2.90E-03 | -1.24E+00 |
| cg01514943 | BCOR;BCOR | 5'UTR | N_Shore | 1.05E-01 | 1.37E-01 | 3.35E-02 | 1.60E-04 | 2.91E-03 | 1.56E+00 |
| cg01416168 | SLC5A9 | TSS1500 | OpenSea | 9.61E-01 | 9.53E-01 | -1.19E-02 | 1.60E-04 | 2.91E-03 | 4.44E-01 |
| cg24718526 | TTC15 | Body | N_Shelf | 9.56E-01 | 9.64E-01 | 1.02E-02 | 1.63E-04 | 2.95E-03 | -6.33E-01 |
| cg12880969 | CLU | Body | OpenSea | 8.58E-01 | 8.17E-01 | -1.37E-01 | 1.64E-04 | 2.95E-03 | -1.31E+00 |
| cg10526245 | GPR177 | Body | OpenSea | 9.73E-01 | 9.70E-01 | -4.44E-03 | 1.65E-04 | 2.96E-03 | -4.22E+00 |
| cg00454592 |  |  | OpenSea | 1.35E-01 | 1.73E-01 | 3.66E-02 | 1.65E-04 | 2.97E-03 | -4.91E-01 |
| cg18023674 | PLCH2 | Body | OpenSea | 7.98E-01 | 8.20E-01 | -1.36E-02 | 1.65E-04 | 2.97E-03 | 3.80E+00 |
| cg22701297 | SLC17A8 | Body | OpenSea | 4.10E-01 | 4.50E-01 | 7.80E-02 | 1.69E-04 | 3.01E-03 | 1.09E+00 |
| cg22163130 | FAM19A2 | TSS200 | S_Shore | 2.14E-02 | 2.55E-02 | 9.33E-03 | 1.70E-04 | 3.02E-03 | -1.67E+00 |
| cg04928670 |  |  | OpenSea | 6.69E-01 | 6.23E-01 | -1.03E-01 | 1.70E-04 | 3.02E-03 | -1.78E+00 |
| cg26775944 | DOK5 | Body | OpenSea | 1.92E-02 | 2.26E-02 | 4.62E-03 | 1.71E-04 | 3.03E-03 | 2.64E+00 |
| cg12289092 | NCAPH | Body | OpenSea | 9.69E-01 | 9.57E-01 | -1.03E-02 | 1.72E-04 | 3.04E-03 | -1.33E+00 |
| cg11769850 |  |  | N_Shore | 9.74E-01 | 9.69E-01 | -8.97E-03 | 1.73E-04 | 3.05E-03 | -5.62E+00 |
| cg26475687 | CDC42BPA | 3'UTR | OpenSea | 9.75E-01 | 9.72E-01 | -3.59E-03 | 1.74E-04 | 3.06E-03 | -1.08E+00 |
| cg14892359 |  |  | N_Shelf | 6.51E-01 | 7.33E-01 | 1.62E-01 | 1.75E-04 | 3.07E-03 | 1.49E-01 |
| cg23447239 | PTPRN2 | Body | S_Shelf | 5.95E-01 | 5.52E-01 | -8.74E-02 | 1.76E-04 | 3.08E-03 | -6.70E-01 |
| cg08053901 |  |  | OpenSea | 8.48E-01 | 8.76E-01 | 6.28E-02 | 1.76E-04 | 3.09E-03 | 2.22E+00 |
| cg00540540 | ODZ2 | Body | OpenSea | 9.41E-01 | 9.32E-01 | -3.28E-02 | 1.76E-04 | 3.09E-03 | -3.15E-02 |
| cg07944831 |  |  | OpenSea | 8.63E-01 | 8.87E-01 | 4.39E-02 | 1.80E-04 | 3.13E-03 | 3.39E-01 |
| cg09129772 | GMFG | TSS1500 | OpenSea | 6.75E-01 | 6.60E-01 | -2.61E-02 | 1.81E-04 | 3.14E-03 | -1.05E+00 |
| cg18458993 | ARG2 | TSS200 | Island | 2.78E-02 | 3.10E-02 | 1.34E-03 | 1.82E-04 | 3.15E-03 | 1.32E+00 |
| cg23953395 | ZNF259 | TSS200 | Island | 6.39E-02 | 8.04E-02 | 4.37E-03 | 1.82E-04 | 3.15E-03 | 1.33E-01 |
| cg08625777 | GSTCD | 5'UTR | OpenSea | 1.32E-01 | 1.52E-01 | 2.31E-02 | 1.83E-04 | 3.17E-03 | -6.71E-01 |
| cg21564998 | KITLG | Body | OpenSea | 9.79E-01 | 9.75E-01 | -4.80E-03 | 1.86E-04 | 3.19E-03 | -3.20E+00 |
| cg08827252 | PDZD2 | Body | OpenSea | 9.60E-01 | 9.67E-01 | 8.65E-03 | 1.87E-04 | 3.20E-03 | 2.40E-01 |
| cg04736673 | CCDC6 | Body | OpenSea | 3.23E-01 | 4.53E-01 | 2.11E-01 | 1.89E-04 | 3.23E-03 | 3.75E-01 |
| cg02051975 | CST8 | TSS200 | OpenSea | 9.61E-01 | 9.67E-01 | 8.71E-03 | 1.91E-04 | 3.25E-03 | 1.40E+00 |
| cg17847466 | KMT2E-AS1;KMT2E | Body;TSS1500 | Island | 4.12E-02 | 5.02E-02 | 8.83E-03 | 1.92E-04 | 3.25E-03 | 3.29E+00 |
| cg26096034 | VPS53 | Body | OpenSea | 9.59E-01 | 9.69E-01 | 2.20E-02 | 1.94E-04 | 3.27E-03 | 2.32E+00 |
| cg02049829 | UBL4B | TSS200 | OpenSea | 9.40E-01 | 9.50E-01 | 1.45E-02 | 2.01E-04 | 3.34E-03 | 2.80E+00 |
| cg22276264 | LINC00620 | Body | OpenSea | 8.14E-01 | 8.39E-01 | 2.78E-02 | 2.03E-04 | 3.36E-03 | 1.20E+00 |
| cg15725616 | GNAS;GNAS-AS1 | TSS1500;3'UTR;Body | Island | 5.44E-01 | 5.74E-01 | 2.16E-02 | 2.06E-04 | 3.39E-03 | -2.29E-03 |
| cg03008692 | PTPRN2 | Body | OpenSea | 9.23E-01 | 9.38E-01 | 2.60E-02 | 2.06E-04 | 3.39E-03 | 2.07E+00 |
| cg18512292 | NINL | 5'UTR | OpenSea | 9.80E-01 | 9.85E-01 | -1.93E-04 | 2.08E-04 | 3.42E-03 | 3.16E+00 |
| cg16632785 |  |  | OpenSea | 6.80E-01 | 7.43E-01 | 1.33E-01 | 2.09E-04 | 3.43E-03 | 5.96E-01 |
| cg19668256 | CDH4 | Body | N_Shelf | 7.68E-01 | 7.92E-01 | 2.24E-02 | 2.11E-04 | 3.44E-03 | 1.07E+00 |
| cg08219773 | EFHB | TSS200 | OpenSea | 5.48E-01 | 5.03E-01 | -1.11E-02 | 2.11E-04 | 3.44E-03 | -7.46E-01 |
| cg14431551 | TTC7A | Body | OpenSea | 9.01E-01 | 9.15E-01 | 2.01E-02 | 2.11E-04 | 3.44E-03 | -6.70E-01 |
| cg05791779 | CHCHD6 | Body | OpenSea | 8.84E-01 | 9.03E-01 | 1.49E-02 | 2.12E-04 | 3.45E-03 | -2.93E+00 |
| cg11907977 |  |  | OpenSea | 9.02E-01 | 9.20E-01 | 1.76E-02 | 2.12E-04 | 3.45E-03 | -1.36E+00 |
| cg08840950 | MGLL | Body | OpenSea | 9.79E-01 | 9.84E-01 | 6.04E-03 | 2.13E-04 | 3.46E-03 | 1.91E+01 |
| cg02544979 | TESPA1 | TSS1500;Body | OpenSea | 7.27E-01 | 7.51E-01 | 3.59E-02 | 2.15E-04 | 3.48E-03 | -3.45E-02 |
| cg22029284 | PRDM16 | Body | N_Shelf | 8.43E-01 | 8.69E-01 | 5.11E-02 | 2.15E-04 | 3.48E-03 | -7.98E-01 |
| cg02065192 | ACOT7 | TSS1500;Body | OpenSea | 9.54E-01 | 9.45E-01 | -1.20E-02 | 2.16E-04 | 3.49E-03 | -1.79E+01 |
| cg26947474 | COL4A6 | Body | OpenSea | 9.02E-01 | 8.87E-01 | -2.32E-02 | 2.19E-04 | 3.52E-03 | -2.13E+00 |
| cg12771165 | STRA8 | Body | Island | 9.86E-01 | 9.89E-01 | 4.76E-03 | 2.26E-04 | 3.58E-03 | 5.80E+00 |
| cg00280235 | NRCAM | 1stExon;5'UTR;Body | OpenSea | 1.52E-01 | 1.80E-01 | 3.68E-02 | 2.28E-04 | 3.60E-03 | -4.51E-03 |
| cg23963071 | SERPINB9 | 5'UTR | N_Shore | 9.14E-01 | 8.84E-01 | -9.64E-03 | 2.29E-04 | 3.62E-03 | -1.44E-01 |
| cg21949414 | FAM83B | Body | OpenSea | 9.62E-01 | 9.55E-01 | -3.40E-03 | 2.32E-04 | 3.65E-03 | -1.32E+00 |
| cg21746884 |  |  | OpenSea | 9.84E-01 | 9.88E-01 | 2.67E-03 | 2.33E-04 | 3.65E-03 | 2.33E+01 |
| cg18396637 | TSC22D3 | Body | N_Shelf | 6.88E-01 | 7.33E-01 | 6.30E-02 | 2.34E-04 | 3.66E-03 | 1.56E+00 |
| cg24953179 | EPDR1 | 1stExon | N_Shore | 2.23E-02 | 1.97E-02 | -4.25E-03 | 2.42E-04 | 3.74E-03 | -5.44E+00 |
| cg02095557 | DOCK10 | Body | OpenSea | 9.37E-01 | 9.19E-01 | -3.12E-02 | 2.42E-04 | 3.74E-03 | 6.73E-02 |
| cg14119069 | FHL1 | TSS1500;TSS200;Body;5'UTR | OpenSea | 5.87E-01 | 6.28E-01 | 4.41E-02 | 2.43E-04 | 3.75E-03 | 9.18E-01 |
| cg00016934 | BCOR | 5'UTR | Island | 1.02E-01 | 1.48E-01 | 4.05E-02 | 2.47E-04 | 3.79E-03 | 5.90E-01 |
| cg03311759 | RFX7 | Body | OpenSea | 8.38E-01 | 8.00E-01 | -5.04E-02 | 2.47E-04 | 3.79E-03 | 3.81E-01 |
| cg03172688 | PCDH1 | Body | N_Shore | 9.11E-01 | 8.70E-01 | -9.56E-02 | 2.48E-04 | 3.79E-03 | -1.76E+00 |
| cg11629619 |  |  | OpenSea | 1.38E-01 | 1.56E-01 | 2.64E-02 | 2.48E-04 | 3.79E-03 | -8.16E-02 |
| cg00525508 | ADARB2 | Body | OpenSea | 9.74E-01 | 9.78E-01 | 5.63E-03 | 2.51E-04 | 3.82E-03 | 1.04E+01 |
| cg06826636 |  |  | OpenSea | 8.74E-01 | 8.53E-01 | -7.50E-03 | 2.52E-04 | 3.83E-03 | -5.86E-01 |
| cg02433135 |  |  | OpenSea | 9.34E-01 | 9.49E-01 | 2.82E-02 | 2.54E-04 | 3.85E-03 | -1.23E+00 |
| cg11660537 | MEFV | Body | Island | 4.84E-01 | 5.20E-01 | 4.84E-02 | 2.55E-04 | 3.86E-03 | 2.26E-01 |
| cg23558994 | ZNF710 | 5'UTR | N_Shelf | 9.37E-01 | 9.12E-01 | -5.40E-02 | 2.56E-04 | 3.86E-03 | -4.83E-01 |
| cg20452975 |  |  | OpenSea | 9.57E-01 | 9.68E-01 | 9.98E-03 | 2.58E-04 | 3.88E-03 | 1.08E+01 |
| cg19104422 | PRTG | Body | N_Shore | 9.02E-02 | 1.14E-01 | 2.89E-02 | 2.63E-04 | 3.93E-03 | -3.79E+00 |
| cg22243637 | NFATC2 | Body | OpenSea | 8.50E-01 | 8.70E-01 | 5.20E-02 | 2.64E-04 | 3.94E-03 | -6.24E-01 |
| cg18429142 | GDPD5 | Body | OpenSea | 9.44E-01 | 9.58E-01 | 2.49E-02 | 2.66E-04 | 3.96E-03 | -2.04E+00 |
| cg06636678 |  |  | OpenSea | 3.37E-01 | 2.80E-01 | -1.06E-01 | 2.67E-04 | 3.97E-03 | -3.54E-01 |
| cg13671721 | FXN | Body | S_Shore | 9.59E-01 | 9.65E-01 | 3.15E-03 | 2.72E-04 | 4.01E-03 | 8.94E+00 |
| cg23202291 |  |  | S_Shore | 9.82E-01 | 9.85E-01 | 3.34E-03 | 2.72E-04 | 4.01E-03 | 1.22E+00 |
| cg13882285 |  |  | S_Shelf | 8.75E-01 | 8.97E-01 | 4.87E-02 | 2.73E-04 | 4.02E-03 | -1.02E+00 |
| cg20303886 | CYP4F3 | Body | OpenSea | 9.46E-01 | 9.61E-01 | 8.30E-03 | 2.77E-04 | 4.05E-03 | 2.18E+00 |
| cg07804024 | C7orf73 | TSS1500 | N_Shore | 2.43E-01 | 2.94E-01 | 1.12E-01 | 2.77E-04 | 4.05E-03 | 4.64E-01 |
| cg02002164 | CSRP3 | 1stExon;5'UTR | OpenSea | 9.77E-01 | 9.81E-01 | 3.49E-03 | 2.77E-04 | 4.05E-03 | 9.79E+00 |
| cg03101183 | C7orf50 | Body | OpenSea | 8.81E-01 | 9.00E-01 | 1.22E-02 | 2.81E-04 | 4.08E-03 | 9.61E-01 |
| cg08663866 | LOC100507389 | Body | OpenSea | 2.69E-01 | 2.30E-01 | -2.73E-02 | 2.84E-04 | 4.11E-03 | -3.03E+00 |
| cg06745030 | B3GNT7 | TSS200 | Island | 5.51E-02 | 7.03E-02 | 2.82E-02 | 2.89E-04 | 4.15E-03 | -1.47E-01 |
| cg15369645 | CDH4 | Body | OpenSea | 9.15E-01 | 9.03E-01 | -1.02E-02 | 2.89E-04 | 4.15E-03 | -4.75E+00 |
| cg16307473 |  |  | OpenSea | 1.22E-01 | 1.47E-01 | 2.47E-02 | 2.90E-04 | 4.16E-03 | -1.50E-01 |
| cg17150496 | CHCHD6 | Body | OpenSea | 8.38E-01 | 8.54E-01 | 2.59E-02 | 2.98E-04 | 4.22E-03 | -5.54E+00 |
| cg02996104 | FXYD6-FXYD2 | Body | OpenSea | 9.05E-01 | 9.32E-01 | 5.92E-02 | 2.98E-04 | 4.22E-03 | 2.09E+00 |
| cg05821961 | PGBD3;ERCC6 | 5'UTR;Body | OpenSea | 9.48E-01 | 9.57E-01 | 1.37E-02 | 2.98E-04 | 4.23E-03 | -2.38E+00 |
| cg07693880 |  |  | Island | 9.20E-01 | 9.33E-01 | 1.75E-02 | 2.99E-04 | 4.23E-03 | 6.36E+00 |
| cg02477714 |  |  | OpenSea | 9.43E-01 | 9.55E-01 | 2.05E-02 | 3.00E-04 | 4.25E-03 | -4.20E-01 |
| cg16692211 | ZNF408 | Body | Island | 9.88E-01 | 9.91E-01 | 1.63E-03 | 3.02E-04 | 4.26E-03 | 1.69E+01 |
| cg16776097 | PKP4;CCDC148 | TSS1500;Body;5'UTR | N_Shore | 5.51E-02 | 6.65E-02 | 2.63E-03 | 3.03E-04 | 4.27E-03 | 1.48E+00 |
| cg16879724 | CFAP97 | Body | OpenSea | 9.79E-01 | 9.76E-01 | -8.44E-03 | 3.04E-04 | 4.28E-03 | -2.07E+00 |
| cg08602892 | COL26A1 | Body | OpenSea | 9.50E-01 | 9.61E-01 | 1.16E-02 | 3.10E-04 | 4.33E-03 | 5.21E-01 |
| cg21592463 |  |  | OpenSea | 8.83E-01 | 8.60E-01 | -6.38E-02 | 3.12E-04 | 4.34E-03 | -5.27E-01 |
| cg03941368 | PLXNB2 | 5'UTR | S_Shore | 2.36E-01 | 2.20E-01 | -1.66E-02 | 3.12E-04 | 4.34E-03 | -1.11E+00 |
| cg03470623 |  |  | OpenSea | 6.83E-01 | 6.56E-01 | -4.56E-02 | 3.13E-04 | 4.35E-03 | -5.46E-02 |
| cg01643835 |  |  | OpenSea | 6.74E-01 | 6.27E-01 | -4.07E-02 | 3.13E-04 | 4.35E-03 | -8.18E-01 |
| cg25924139 | PTPRM | Body | OpenSea | 9.75E-01 | 9.79E-01 | 5.40E-03 | 3.14E-04 | 4.36E-03 | -8.46E+00 |
| cg12419678 | FUT11 | Body | S_Shore | 6.06E-02 | 7.00E-02 | 8.96E-03 | 3.15E-04 | 4.37E-03 | 8.66E-01 |
| cg11637980 | PGR | TSS200 | S_Shore | 7.44E-02 | 9.82E-02 | 3.75E-02 | 3.15E-04 | 4.37E-03 | -9.37E-01 |
| cg06733659 |  |  | S_Shelf | 1.47E-01 | 1.29E-01 | -9.74E-03 | 3.17E-04 | 4.38E-03 | -2.43E-01 |
| cg15549849 | GPR37 | Body | N_Shore | 9.75E-01 | 9.63E-01 | -1.51E-02 | 3.18E-04 | 4.40E-03 | -3.43E+00 |
| cg07550370 |  |  | OpenSea | 9.19E-01 | 9.30E-01 | 2.53E-02 | 3.18E-04 | 4.40E-03 | 3.49E+00 |
| cg13473803 |  |  | N_Shelf | 4.87E-01 | 5.30E-01 | 3.75E-02 | 3.20E-04 | 4.41E-03 | 1.75E+00 |
| cg23624713 |  |  | OpenSea | 9.79E-01 | 9.77E-01 | -3.80E-03 | 3.21E-04 | 4.42E-03 | -5.95E+00 |
| cg11711114 | NCKAP1 | TSS1500 | S_Shore | 2.37E-02 | 3.06E-02 | 1.05E-02 | 3.22E-04 | 4.42E-03 | 1.22E+01 |
| cg24066423 | PRMT3 | Body | OpenSea | 9.18E-01 | 8.99E-01 | -3.89E-02 | 3.23E-04 | 4.43E-03 | 4.40E-01 |
| cg15284964 | ATP2B2 | 5'UTR | OpenSea | 8.82E-01 | 8.99E-01 | 1.93E-02 | 3.28E-04 | 4.47E-03 | -3.48E+00 |
| cg02391432 | STX12 | TSS200 | N_Shore | 1.65E-02 | 1.86E-02 | 3.31E-03 | 3.29E-04 | 4.48E-03 | 2.52E+01 |
| cg12112406 | NXNL2 | Body | OpenSea | 8.75E-01 | 8.42E-01 | -5.02E-02 | 3.30E-04 | 4.49E-03 | 7.12E-01 |
| cg01933946 | C1orf109 | Body | N_Shore | 2.48E-02 | 2.30E-02 | -9.80E-04 | 3.30E-04 | 4.49E-03 | -5.24E+01 |
| cg24012572 | LOC101927437 | Body | OpenSea | 9.21E-01 | 9.42E-01 | 3.67E-02 | 3.34E-04 | 4.52E-03 | 3.62E+00 |
| cg02989600 | DDIT4L | TSS200 | S_Shore | 3.02E-02 | 2.73E-02 | -2.63E-03 | 3.38E-04 | 4.55E-03 | -3.75E+01 |
| cg00732327 | FER1L5 | Body | OpenSea | 9.82E-01 | 9.79E-01 | -3.14E-03 | 3.38E-04 | 4.55E-03 | -9.98E+00 |
| cg07838730 | SIGLEC15 | 3'UTR | S_Shelf | 9.10E-01 | 9.28E-01 | 2.62E-02 | 3.40E-04 | 4.57E-03 | -1.07E+00 |
| cg00702872 |  |  | OpenSea | 9.36E-01 | 9.47E-01 | 1.81E-02 | 3.52E-04 | 4.67E-03 | 2.86E+00 |
| cg08572565 | NIN | TSS1500 | S_Shore | 8.86E-02 | 1.14E-01 | 1.26E-02 | 3.54E-04 | 4.68E-03 | -1.03E-02 |
| cg05232009 |  |  | OpenSea | 9.55E-01 | 9.63E-01 | 9.85E-03 | 3.55E-04 | 4.69E-03 | 4.25E+00 |
| cg25838959 |  |  | OpenSea | 8.64E-01 | 8.98E-01 | 1.17E-02 | 3.55E-04 | 4.69E-03 | 1.05E+00 |
| cg24838345 | MTSS1 | Body | N_Shelf | 9.33E-01 | 9.18E-01 | -6.96E-03 | 3.55E-04 | 4.69E-03 | -4.12E+00 |
| cg16692653 |  |  | OpenSea | 9.30E-01 | 9.16E-01 | -2.46E-02 | 3.55E-04 | 4.69E-03 | -3.65E-01 |
| cg13233929 | CARKD | TSS200 | Island | 1.96E-02 | 2.38E-02 | 2.99E-03 | 3.58E-04 | 4.71E-03 | 1.11E-01 |
| cg03124146 |  |  | Island | 1.30E-01 | 1.79E-01 | 3.24E-02 | 3.62E-04 | 4.74E-03 | 2.66E+00 |
| cg07610074 | C3P1 | Body | OpenSea | 8.24E-01 | 8.55E-01 | 1.38E-02 | 3.66E-04 | 4.77E-03 | 1.31E-02 |
| cg22902534 | AP2M1 | 5'UTR | Island | 3.93E-02 | 4.56E-02 | 7.75E-03 | 3.68E-04 | 4.79E-03 | 1.41E+01 |
| cg18196334 |  |  | N_Shore | 6.51E-01 | 6.93E-01 | 5.62E-02 | 3.70E-04 | 4.81E-03 | 6.00E-01 |
| cg25274320 | ZNHIT2 | TSS1500 | S_Shore | 9.56E-01 | 9.48E-01 | -1.77E-02 | 3.70E-04 | 4.81E-03 | -9.02E+00 |
| cg16667192 |  |  | Island | 9.58E-01 | 9.64E-01 | 2.34E-03 | 3.72E-04 | 4.82E-03 | 7.17E-01 |
| cg20315722 | PLCD4 | Body | OpenSea | 9.06E-01 | 9.16E-01 | 1.00E-02 | 3.73E-04 | 4.83E-03 | 5.52E+00 |
| cg21071540 | MIR485;MIR453 | TSS200;TSS1500 | OpenSea | 9.58E-01 | 9.69E-01 | 1.78E-02 | 3.79E-04 | 4.87E-03 | 3.68E+00 |
| cg14690315 | MALAT1 | Body | S_Shelf | 4.22E-02 | 4.88E-02 | 1.25E-02 | 3.81E-04 | 4.89E-03 | 6.43E-01 |
| cg05530288 |  |  | OpenSea | 1.60E-02 | 1.76E-02 | 3.29E-03 | 3.82E-04 | 4.89E-03 | 3.45E+00 |
| cg00830087 | SPAG17 | TSS200 | Island | 1.90E-01 | 2.32E-01 | 5.27E-02 | 3.82E-04 | 4.90E-03 | -1.71E-01 |
| cg20973207 | H2AFY | TSS1500;5'UTR | S_Shore | 9.93E-02 | 1.20E-01 | 3.61E-02 | 3.84E-04 | 4.91E-03 | 8.13E-01 |
| cg20060470 | PARP3 |  | S_Shelf | 8.68E-01 | 8.81E-01 | 1.49E-02 | 3.84E-04 | 4.91E-03 | -5.63E-03 |
| cg11177955 | C1orf58;AIDA | 5'UTR;TSS1500 | S_Shore | 1.60E-02 | 1.81E-02 | 1.88E-03 | 3.88E-04 | 4.94E-03 | 2.92E+00 |
| cg05537796 | MIR369;MIR410;MIR412;MIR656 | TSS200;TSS1500;Body | Island | 8.75E-01 | 8.96E-01 | 2.97E-02 | 3.91E-04 | 4.96E-03 | 9.68E-02 |
| cg10191248 | INPP5A | Body | OpenSea | 9.13E-01 | 9.32E-01 | 6.28E-03 | 3.97E-04 | 5.01E-03 | 1.43E+00 |
| cg06837911 |  |  | OpenSea | 9.59E-01 | 9.49E-01 | -5.22E-03 | 4.03E-04 | 5.05E-03 | -2.18E+00 |
| cg17090245 | STARD10 | 1stExon;5'UTR | Island | 1.70E-02 | 1.86E-02 | 2.27E-03 | 4.04E-04 | 5.06E-03 | 5.58E+01 |
| cg22851879 |  |  | OpenSea | 9.61E-01 | 9.70E-01 | 7.97E-03 | 4.06E-04 | 5.07E-03 | 4.47E+00 |
| cg03988131 | LDLRAD4 | Body | OpenSea | 9.36E-01 | 9.54E-01 | 3.31E-02 | 4.06E-04 | 5.07E-03 | 7.87E-01 |
| cg01258499 | PDRG1 | TSS200 | S_Shore | 1.51E-02 | 1.73E-02 | 3.09E-03 | 4.09E-04 | 5.09E-03 | 3.94E-01 |
| cg14546350 | TMEM38A;SMIM7 | Body;TSS1500;ExonBnd | Island | 1.07E-02 | 1.28E-02 | 1.26E-03 | 4.11E-04 | 5.11E-03 | 4.35E+00 |
| cg03903974 | FAM49B | Body | OpenSea | 9.43E-01 | 9.27E-01 | -1.27E-02 | 4.12E-04 | 5.11E-03 | -3.30E+00 |
| cg12709669 |  |  | N_Shore | 2.60E-01 | 2.35E-01 | -4.51E-02 | 4.13E-04 | 5.12E-03 | -2.23E-01 |
| cg17777559 | OTOG | Body | OpenSea | 9.56E-01 | 9.75E-01 | 3.08E-02 | 4.13E-04 | 5.12E-03 | 2.07E+00 |
| cg26776035 |  |  | OpenSea | 1.23E-01 | 1.46E-01 | 3.70E-02 | 4.17E-04 | 5.15E-03 | -9.51E-01 |
| cg25812921 | LOC339975 | Body | OpenSea | 7.00E-01 | 7.35E-01 | 4.78E-02 | 4.17E-04 | 5.15E-03 | 8.23E-01 |
| cg24769830 | HHLA2 | 5'UTR | OpenSea | 8.02E-01 | 8.47E-01 | 2.43E-02 | 4.17E-04 | 5.15E-03 | 5.31E-01 |
| cg14763874 | WFDC12 | TSS200 | OpenSea | 9.54E-01 | 9.64E-01 | 7.87E-03 | 4.20E-04 | 5.17E-03 | 4.70E+00 |
| cg06453915 | TAF6L | TSS1500 | N_Shore | 4.40E-02 | 5.09E-02 | 7.96E-03 | 4.22E-04 | 5.19E-03 | 1.85E+00 |
| cg01754828 | ADGRB3;LOC101928307 | TSS1500;TSS200 | N_Shore | 9.11E-02 | 1.15E-01 | 2.80E-02 | 4.24E-04 | 5.20E-03 | -2.74E+00 |
| cg16513734 | KHDRBS3 | Body | OpenSea | 1.19E-01 | 1.47E-01 | 2.80E-02 | 4.25E-04 | 5.20E-03 | -1.01E-03 |
| cg08180934 | STRAP | TSS200 | Island | 1.76E-02 | 1.94E-02 | 2.14E-03 | 4.25E-04 | 5.21E-03 | 2.03E+01 |
| cg00128530 | RSL24D1 | TSS200 | S_Shore | 1.99E-02 | 2.17E-02 | 3.19E-03 | 4.27E-04 | 5.22E-03 | 4.28E+01 |
| cg21772826 | TPRG1 | 3'UTR | OpenSea | 3.65E-01 | 4.06E-01 | 9.41E-02 | 4.27E-04 | 5.22E-03 | 3.52E-01 |
| cg24786495 | ACSL6 | Body | OpenSea | 9.48E-01 | 9.59E-01 | 1.79E-02 | 4.30E-04 | 5.24E-03 | -3.27E+00 |
| cg20944662 |  |  | OpenSea | 9.67E-01 | 9.76E-01 | 7.42E-03 | 4.35E-04 | 5.28E-03 | 1.72E+00 |
| cg04496042 |  |  | N_Shore | 8.40E-01 | 8.70E-01 | -7.76E-04 | 4.36E-04 | 5.28E-03 | 3.00E+00 |
| cg15653779 |  |  | OpenSea | 8.63E-01 | 8.73E-01 | 1.10E-02 | 4.37E-04 | 5.29E-03 | 1.78E+00 |
| cg09235303 |  |  | OpenSea | 5.93E-01 | 6.22E-01 | 1.83E-02 | 4.38E-04 | 5.30E-03 | 1.18E+00 |
| cg13467135 | KIAA1598;KIAA1598 | Body | OpenSea | 9.47E-01 | 9.36E-01 | -1.53E-02 | 4.40E-04 | 5.31E-03 | 9.20E-04 |
| cg08990121 | RPGR;RPGR | Body | OpenSea | 6.80E-01 | 7.11E-01 | 3.74E-02 | 4.43E-04 | 5.33E-03 | 4.84E-01 |
| cg06419113 |  |  | OpenSea | 8.57E-01 | 8.79E-01 | 3.73E-02 | 4.44E-04 | 5.34E-03 | -1.30E+00 |
| cg19629208 | SLC34A1;SLC34A1 | TSS1500;TSS1500 | OpenSea | 9.49E-01 | 9.36E-01 | -3.66E-02 | 4.45E-04 | 5.35E-03 | -1.25E+00 |
| cg17747905 |  |  | OpenSea | 8.68E-01 | 8.81E-01 | 2.55E-02 | 4.45E-04 | 5.35E-03 | -5.28E-01 |
| cg12198839 | NCK2 | Body | OpenSea | 9.74E-01 | 9.66E-01 | 2.97E-03 | 4.47E-04 | 5.36E-03 | -8.24E-01 |
| cg25409748 | RAB7L1 | TSS200;TSS200;TSS200;TSS200 | S_Shore | 1.90E-02 | 2.11E-02 | 3.17E-03 | 4.47E-04 | 5.36E-03 | 6.01E+01 |
| cg21261477 | CABC1 | TSS1500 | Island | 3.89E-02 | 3.58E-02 | -9.73E-04 | 4.53E-04 | 5.40E-03 | -3.27E-01 |
| cg26321437 | RAB27A | TSS1500;TSS1500;5'UTR | OpenSea | 9.64E-01 | 9.59E-01 | -4.44E-03 | 4.54E-04 | 5.41E-03 | -3.81E-01 |
| cg09839978 | VIPR2 | TSS200;Body;Body;Body | S_Shelf | 8.38E-01 | 8.65E-01 | 2.56E-02 | 4.55E-04 | 5.41E-03 | 1.41E+00 |
| cg10862468 | CYP2E1 | Body | Island | 1.92E-01 | 2.78E-01 | 4.49E-02 | 4.55E-04 | 5.42E-03 | 6.43E-01 |
| cg05342157 | TMEM63C | 5'UTR | OpenSea | 9.76E-01 | 9.71E-01 | -7.45E-03 | 4.58E-04 | 5.44E-03 | 3.69E+00 |
| cg09398502 |  |  | OpenSea | 1.71E-01 | 1.29E-01 | -8.86E-02 | 4.58E-04 | 5.44E-03 | -1.63E-01 |
| cg26897524 |  |  | OpenSea | 8.98E-01 | 8.78E-01 | -2.03E-02 | 4.63E-04 | 5.47E-03 | 1.06E+00 |
| cg08323766 |  |  | N_Shore | 9.66E-01 | 9.59E-01 | -1.06E-02 | 4.63E-04 | 5.47E-03 | 3.21E-01 |
| cg05491695 | MALAT1 | Body | S_Shore | 1.66E-01 | 1.28E-01 | -8.75E-02 | 4.66E-04 | 5.49E-03 | -1.06E+00 |
| cg14634738 | RBM5 | TSS1500 | N_Shore | 1.34E-01 | 1.11E-01 | -2.34E-03 | 4.67E-04 | 5.49E-03 | -5.06E+00 |
| cg25541397 | KCNK10 | 1stExon;5'UTR;Body;Body | OpenSea | 8.21E-01 | 8.70E-01 | 1.03E-01 | 4.69E-04 | 5.51E-03 | 4.45E-01 |
| cg23180489 | LYNX1 | TSS200;TSS1500;TSS200;TSS1500 | S_Shore | 7.97E-01 | 7.42E-01 | -1.28E-01 | 4.69E-04 | 5.51E-03 | -7.01E-01 |
| cg09434586 | XPO4 | Body | N_Shore | 3.58E-02 | 4.22E-02 | 7.63E-03 | 4.75E-04 | 5.55E-03 | -6.37E-01 |
| cg23144473 |  |  | OpenSea | 1.76E-01 | 1.99E-01 | 1.99E-02 | 4.78E-04 | 5.57E-03 | -2.09E-01 |
| cg08805208 | GPR133 | Body | OpenSea | 8.15E-01 | 8.33E-01 | 2.38E-02 | 4.79E-04 | 5.58E-03 | -1.40E+00 |
| cg10377764 | RAD52 | TSS1500 | Island | 2.32E-02 | 2.13E-02 | -2.01E-03 | 4.81E-04 | 5.59E-03 | -1.38E+01 |
| cg07346603 | C5orf58 | TSS200 | N_Shore | 9.88E-01 | 9.90E-01 | 4.62E-03 | 4.83E-04 | 5.60E-03 | 9.04E+00 |
| cg01034367 | ABCC11 | Body | OpenSea | 9.50E-01 | 9.40E-01 | -7.67E-03 | 4.84E-04 | 5.61E-03 | -5.21E-01 |
| cg14188929 |  |  | OpenSea | 9.71E-01 | 9.78E-01 | 1.99E-02 | 4.84E-04 | 5.61E-03 | 1.53E+00 |
| cg22510460 | EVA1A | TSS1500;5'UTR | S_Shore | 5.96E-01 | 6.24E-01 | 2.60E-02 | 4.84E-04 | 5.62E-03 | 1.16E-01 |
| cg07616394 | HHEX | Body | S_Shore | 5.35E-02 | 6.68E-02 | 1.16E-02 | 4.85E-04 | 5.62E-03 | -7.99E-02 |
| cg05937941 | FKBP9 | TSS1500 | N_Shore | 8.94E-01 | 9.23E-01 | 1.08E-02 | 4.85E-04 | 5.62E-03 | 2.44E+00 |
| cg18537923 | LOC101929754;ZBTB20 | TSS1500;Body;5'UTR | OpenSea | 9.24E-01 | 9.11E-01 | -2.39E-02 | 4.92E-04 | 5.67E-03 | 5.00E-03 |
| cg25140002 |  |  | Island | 9.71E-01 | 9.76E-01 | 4.93E-03 | 4.94E-04 | 5.69E-03 | 5.37E+00 |
| cg24277994 |  |  | OpenSea | 5.94E-01 | 6.44E-01 | 2.83E-02 | 4.99E-04 | 5.72E-03 | 4.94E-01 |
| cg13959174 | LINC01399 | Body | OpenSea | 1.63E-01 | 1.46E-01 | -1.63E-02 | 5.08E-04 | 5.78E-03 | -1.45E+00 |
| cg24681175 |  |  | OpenSea | 1.65E-01 | 1.99E-01 | 7.57E-02 | 5.08E-04 | 5.78E-03 | -5.30E-02 |
| cg23198826 | PPP1R1B | TSS1500 | N_Shore | 5.85E-02 | 7.04E-02 | -3.05E-04 | 5.10E-04 | 5.79E-03 | 4.26E+00 |
| cg23044891 | MAPT | 5'UTR | OpenSea | 8.48E-01 | 8.96E-01 | 9.99E-02 | 5.11E-04 | 5.80E-03 | 4.81E-01 |
| cg05201403 | STK32C | Body | N_Shelf | 5.50E-01 | 5.93E-01 | 3.38E-02 | 5.14E-04 | 5.82E-03 | 6.98E-01 |
| cg21712831 |  |  | OpenSea | 4.23E-02 | 5.87E-02 | 2.29E-02 | 5.16E-04 | 5.83E-03 | -6.12E-02 |
| cg20140052 |  |  | Island | 2.50E-02 | 2.92E-02 | 9.13E-04 | 5.17E-04 | 5.84E-03 | 6.99E-01 |
| cg20754324 | SOX10 | TSS1500 | S_Shore | 7.87E-01 | 8.03E-01 | 2.91E-02 | 5.25E-04 | 5.89E-03 | -7.76E-02 |
| cg04658591 | MXI1 | Body;5'UTR | S_Shore | 1.58E-02 | 1.77E-02 | 1.80E-03 | 5.27E-04 | 5.91E-03 | 2.46E-01 |
| cg01877391 | METTL9 | TSS1500 | N_Shore | 4.82E-02 | 5.40E-02 | 3.15E-03 | 5.30E-04 | 5.92E-03 | 4.87E-01 |
| cg17546454 | ZFX | Body | OpenSea | 8.07E-01 | 7.64E-01 | -2.96E-02 | 5.36E-04 | 5.97E-03 | 5.70E-02 |
| cg09317752 | MTHFSD | Body | OpenSea | 9.85E-01 | 9.87E-01 | 2.32E-03 | 5.36E-04 | 5.97E-03 | 1.63E+01 |
| cg18167921 | DEPDC1 | TSS1500 | S_Shore | 1.46E-01 | 1.74E-01 | 3.16E-02 | 5.37E-04 | 5.97E-03 | -3.20E-02 |
| cg07123796 |  |  | S_Shore | 4.96E-01 | 5.12E-01 | 1.76E-02 | 5.39E-04 | 5.98E-03 | 1.37E-02 |
| cg25964728 | TMEM22 | 5'UTR;5'UTR;5'UTR | S_Shore | 9.40E-02 | 1.20E-01 | 4.49E-02 | 5.39E-04 | 5.98E-03 | -6.56E-02 |
| cg24169711 |  |  | N_Shore | 9.52E-01 | 9.66E-01 | 2.78E-02 | 5.45E-04 | 6.02E-03 | 3.00E-01 |
| cg08225417 | HIPK1-AS1; | Body;TSS1500 | N_Shore | 1.48E-02 | 1.65E-02 | -1.02E-05 | 5.52E-04 | 6.07E-03 | 1.60E+01 |
| cg00321709 | CYP2E1 | Body | Island | 1.93E-01 | 2.91E-01 | 4.32E-02 | 5.55E-04 | 6.09E-03 | 5.44E-01 |
| cg17735531 | HCN2 | Body | Island | 5.38E-01 | 5.09E-01 | -4.38E-02 | 5.56E-04 | 6.10E-03 | -1.14E+00 |
| cg03002086 | NEURL1-AS1;NEURL1 | Body | OpenSea | 8.79E-01 | 8.93E-01 | 1.98E-02 | 5.59E-04 | 6.11E-03 | -1.90E+00 |
| cg17683386 |  |  | OpenSea | 2.43E-01 | 2.71E-01 | 1.47E-02 | 5.63E-04 | 6.14E-03 | 7.13E-01 |
| cg24152000 | KIF26A | Body | N_Shore | 8.71E-01 | 8.58E-01 | 7.91E-03 | 5.65E-04 | 6.15E-03 | -8.66E-01 |
| cg16485952 | TNXB | Body | OpenSea | 9.67E-01 | 9.73E-01 | 5.56E-03 | 5.71E-04 | 6.19E-03 | 6.83E+00 |
| cg08297626 | ATP11A | Body | N_Shelf | 9.50E-01 | 9.61E-01 | 2.16E-02 | 5.72E-04 | 6.21E-03 | 2.27E+00 |
| cg25273233 | PRDM13 | TSS200 | N_Shore | 9.76E-02 | 1.23E-01 | 9.06E-03 | 5.74E-04 | 6.22E-03 | 2.79E+00 |
| cg04435763 | IQSEC1 | Body | N_Shelf | 9.64E-01 | 9.72E-01 | 1.50E-02 | 5.76E-04 | 6.22E-03 | -1.17E+01 |
| cg09670189 | RAET1G | Body | Island | 2.77E-02 | 3.10E-02 | 3.22E-03 | 5.78E-04 | 6.24E-03 | 1.93E+01 |
| cg23616707 | PPP2R2C | 5'UTR | OpenSea | 9.84E-01 | 9.88E-01 | 1.34E-03 | 5.78E-04 | 6.24E-03 | 2.50E+01 |
| cg05142520 | WNT10B | 5'UTR | Island | 3.97E-01 | 4.23E-01 | 2.39E-02 | 5.80E-04 | 6.25E-03 | 3.66E-01 |
| cg06181187 | CRYGB | TSS200 | OpenSea | 8.80E-01 | 9.01E-01 | 9.63E-03 | 5.80E-04 | 6.25E-03 | 5.99E-01 |
| cg17020398 |  |  | OpenSea | 9.75E-01 | 9.80E-01 | 3.49E-03 | 5.81E-04 | 6.26E-03 | 6.93E+00 |
| cg09157285 |  |  | S_Shelf | 8.66E-01 | 8.35E-01 | -3.11E-02 | 5.81E-04 | 6.26E-03 | 9.39E-01 |
| cg25578781 | ETS1 | 5'UTR | OpenSea | 1.02E-01 | 1.27E-01 | 1.82E-02 | 5.83E-04 | 6.27E-03 | -2.78E-02 |
| cg07194301 | PRCC | ExonBnd;Body | OpenSea | 9.81E-01 | 9.86E-01 | 4.64E-03 | 5.84E-04 | 6.28E-03 | 9.78E+00 |
| cg00228712 | FBXW11 | 3'UTR | OpenSea | 7.01E-01 | 7.31E-01 | 9.21E-02 | 5.93E-04 | 6.33E-03 | -5.10E-01 |
| cg26940725 | RPL21;RPL21P28 | 5'UTR;Body | Island | 1.44E-02 | 1.66E-02 | 2.97E-03 | 5.95E-04 | 6.35E-03 | 2.19E+00 |
| cg16493922 | ZBED3-AS1 | Body | OpenSea | 9.14E-01 | 8.89E-01 | -3.36E-02 | 5.97E-04 | 6.36E-03 | 4.64E-02 |
| cg24829888 | ARHGAP15 | Body | OpenSea | 8.69E-01 | 8.87E-01 | 2.80E-02 | 5.97E-04 | 6.36E-03 | -1.24E-01 |
| cg19889971 |  |  | N_Shore | 9.40E-01 | 9.51E-01 | 2.65E-02 | 5.97E-04 | 6.36E-03 | -1.74E+00 |
| cg01266688 | ACTR1B | Body | OpenSea | 9.85E-01 | 9.88E-01 | 2.44E-03 | 5.99E-04 | 6.37E-03 | 3.88E+00 |
| cg03244036 | HIC1 | TSS1500 | Island | 3.25E-02 | 2.82E-02 | -9.24E-04 | 6.00E-04 | 6.38E-03 | -2.57E+01 |
| cg24044884 | NSG1 | TSS1500 | N_Shore | 3.50E-01 | 4.27E-01 | 6.38E-02 | 6.04E-04 | 6.40E-03 | 6.07E-01 |
| cg25593631 |  |  | S_Shore | 7.06E-01 | 6.85E-01 | -2.12E-02 | 6.05E-04 | 6.41E-03 | -2.05E+00 |
| cg06835647 |  |  | OpenSea | 9.36E-01 | 9.24E-01 | -1.90E-02 | 6.06E-04 | 6.41E-03 | 8.50E-02 |
| cg27322781 | KIF3C | Body | OpenSea | 9.48E-01 | 9.42E-01 | -9.98E-03 | 6.07E-04 | 6.42E-03 | -1.11E+00 |
| cg18006085 | LRRC59 | TSS200 | Island | 2.18E-02 | 2.32E-02 | 1.76E-03 | 6.10E-04 | 6.44E-03 | 8.98E+00 |
| cg02163400 | NXN | TSS200 | Island | 2.62E-02 | 3.04E-02 | 5.41E-03 | 6.12E-04 | 6.45E-03 | 2.17E+00 |
| cg07728254 | CYP3A43 | TSS1500 | OpenSea | 9.29E-01 | 8.99E-01 | -1.69E-02 | 6.13E-04 | 6.45E-03 | -3.37E+00 |
| cg03199508 | CD96 | Body | OpenSea | 8.98E-01 | 8.85E-01 | -1.96E-02 | 6.15E-04 | 6.46E-03 | 1.16E+00 |
| cg23729340 |  |  | OpenSea | 6.68E-01 | 6.92E-01 | 3.71E-02 | 6.20E-04 | 6.49E-03 | -1.07E+00 |
| cg10711871 | WNT7A | Body | Island | 7.59E-01 | 7.88E-01 | 5.30E-02 | 6.21E-04 | 6.50E-03 | 3.92E-01 |
| cg03043824 |  |  | OpenSea | 9.76E-01 | 9.80E-01 | 2.18E-03 | 6.22E-04 | 6.50E-03 | 2.25E+01 |
| cg18389023 |  |  | N_Shore | 9.00E-01 | 9.22E-01 | 1.16E-02 | 6.27E-04 | 6.53E-03 | 1.26E+00 |
| cg00694357 | ZBTB7B | 5'UTR | S_Shore | 2.28E-02 | 2.60E-02 | 4.75E-03 | 6.28E-04 | 6.54E-03 | 2.34E+01 |
| cg07814664 |  |  | OpenSea | 9.45E-01 | 9.32E-01 | -1.18E-02 | 6.29E-04 | 6.54E-03 | -3.93E+00 |
| cg14594704 |  |  | OpenSea | 9.33E-01 | 9.20E-01 | -1.92E-02 | 6.30E-04 | 6.55E-03 | -4.70E+00 |
| cg15437444 | FBXL2 | Body | S_Shore | 2.48E-01 | 2.68E-01 | 1.85E-02 | 6.33E-04 | 6.56E-03 | 1.26E-01 |
| cg19564630 | BBS7 | TSS1500 | S_Shore | 3.64E-02 | 4.63E-02 | 1.49E-02 | 6.38E-04 | 6.60E-03 | -1.12E-01 |
| cg01472463 | SH3PXD2A | Body | S_Shore | 2.71E-02 | 2.34E-02 | -9.46E-04 | 6.41E-04 | 6.61E-03 | -2.32E+00 |
| cg01335986 |  |  | OpenSea | 9.26E-01 | 9.07E-01 | -2.07E-02 | 6.41E-04 | 6.61E-03 | 6.13E-01 |
| cg00238698 | PBX3 | Body | OpenSea | 9.30E-01 | 9.37E-01 | 1.33E-02 | 6.42E-04 | 6.61E-03 | 5.99E-01 |
| cg04063860 |  |  | OpenSea | 4.50E-02 | 5.43E-02 | 1.05E-02 | 6.43E-04 | 6.62E-03 | -5.16E-01 |
| cg25199878 | NCKAP1;NCKAP1 | TSS1500;TSS1500 | S_Shore | 2.17E-02 | 2.68E-02 | 7.56E-03 | 6.45E-04 | 6.63E-03 | 7.25E+00 |
| cg03864121 | CTDP1;CTDP1 | Body | Island | 9.57E-01 | 9.48E-01 | -1.28E-02 | 6.50E-04 | 6.66E-03 | -5.26E+00 |
| cg13743428 | PCSK6 | Body | OpenSea | 9.28E-01 | 9.42E-01 | 1.03E-02 | 6.50E-04 | 6.67E-03 | 8.69E-01 |
| cg04965141 | SFRP2 | TSS1500 | Island | 1.05E-01 | 1.29E-01 | 3.59E-02 | 6.53E-04 | 6.68E-03 | -1.89E+00 |
| cg11571001 | GTF2H4;VARS2 | 3'UTR;TSS1500;TSS200 | Island | 4.70E-02 | 5.61E-02 | 3.43E-03 | 6.53E-04 | 6.68E-03 | 1.07E+01 |
| cg11148957 | CAMTA1 | Body | OpenSea | 8.60E-01 | 8.76E-01 | 2.56E-02 | 6.55E-04 | 6.69E-03 | -7.79E-02 |
| cg00931339 | NAT14;ZNF628 | TSS1500;Body | Island | 9.49E-01 | 9.58E-01 | 5.07E-03 | 6.56E-04 | 6.70E-03 | 4.17E-01 |
| cg17146095 |  |  | S_Shore | 8.92E-01 | 9.11E-01 | 3.79E-02 | 6.63E-04 | 6.74E-03 | -2.00E-01 |
| cg08358683 | NNT;NNT | TSS1500 | N_Shore | 1.12E-01 | 1.33E-01 | 3.28E-02 | 6.68E-04 | 6.77E-03 | -1.28E+00 |
| cg00477203 | DUSP27 | Body | OpenSea | 9.64E-01 | 9.73E-01 | 3.73E-03 | 6.69E-04 | 6.77E-03 | 1.68E+00 |
| cg20069939 |  |  | OpenSea | 9.62E-01 | 9.57E-01 | -8.36E-03 | 6.79E-04 | 6.83E-03 | -2.58E+00 |
| cg24956739 |  |  | OpenSea | 9.28E-01 | 9.42E-01 | 2.25E-02 | 6.79E-04 | 6.83E-03 | -8.83E-01 |
| cg26862834 |  |  | OpenSea | 9.42E-01 | 9.54E-01 | 2.93E-02 | 6.88E-04 | 6.88E-03 | 4.24E-01 |
| cg24418664 | ERG | 5'UTR;Body | OpenSea | 8.78E-01 | 8.95E-01 | 2.20E-02 | 6.90E-04 | 6.90E-03 | -1.88E+00 |
| cg10525161 | TKT | Body | OpenSea | 9.48E-01 | 9.55E-01 | 1.38E-02 | 6.90E-04 | 6.90E-03 | -9.46E+00 |
| cg01412450 | NCRNA00119;NPHP3 | Body;TSS200 | S_Shore | 5.53E-02 | 6.36E-02 | 3.82E-03 | 6.91E-04 | 6.90E-03 | 2.55E+00 |
| cg16459480 | DIS3L2 | Body | OpenSea | 9.49E-01 | 9.58E-01 | 2.03E-02 | 6.93E-04 | 6.91E-03 | -5.50E+00 |
| cg08699623 | GPRIN3 | 5'UTR | OpenSea | 9.28E-01 | 9.09E-01 | -3.34E-02 | 6.95E-04 | 6.93E-03 | -6.79E+00 |
| cg05460950 | FOXK1 | Body | Island | 9.14E-01 | 9.04E-01 | -5.51E-03 | 6.98E-04 | 6.94E-03 | -6.82E+00 |
| cg06885284 | SLC18A1 | Body | OpenSea | 9.77E-01 | 9.82E-01 | 5.83E-03 | 7.02E-04 | 6.97E-03 | 1.33E+01 |
| cg00919689 | MAD1L1 | Body | OpenSea | 9.68E-01 | 9.77E-01 | 1.92E-02 | 7.05E-04 | 6.98E-03 | 1.16E-02 |
| cg21482312 | SEMA5B; | Body | OpenSea | 9.13E-01 | 9.25E-01 | 1.25E-02 | 7.07E-04 | 6.99E-03 | -6.27E-01 |
| cg10133282 |  |  | OpenSea | 8.10E-01 | 8.28E-01 | 1.12E-02 | 7.10E-04 | 7.01E-03 | 1.74E-01 |
| cg26962057 |  |  | Island | 1.23E-02 | 1.11E-02 | -2.11E-03 | 7.10E-04 | 7.02E-03 | -5.57E+00 |
| cg15945863 | SARNP | Body | OpenSea | 9.84E-01 | 9.86E-01 | -9.63E-04 | 7.10E-04 | 7.02E-03 | 2.57E+01 |
| cg23060965 | POLB | Body | OpenSea | 8.96E-01 | 8.84E-01 | -1.16E-02 | 7.11E-04 | 7.02E-03 | -1.31E+00 |
| cg24202772 | DMBT1 | Body | Island | 9.54E-01 | 9.64E-01 | 1.13E-02 | 7.13E-04 | 7.04E-03 | 2.78E+00 |
| cg25991744 | ABCC1 | Body | OpenSea | 9.46E-01 | 9.58E-01 | 2.25E-02 | 7.15E-04 | 7.04E-03 | -3.57E+00 |
| cg17936901 | ARHGAP19;ARHGAP19-SLIT1 | 3'UTR;Body | OpenSea | 7.49E-01 | 7.86E-01 | 7.37E-02 | 7.17E-04 | 7.05E-03 | -1.68E-01 |
| cg01969129 |  |  | OpenSea | 9.37E-01 | 9.25E-01 | -1.64E-02 | 7.18E-04 | 7.06E-03 | 1.22E+00 |
| cg03794372 | NXPE3 | 5'UTR | OpenSea | 9.88E-01 | 9.84E-01 | -1.41E-02 | 7.20E-04 | 7.07E-03 | -1.36E+00 |
| cg22511947 | FN1 | TSS1500 | S_Shore | 7.10E-02 | 8.80E-02 | 2.51E-02 | 7.22E-04 | 7.09E-03 | -3.62E-02 |
| cg08820191 | AP3B1 | TSS1500 | S_Shore | 3.16E-02 | 3.59E-02 | 7.96E-03 | 7.23E-04 | 7.09E-03 | -1.09E+01 |
| cg16662106 | EFCAB6 | Body | OpenSea | 8.03E-01 | 7.84E-01 | -4.24E-03 | 7.24E-04 | 7.10E-03 | -5.38E-02 |
| cg02857048 | GADD45G | TSS1500 | S_Shore | 1.40E-02 | 1.59E-02 | 2.18E-03 | 7.29E-04 | 7.13E-03 | 6.05E+00 |
| cg10397419 | PRKCE | Body | OpenSea | 9.09E-01 | 9.31E-01 | 4.24E-02 | 7.33E-04 | 7.15E-03 | 3.48E+00 |
| cg07819357 | ARNTL | 5'UTR | OpenSea | 9.69E-01 | 9.73E-01 | 2.89E-03 | 7.35E-04 | 7.16E-03 | 4.99E+00 |
| cg06183287 | SLC5A9;SLC5A9 | TSS1500 | OpenSea | 9.73E-01 | 9.68E-01 | -7.49E-03 | 7.36E-04 | 7.17E-03 | 1.99E+00 |
| cg19472909 | TMEM175 | Body | N_Shore | 9.78E-01 | 9.82E-01 | -1.45E-03 | 7.37E-04 | 7.17E-03 | 6.80E+00 |
| cg13651986 | WIPF1 | TSS1500 | S_Shore | 1.48E-01 | 1.72E-01 | 3.59E-02 | 7.38E-04 | 7.18E-03 | -1.45E+00 |
| cg06352370 | XKR8 | TSS200 | Island | 1.81E-02 | 1.93E-02 | 1.17E-03 | 7.39E-04 | 7.18E-03 | 6.31E+00 |
| cg11870153 | LOC100289495;SFT2D1 | Body;TSS1500 | S_Shore | 2.69E-02 | 3.25E-02 | 1.03E-02 | 7.40E-04 | 7.19E-03 | -4.02E+00 |
| cg04740057 |  |  | Island | 3.09E-02 | 3.57E-02 | 4.77E-03 | 7.43E-04 | 7.20E-03 | 5.60E+00 |
| cg17578917 | MROH2B;MROH2B | ExonBnd;Body | OpenSea | 9.74E-01 | 9.70E-01 | -3.66E-03 | 7.45E-04 | 7.21E-03 | -7.62E+00 |
| cg21697897 | LINC00242;LINC00574 | Body | Island | 2.53E-02 | 2.97E-02 | 6.09E-03 | 7.47E-04 | 7.23E-03 | -5.20E+00 |
| cg22603436 | PELI1 | 5'UTR | N_Shore | 2.07E-01 | 1.78E-01 | -5.96E-02 | 7.49E-04 | 7.24E-03 | -3.91E+00 |
| cg12746652 | KIAA1549 | Body | Island | 9.67E-01 | 9.73E-01 | 5.90E-03 | 7.54E-04 | 7.27E-03 | 1.88E+00 |
| cg18551822 | GMDS | Body | OpenSea | 9.01E-01 | 8.78E-01 | -3.53E-02 | 7.57E-04 | 7.28E-03 | 2.30E-01 |
| cg00357532 | NCKAP1;NCKAP1 | TSS1500 | S_Shore | 1.65E-02 | 2.10E-02 | 7.23E-03 | 7.58E-04 | 7.29E-03 | 8.93E-01 |
| cg22193559 |  |  | N_Shore | 3.01E-02 | 4.32E-02 | 2.07E-02 | 7.64E-04 | 7.33E-03 | 9.00E-01 |
| cg05438653 | MFSD9 | Body | OpenSea | 9.86E-01 | 9.88E-01 | 4.79E-04 | 7.65E-04 | 7.33E-03 | 4.30E+01 |
| cg25195301 |  |  | OpenSea | 9.61E-01 | 9.68E-01 | -3.08E-03 | 7.65E-04 | 7.33E-03 | 3.44E+00 |
| cg25183989 | SYCP2 | TSS1500 | Island | 3.91E-01 | 4.24E-01 | 3.66E-02 | 7.72E-04 | 7.37E-03 | 3.45E+00 |
| cg07929125 | PLXDC1 | Body | OpenSea | 8.32E-01 | 8.48E-01 | 1.79E-02 | 7.75E-04 | 7.39E-03 | -8.18E-02 |
| cg11488878 | TSHZ2 | TSS200 | N_Shore | 1.08E-01 | 1.42E-01 | 3.10E-02 | 7.76E-04 | 7.40E-03 | -1.29E-01 |
| cg01340229 |  |  | OpenSea | 8.49E-01 | 8.19E-01 | -2.89E-02 | 7.77E-04 | 7.41E-03 | 3.03E+00 |
| cg14996023 |  |  | N_Shore | 5.66E-01 | 5.36E-01 | -6.22E-02 | 7.79E-04 | 7.41E-03 | -1.93E-01 |
| cg11747586 | NRCAM | TSS1500;Body | OpenSea | 9.69E-01 | 9.63E-01 | -1.73E-02 | 7.83E-04 | 7.43E-03 | -3.82E+00 |
| cg13529269 | PPA2 | TSS200 | S_Shore | 1.73E-02 | 2.05E-02 | 3.78E-03 | 7.87E-04 | 7.45E-03 | -5.84E-01 |
| cg06291428 | CTNND1 | 5'UTR;Body | OpenSea | 9.71E-01 | 9.58E-01 | -2.67E-02 | 7.91E-04 | 7.48E-03 | -9.89E-01 |
| cg16672203 | C6orf27 | Body | OpenSea | 4.47E-01 | 4.15E-01 | -5.03E-02 | 7.95E-04 | 7.50E-03 | -6.36E+00 |
| cg00573202 |  |  | OpenSea | 8.04E-01 | 8.28E-01 | 2.93E-02 | 7.95E-04 | 7.50E-03 | -2.88E-01 |
| cg24948792 | SFXN3;PDZD7 | TSS1500;Body | Island | 9.25E-01 | 9.41E-01 | 9.30E-03 | 7.96E-04 | 7.50E-03 | 3.44E+00 |
| cg11956560 |  |  | OpenSea | 9.60E-01 | 9.52E-01 | -8.58E-03 | 8.05E-04 | 7.55E-03 | 1.27E+00 |
| cg06768203 | ENPP4 | TSS200 | Island | 2.49E-02 | 2.20E-02 | -4.49E-03 | 8.08E-04 | 7.57E-03 | -1.63E+01 |
| cg11599622 | LARP4B | Body | OpenSea | 9.78E-01 | 9.82E-01 | 3.38E-03 | 8.09E-04 | 7.57E-03 | 1.94E+00 |
| cg23858558 | SDC2 | TSS1500 | N_Shore | 7.06E-02 | 8.62E-02 | 2.28E-02 | 8.16E-04 | 7.61E-03 | -2.28E+00 |
| cg09171428 | PARVG | Body | OpenSea | 9.63E-01 | 9.70E-01 | 1.35E-02 | 8.17E-04 | 7.62E-03 | -8.10E-02 |
| cg16232232 |  |  | OpenSea | 4.70E-02 | 4.00E-02 | -3.61E-03 | 8.33E-04 | 7.70E-03 | -1.84E+01 |
| cg02469397 | FBXO25 | Body | OpenSea | 9.19E-01 | 8.99E-01 | -2.37E-02 | 8.34E-04 | 7.71E-03 | 1.23E+00 |
| cg21148362 |  |  | N_Shore | 1.32E-01 | 1.56E-01 | 4.17E-02 | 8.38E-04 | 7.73E-03 | -3.77E-01 |
| cg09990421 | NUBPL | TSS200 | OpenSea | 2.03E-02 | 2.20E-02 | 2.00E-03 | 8.40E-04 | 7.74E-03 | 1.60E+01 |
| cg22359892 |  |  | OpenSea | 9.78E-01 | 9.84E-01 | 2.62E-03 | 8.41E-04 | 7.74E-03 | 4.03E+00 |
| cg00732939 | HDAC8 | 1stExon;5'UTR;Body | OpenSea | 3.63E-01 | 3.98E-01 | 1.02E-02 | 8.46E-04 | 7.77E-03 | 2.55E-01 |
| cg07331673 |  |  | OpenSea | 9.04E-01 | 9.19E-01 | 4.07E-02 | 8.49E-04 | 7.78E-03 | 5.75E+00 |
| cg17668069 | UBTF | 5'UTR;TSS200 | N_Shore | 3.76E-02 | 4.41E-02 | 5.44E-03 | 8.57E-04 | 7.83E-03 | 1.61E+01 |
| cg23822289 | C1orf56;BNIPL | TSS1500;3'UTR | N_Shore | 9.53E-01 | 9.44E-01 | -2.40E-02 | 8.61E-04 | 7.85E-03 | -2.22E-02 |
| cg02296932 | MMD2 | Body | OpenSea | 8.99E-01 | 8.77E-01 | -2.90E-02 | 8.61E-04 | 7.85E-03 | -5.99E+00 |
| cg11350264 |  |  | OpenSea | 9.23E-01 | 9.54E-01 | 8.80E-02 | 8.68E-04 | 7.89E-03 | 9.25E-04 |
| cg02142005 | PACSIN2 | 5'UTR | OpenSea | 5.55E-01 | 5.03E-01 | -1.24E-01 | 8.75E-04 | 7.92E-03 | -1.05E+00 |
| cg20541527 | DLEU1 | Body | OpenSea | 8.58E-01 | 8.30E-01 | -4.91E-02 | 8.79E-04 | 7.94E-03 | 9.51E-01 |
| cg16946840 | MED12L | Body | OpenSea | 8.75E-01 | 8.53E-01 | -3.17E-02 | 8.89E-04 | 8.00E-03 | 9.37E-01 |
| cg13389611 | USP13 | TSS1500 | Island | 1.05E-01 | 1.32E-01 | 3.47E-02 | 8.92E-04 | 8.02E-03 | -7.59E-01 |
| cg03016097 | HOMER2 | TSS1500 | Island | 3.32E-02 | 3.06E-02 | -3.09E-04 | 8.96E-04 | 8.04E-03 | -1.11E+01 |
| cg11636245 | NOL10 | Body | OpenSea | 9.87E-01 | 9.89E-01 | 5.32E-05 | 9.15E-04 | 8.14E-03 | 1.18E+01 |
| cg04612952 |  |  | S_Shore | 8.50E-01 | 8.61E-01 | 2.48E-02 | 9.17E-04 | 8.15E-03 | -3.10E-01 |
| cg17307145 |  |  | OpenSea | 9.70E-01 | 9.76E-01 | 1.67E-02 | 9.19E-04 | 8.16E-03 | 5.97E+00 |
| cg12746554 | COL9A3 | Body | N_Shore | 7.40E-01 | 7.51E-01 | 9.97E-03 | 9.26E-04 | 8.20E-03 | 3.19E-02 |
| cg01424467 |  |  | N_Shelf | 8.59E-01 | 8.78E-01 | 1.96E-02 | 9.26E-04 | 8.20E-03 | -3.98E-01 |
| cg13224420 | PSD4;LOC440839 | Body | OpenSea | 9.48E-01 | 9.42E-01 | -1.40E-02 | 9.27E-04 | 8.20E-03 | -1.00E+01 |
| cg17250082 |  |  | N_Shore | 2.63E-01 | 1.88E-01 | -8.36E-02 | 9.28E-04 | 8.20E-03 | -1.84E+00 |
| cg04600055 |  |  | Island | 2.76E-01 | 3.22E-01 | 2.50E-02 | 9.32E-04 | 8.22E-03 | 1.12E+00 |
| cg15400839 | ZSWIM2 | TSS1500 | S_Shore | 2.05E-02 | 2.45E-02 | 7.04E-03 | 9.32E-04 | 8.22E-03 | -2.00E+00 |
| cg08131721 |  |  | OpenSea | 7.93E-01 | 7.34E-01 | -7.28E-02 | 9.35E-04 | 8.24E-03 | -1.40E+00 |
| cg14953665 |  |  | OpenSea | 9.26E-01 | 9.40E-01 | 2.14E-02 | 9.36E-04 | 8.24E-03 | -5.16E-01 |
| cg10318218 | FLJ26245 | Body | OpenSea | 7.37E-01 | 7.62E-01 | 2.13E-02 | 9.40E-04 | 8.26E-03 | 1.70E+00 |
| cg00224807 |  |  | OpenSea | 8.66E-01 | 9.05E-01 | 5.19E-02 | 9.41E-04 | 8.27E-03 | 1.24E-01 |
| cg15114672 | VCAN | TSS1500 | N_Shore | 8.41E-02 | 1.08E-01 | 2.93E-02 | 9.44E-04 | 8.28E-03 | -4.12E-01 |
| cg07402310 | STAT5B | 5'UTR | N_Shelf | 7.71E-01 | 7.47E-01 | -1.31E-02 | 9.44E-04 | 8.29E-03 | -7.61E-02 |
| cg07372824 |  |  | OpenSea | 1.81E-01 | 1.57E-01 | -1.27E-02 | 9.46E-04 | 8.30E-03 | -7.42E-01 |
| cg14660024 | C17orf81;DULLARD | Body;TSS1500;1stExon | Island | 5.74E-01 | 5.42E-01 | -1.05E-01 | 9.46E-04 | 8.30E-03 | -2.71E+00 |
| cg16742028 |  |  | OpenSea | 1.69E-02 | 1.96E-02 | 2.88E-03 | 9.47E-04 | 8.30E-03 | 4.93E+00 |
| cg15384356 |  |  | OpenSea | 9.24E-01 | 9.36E-01 | 1.37E-02 | 9.50E-04 | 8.31E-03 | -2.76E+00 |
| cg20134712 | DST;LOC101930010 | TSS1500;Body | OpenSea | 8.06E-01 | 7.66E-01 | -4.09E-02 | 9.52E-04 | 8.32E-03 | 1.12E-01 |
| cg05950939 | FAM132B | Body | S_Shore | 6.59E-01 | 6.70E-01 | 8.76E-03 | 9.56E-04 | 8.35E-03 | -1.21E+00 |
| cg07323088 | CRIM1 | Body | OpenSea | 9.68E-01 | 9.63E-01 | -7.99E-03 | 9.56E-04 | 8.35E-03 | 5.32E-01 |
| cg13979023 | GLRX3 | TSS1500 | N_Shore | 7.65E-01 | 7.07E-01 | -1.10E-02 | 9.56E-04 | 8.35E-03 | -6.56E-01 |
| cg26779945 | TBPL1 | TSS1500 | N_Shore | 2.10E-02 | 1.92E-02 | -3.30E-03 | 9.57E-04 | 8.35E-03 | -1.82E+01 |
| cg23431458 |  |  | OpenSea | 9.86E-01 | 9.85E-01 | -3.30E-03 | 9.65E-04 | 8.39E-03 | -2.44E+01 |
| cg05349900 | IRF2 | Body | OpenSea | 9.80E-01 | 9.82E-01 | 3.25E-04 | 9.67E-04 | 8.40E-03 | 1.59E+01 |
| cg19469447 | CYP2E1 | Body | Island | 1.33E-01 | 2.02E-01 | 3.51E-02 | 9.69E-04 | 8.42E-03 | 1.11E+00 |
| cg02251567 | AMT;NICN1 | Body;5'UTR;3'UTR;1stExon | OpenSea | 3.12E-01 | 3.48E-01 | 5.13E-02 | 9.69E-04 | 8.42E-03 | 1.85E-01 |
| cg04880418 | C11orf42 | Body | OpenSea | 9.16E-01 | 9.25E-01 | 1.19E-02 | 9.70E-04 | 8.42E-03 | -8.41E-02 |
| cg01788025 | COQ3 | TSS200 | Island | 1.16E-01 | 1.47E-01 | 3.89E-02 | 9.70E-04 | 8.42E-03 | -3.76E-01 |
| cg00318631 |  |  | S_Shore | 4.81E-01 | 5.36E-01 | 7.38E-02 | 9.73E-04 | 8.43E-03 | 3.20E+00 |
| cg22999593 | TNKS | Body | OpenSea | 9.36E-01 | 9.23E-01 | -2.36E-02 | 9.76E-04 | 8.45E-03 | 2.58E-01 |
| cg13342710 | MYLK | Body | OpenSea | 9.03E-01 | 8.84E-01 | -2.48E-02 | 9.80E-04 | 8.47E-03 | 4.78E-01 |
| cg17152444 | CLDN2 | TSS1500;5'UTR | OpenSea | 4.69E-01 | 4.88E-01 | 2.83E-02 | 9.91E-04 | 8.53E-03 | 2.11E+00 |
| cg19497942 | LINC01102 | TSS200 | OpenSea | 7.83E-01 | 7.49E-01 | -4.19E-02 | 9.92E-04 | 8.53E-03 | 1.34E-01 |
| cg07377341 | IGSF8 | TSS200 | S_Shore | 1.21E-01 | 1.35E-01 | 1.66E-03 | 9.97E-04 | 8.56E-03 | 2.04E+00 |
| cg05511138 | ARHGAP6 | 1stExon | Island | 4.06E-01 | 3.90E-01 | 5.18E-03 | 9.99E-04 | 8.57E-03 | -4.55E+00 |
| cg20151207 | CLTC | TSS200 | Island | 1.60E-02 | 1.78E-02 | 2.35E-03 | 9.99E-04 | 8.57E-03 | 9.35E+00 |
| cg14464416 | IRX5 | Body | Island | 1.32E-01 | 1.65E-01 | -7.07E-03 | 1.00E-03 | 8.58E-03 | 3.94E-01 |
| cg14073057 | RPTOR | Body | Island | 7.46E-01 | 8.07E-01 | 4.49E-02 | 1.00E-03 | 8.59E-03 | 1.74E-02 |
| cg13635299 | TRERF1 | 5'UTR | OpenSea | 9.53E-01 | 9.47E-01 | -4.53E-03 | 1.00E-03 | 8.60E-03 | -1.27E+00 |
| cg25764570 | HLA-DRA | TSS1500 | OpenSea | 6.14E-01 | 5.60E-01 | -8.15E-02 | 1.01E-03 | 8.61E-03 | -3.69E-01 |
| cg06156195 | DIP2C | Body | OpenSea | 9.80E-01 | 9.84E-01 | 2.10E-03 | 1.01E-03 | 8.64E-03 | 3.29E+00 |
| cg11969254 |  |  | OpenSea | 9.27E-01 | 9.46E-01 | 4.72E-02 | 1.02E-03 | 8.67E-03 | 4.54E-01 |
| cg01404988 | SMC1A;RIBC1 | 1stExon;TSS1500;5'UTR | Island | 1.63E-01 | 1.95E-01 | 3.91E-02 | 1.02E-03 | 8.69E-03 | 6.96E-01 |
| cg24848535 | FBXO31 | Body;TSS200 | Island | 4.43E-02 | 4.13E-02 | -1.59E-03 | 1.02E-03 | 8.70E-03 | -8.16E+00 |
| cg25284075 | CENPF | 5'UTR | Island | 1.99E-02 | 2.28E-02 | 1.79E-03 | 1.03E-03 | 8.71E-03 | 5.33E+00 |
| cg06302818 |  |  | S_Shore | 9.63E-01 | 9.66E-01 | 2.85E-03 | 1.03E-03 | 8.72E-03 | 2.13E+00 |
| cg14655242 |  |  | OpenSea | 8.02E-01 | 8.25E-01 | 3.89E-02 | 1.03E-03 | 8.72E-03 | -1.05E+00 |
| cg13733755 | PCDHGA1;PCDHGA2;PCDHGA3 | TSS1500;Body | N_Shelf | 7.07E-01 | 6.74E-01 | -2.93E-03 | 1.03E-03 | 8.73E-03 | -1.31E+00 |
| cg22798121 | GRIA1;GRIA1 | Body | OpenSea | 7.30E-01 | 7.59E-01 | 7.65E-02 | 1.04E-03 | 8.76E-03 | 3.12E-02 |
| cg04550398 | SMARCD1 | TSS200 | N_Shore | 1.62E-02 | 1.79E-02 | 1.19E-03 | 1.04E-03 | 8.77E-03 | 1.45E+00 |
| cg20687262 | SFMBT2 | 3'UTR | OpenSea | 8.81E-01 | 8.53E-01 | -3.46E-02 | 1.04E-03 | 8.77E-03 | 5.63E-01 |
| cg17300987 | FHOD3 | Body | OpenSea | 8.21E-01 | 7.90E-01 | -5.40E-02 | 1.04E-03 | 8.78E-03 | -9.90E-01 |
| cg12621279 | MED21 | TSS1500 | N_Shore | 1.97E-01 | 2.66E-01 | 1.53E-01 | 1.05E-03 | 8.83E-03 | 5.78E-01 |
| cg23616846 | LSR | Body | OpenSea | 9.79E-01 | 9.83E-01 | 2.54E-03 | 1.05E-03 | 8.83E-03 | 8.68E+00 |
| cg07826526 |  |  | OpenSea | 9.82E-01 | 9.85E-01 | 1.97E-03 | 1.05E-03 | 8.84E-03 | 1.31E+00 |
| cg23320056 | ARHGEF2 | TSS1500 | S_Shore | 4.36E-02 | 4.78E-02 | 7.44E-03 | 1.06E-03 | 8.85E-03 | 3.08E+00 |
| cg02294690 | LAP3 | Body | S_Shelf | 6.39E-01 | 5.70E-01 | -1.19E-01 | 1.06E-03 | 8.88E-03 | -2.10E-01 |
| cg07651048 |  |  | N_Shore | 8.79E-01 | 8.93E-01 | 1.99E-02 | 1.06E-03 | 8.88E-03 | -2.98E-01 |
| cg11429960 | AMOTL1 | Body | OpenSea | 9.60E-01 | 9.65E-01 | 3.74E-03 | 1.07E-03 | 8.89E-03 | 1.36E+01 |
| cg07374632 | PCSK1N | Body | Island | 4.64E-01 | 4.89E-01 | 1.59E-02 | 1.07E-03 | 8.90E-03 | 1.54E+00 |
| cg19029975 |  |  | OpenSea | 9.33E-01 | 9.59E-01 | 2.77E-02 | 1.07E-03 | 8.90E-03 | 6.80E-01 |
| cg19349999 | RNASEH2B | Body | S_Shelf | 2.67E-01 | 2.86E-01 | 3.82E-02 | 1.07E-03 | 8.90E-03 | 1.67E+00 |
| cg27401880 | MICAL2 | 3'UTR | OpenSea | 9.60E-01 | 9.71E-01 | 2.40E-02 | 1.07E-03 | 8.91E-03 | 1.00E+00 |
| cg24409539 | PAX8;LOC440839 | Body | Island | 1.65E-01 | 1.54E-01 | -3.66E-03 | 1.07E-03 | 8.94E-03 | -8.05E-01 |
| cg01205766 |  |  | OpenSea | 7.67E-02 | 7.27E-02 | -5.47E-03 | 1.08E-03 | 8.94E-03 | -3.26E+00 |
| cg02646706 | GPR139 | Body | OpenSea | 9.62E-01 | 9.53E-01 | -8.24E-03 | 1.08E-03 | 8.96E-03 | -9.14E-01 |
| cg22340571 |  |  | OpenSea | 9.56E-01 | 9.46E-01 | -6.69E-03 | 1.08E-03 | 8.97E-03 | -1.18E+00 |
| cg04964883 |  |  | OpenSea | 8.58E-01 | 8.34E-01 | -4.25E-02 | 1.09E-03 | 9.00E-03 | -2.86E+00 |
| cg22107082 |  |  | OpenSea | 9.31E-01 | 9.39E-01 | -1.71E-03 | 1.09E-03 | 9.01E-03 | 3.70E+00 |
| cg01453929 | SLC16A1 | 1stExon;TSS1500;5'UTR | Island | 1.66E-02 | 1.80E-02 | 1.62E-03 | 1.09E-03 | 9.02E-03 | 1.41E+01 |
| cg16129515 | CDK5RAP2 | ExonBnd;Body | OpenSea | 8.63E-01 | 8.28E-01 | -4.05E-02 | 1.10E-03 | 9.03E-03 | 9.94E-01 |
| cg02188048 |  |  | Island | 1.01E-01 | 1.35E-01 | 2.29E-02 | 1.10E-03 | 9.04E-03 | 5.05E-01 |
| cg18232014 |  |  | OpenSea | 9.50E-01 | 9.47E-01 | -1.17E-02 | 1.10E-03 | 9.04E-03 | -3.33E+00 |
| cg19021732 | C15orf29 | TSS1500 | S_Shore | 1.41E-02 | 1.55E-02 | 1.60E-03 | 1.10E-03 | 9.04E-03 | 3.70E+01 |
| cg19490561 |  |  | OpenSea | 8.30E-01 | 8.62E-01 | 4.84E-02 | 1.10E-03 | 9.06E-03 | 9.12E-02 |
| cg10513709 | BIK | TSS1500 | N_Shore | 3.16E-02 | 3.63E-02 | 4.93E-03 | 1.10E-03 | 9.07E-03 | 1.61E+00 |
| cg03270777 | HPDL | 1stExon | S_Shore | 8.44E-01 | 8.70E-01 | 4.86E-02 | 1.11E-03 | 9.08E-03 | -1.97E+00 |
| cg11598561 | DNAJB8 | 3'UTR | OpenSea | 9.43E-01 | 9.54E-01 | 1.37E-02 | 1.11E-03 | 9.09E-03 | -1.48E-01 |
| cg18194052 | PFN1 | TSS200 | Island | 2.23E-02 | 2.59E-02 | 5.21E-03 | 1.11E-03 | 9.09E-03 | 1.17E+00 |
| cg07832778 |  |  | OpenSea | 7.08E-01 | 7.26E-01 | 1.21E-02 | 1.11E-03 | 9.09E-03 | 1.21E+00 |
| cg17916136 | MLXIP | ExonBnd;Body | OpenSea | 9.52E-01 | 9.60E-01 | 3.95E-03 | 1.11E-03 | 9.11E-03 | -6.61E-02 |
| cg03067182 | INTS1 | Body | Island | 9.48E-01 | 9.56E-01 | 6.50E-03 | 1.12E-03 | 9.12E-03 | 8.22E-01 |
| cg06581033 |  |  | OpenSea | 9.29E-01 | 9.11E-01 | -6.38E-03 | 1.12E-03 | 9.13E-03 | -3.77E+00 |
| cg27629753 |  |  | S_Shore | 4.54E-02 | 5.28E-02 | 1.08E-02 | 1.12E-03 | 9.16E-03 | -9.42E-01 |
| cg02666129 | SPOCK1 | Body | OpenSea | 8.68E-01 | 8.55E-01 | -2.74E-02 | 1.13E-03 | 9.19E-03 | -4.62E-01 |
| cg01177167 |  |  | N_Shore | 1.58E-01 | 1.96E-01 | 2.60E-02 | 1.13E-03 | 9.19E-03 | -9.65E-01 |
| cg19793620 | IL15;IL15;IL15 | 5'UTR;Body | OpenSea | 9.81E-01 | 9.83E-01 | -1.45E-05 | 1.13E-03 | 9.21E-03 | 5.67E-01 |
| cg24971215 | AGAP3 | 5'UTR;Body | N_Shelf | 9.38E-01 | 9.17E-01 | -6.48E-02 | 1.13E-03 | 9.21E-03 | -1.77E+00 |
| cg17146950 | EFR3A | TSS1500 | N_Shore | 3.03E-02 | 2.69E-02 | -1.01E-03 | 1.14E-03 | 9.21E-03 | -2.05E+00 |
| cg01428687 | C3orf10 | TSS1500 | N_Shore | 2.14E-02 | 2.51E-02 | 2.09E-03 | 1.14E-03 | 9.23E-03 | 7.32E+00 |
| cg17064161 | RALB | Body | OpenSea | 7.10E-01 | 7.65E-01 | 1.34E-01 | 1.14E-03 | 9.24E-03 | -1.38E+00 |
| cg26444730 | BCL11B | Body | OpenSea | 7.11E-01 | 6.97E-01 | -1.44E-02 | 1.14E-03 | 9.24E-03 | -1.73E+00 |
| cg17265729 | LRRC8B | 5'UTR | Island | 1.78E-02 | 2.03E-02 | 2.83E-03 | 1.14E-03 | 9.25E-03 | 2.94E+01 |
| cg06626224 | FIZ1;ZNF524 | TSS1500 | Island | 2.82E-02 | 3.22E-02 | 3.77E-03 | 1.15E-03 | 9.26E-03 | 1.77E+00 |
| cg10724969 | CLASP2 | Body | OpenSea | 9.22E-02 | 1.14E-01 | 2.95E-02 | 1.16E-03 | 9.31E-03 | -1.15E+00 |
| cg25019718 |  |  | OpenSea | 8.88E-01 | 9.05E-01 | 4.08E-02 | 1.16E-03 | 9.31E-03 | -8.07E-01 |
| cg26120658 | TMEM161B-AS1 | Body | S_Shore | 9.69E-01 | 9.74E-01 | 1.15E-02 | 1.16E-03 | 9.32E-03 | -3.55E+00 |
| cg14752698 | CABLES1 | 5'UTR;Body | OpenSea | 9.81E-01 | 9.87E-01 | -2.14E-03 | 1.16E-03 | 9.33E-03 | 1.88E+00 |
| cg11101316 | UBE2CBP | Body | OpenSea | 8.87E-01 | 8.62E-01 | -3.06E-02 | 1.16E-03 | 9.33E-03 | 1.16E-01 |
| cg21177626 | FAM50B | TSS1500 | Island | 4.59E-01 | 5.03E-01 | 1.00E-02 | 1.16E-03 | 9.33E-03 | 1.47E+00 |
| cg03785666 | LOC100133991;FMNL1 | TSS1500;3'UTR | N_Shore | 3.58E-02 | 4.05E-02 | 3.68E-03 | 1.16E-03 | 9.33E-03 | 1.30E+01 |
| cg16207673 | ASCL4 | TSS1500 | N_Shore | 1.63E-01 | 1.80E-01 | 2.84E-02 | 1.16E-03 | 9.33E-03 | -1.60E+00 |
| cg15641500 | CARS2 | Body | Island | 9.52E-01 | 9.64E-01 | 1.47E-02 | 1.16E-03 | 9.34E-03 | 8.10E-01 |
| cg10189605 | GUSBL2 | Body | Island | 1.30E-01 | 1.56E-01 | 3.19E-02 | 1.17E-03 | 9.35E-03 | -1.13E+00 |
| cg00254448 |  |  | OpenSea | 9.89E-01 | 9.85E-01 | -5.56E-05 | 1.17E-03 | 9.36E-03 | -3.76E+00 |
| cg22006852 |  |  | OpenSea | 9.58E-01 | 9.45E-01 | -1.47E-02 | 1.17E-03 | 9.37E-03 | -2.00E-01 |
| cg21486898 |  |  | S_Shore | 9.82E-01 | 9.87E-01 | 5.31E-03 | 1.17E-03 | 9.37E-03 | 4.17E+00 |
| cg07755265 | TMOD1 | Body | OpenSea | 9.84E-01 | 9.81E-01 | -3.04E-03 | 1.17E-03 | 9.38E-03 | -2.45E+01 |
| cg07279206 | LOC400867 | Body | OpenSea | 2.56E-02 | 2.94E-02 | 3.20E-03 | 1.17E-03 | 9.39E-03 | 6.34E-01 |
| cg24450692 | SEPT4;SEPT4-AS1 | TSS1500;1stExon;Body | N_Shelf | 9.24E-01 | 9.16E-01 | -1.38E-02 | 1.17E-03 | 9.39E-03 | -9.66E-03 |
| cg04296351 | CCDC149 | TSS200 | OpenSea | 3.53E-02 | 4.10E-02 | 1.33E-02 | 1.17E-03 | 9.39E-03 | -8.79E-01 |
| cg25234088 | CEP295 | Body | OpenSea | 8.23E-01 | 7.93E-01 | -3.43E-02 | 1.18E-03 | 9.40E-03 | 4.94E-01 |
| cg24053032 | RORA | Body | OpenSea | 9.63E-01 | 9.53E-01 | -9.41E-03 | 1.18E-03 | 9.40E-03 | -6.58E+00 |
| cg17550962 | WDPCP | TSS1500;Body | OpenSea | 8.83E-01 | 8.58E-01 | -2.74E-02 | 1.18E-03 | 9.43E-03 | 3.83E-01 |
| cg11240908 | SLMO2;SLMO2-ATP5E | Body | N_Shore | 1.55E-01 | 1.29E-01 | 4.07E-03 | 1.19E-03 | 9.45E-03 | -1.17E+00 |
| cg15217684 | BIN1 | Body | OpenSea | 9.15E-01 | 9.32E-01 | 3.12E-02 | 1.19E-03 | 9.47E-03 | -2.33E+00 |
| cg26805012 | DIXDC1 | 5'UTR;1stExon;Body | Island | 4.06E-02 | 3.79E-02 | -3.26E-03 | 1.20E-03 | 9.49E-03 | -2.72E+00 |
| cg10346242 | ARF3 | TSS1500 | S_Shore | 1.31E-02 | 1.51E-02 | 2.15E-03 | 1.20E-03 | 9.52E-03 | 4.06E+01 |
| cg11102098 |  |  | N_Shelf | 8.37E-01 | 8.11E-01 | -3.09E-02 | 1.20E-03 | 9.53E-03 | 9.71E-01 |
| cg06240124 | PLK1 | TSS200 | Island | 2.66E-02 | 2.45E-02 | -2.73E-04 | 1.20E-03 | 9.53E-03 | -5.53E+00 |
| cg03492603 |  |  | N_Shelf | 9.62E-01 | 9.70E-01 | 1.03E-02 | 1.21E-03 | 9.54E-03 | 1.63E+00 |
| cg01372572 | ATP10A | Body | OpenSea | 7.20E-01 | 7.34E-01 | 4.13E-02 | 1.21E-03 | 9.55E-03 | -2.19E+00 |
| cg17268387 |  |  | OpenSea | 8.09E-01 | 8.58E-01 | 7.93E-02 | 1.21E-03 | 9.55E-03 | 9.04E-02 |
| cg13791589 | SLC45A3 | 5'UTR | Island | 7.09E-03 | 7.91E-03 | 1.32E-03 | 1.21E-03 | 9.56E-03 | 5.81E+00 |
| cg00834958 | TSC22D4 | TSS1500 | S_Shore | 2.74E-02 | 2.91E-02 | -1.69E-04 | 1.21E-03 | 9.57E-03 | 5.14E+00 |
| cg05473257 | CYP2E1 | Body | Island | 1.52E-01 | 2.20E-01 | 2.84E-02 | 1.21E-03 | 9.57E-03 | 9.26E-01 |
| cg05930098 | PLCG1 | Body | OpenSea | 8.09E-01 | 8.26E-01 | 1.88E-02 | 1.23E-03 | 9.62E-03 | -1.61E+00 |
| cg19327844 | APBB1 | TSS200;5'UTR | Island | 1.76E-02 | 1.56E-02 | -1.79E-03 | 1.23E-03 | 9.62E-03 | -1.60E+01 |
| cg17527902 | COL1A2 | Body | OpenSea | 9.62E-01 | 9.66E-01 | 9.23E-03 | 1.23E-03 | 9.63E-03 | -1.57E+00 |
| cg09861710 | PRMT10 | TSS200 | Island | 2.16E-02 | 2.30E-02 | 2.58E-03 | 1.23E-03 | 9.64E-03 | 1.02E+01 |
| cg27304653 | ADPRH | 5'UTR;Body;Body | OpenSea | 8.07E-01 | 8.28E-01 | 5.45E-02 | 1.23E-03 | 9.66E-03 | -1.56E+00 |
| cg16689193 | FARP1 | Body | OpenSea | 9.33E-01 | 9.18E-01 | -2.46E-02 | 1.24E-03 | 9.67E-03 | 5.77E-01 |
| cg02637063 |  |  | OpenSea | 9.21E-01 | 9.33E-01 | 1.35E-02 | 1.24E-03 | 9.67E-03 | 8.71E-01 |
| cg02715525 | MRPS12;SARS2 | TSS1500;Body;1stExon | S_Shore | 1.69E-02 | 1.86E-02 | 1.95E-03 | 1.24E-03 | 9.68E-03 | 1.13E+01 |
| cg17328716 | LOC650226 | TSS200 | S_Shore | 3.92E-01 | 4.42E-01 | 1.87E-02 | 1.25E-03 | 9.70E-03 | 1.22E+00 |
| cg19533489 | ZCCHC14;LOC101928737 | TSS1500 | OpenSea | 1.15E-01 | 1.61E-01 | 4.34E-03 | 1.25E-03 | 9.72E-03 | 1.05E+00 |
| cg12734446 | HEXB | 5'UTR | OpenSea | 9.53E-01 | 9.47E-01 | -1.66E-02 | 1.25E-03 | 9.73E-03 | -1.23E+00 |
| cg07491405 | CYFIP2 | Body | OpenSea | 9.40E-01 | 9.51E-01 | 2.10E-02 | 1.25E-03 | 9.73E-03 | -9.28E-01 |
| cg09478103 | ZNF485 | TSS1500 | N_Shore | 9.31E-01 | 9.44E-01 | 1.69E-02 | 1.26E-03 | 9.76E-03 | -8.62E+00 |
| cg15979670 | NOD1 | Body | OpenSea | 9.26E-01 | 9.38E-01 | 1.46E-02 | 1.26E-03 | 9.78E-03 | -1.04E+00 |
| cg19544057 | LINC00548 | TSS1500 | OpenSea | 9.77E-01 | 9.74E-01 | -5.46E-03 | 1.26E-03 | 9.78E-03 | 4.66E+00 |
| cg23594928 |  |  | N_Shelf | 9.49E-01 | 9.57E-01 | 1.25E-02 | 1.27E-03 | 9.80E-03 | -7.08E-01 |
| cg00381310 |  |  | OpenSea | 9.59E-01 | 9.69E-01 | 1.23E-02 | 1.27E-03 | 9.82E-03 | -1.06E+00 |
| cg09634707 |  |  | S_Shore | 6.48E-01 | 6.88E-01 | 5.68E-02 | 1.27E-03 | 9.82E-03 | 1.81E+00 |
| cg06854565 | PRKCZ | Body | OpenSea | 9.84E-01 | 9.86E-01 | 2.44E-03 | 1.27E-03 | 9.82E-03 | 2.96E+01 |
| cg13960067 | GMDS | Body | OpenSea | 8.56E-01 | 8.36E-01 | -3.06E-02 | 1.27E-03 | 9.83E-03 | 4.86E-01 |
| cg24182468 | VIPR2 | Body | OpenSea | 9.18E-01 | 9.33E-01 | 2.50E-02 | 1.27E-03 | 9.83E-03 | -3.16E+00 |
| cg07810861 |  |  | OpenSea | 7.60E-01 | 7.80E-01 | 1.21E-02 | 1.28E-03 | 9.85E-03 | 1.27E+00 |
| cg01957881 |  |  | OpenSea | 9.39E-01 | 9.53E-01 | 1.85E-02 | 1.28E-03 | 9.85E-03 | 5.36E-01 |
| cg23801974 | SOX2-OT | Body | OpenSea | 7.63E-02 | 9.57E-02 | 8.40E-03 | 1.28E-03 | 9.86E-03 | -1.87E+00 |
| cg15928372 | TPTEP1 | Body | S_Shelf | 8.03E-01 | 7.69E-01 | -1.43E-02 | 1.28E-03 | 9.87E-03 | 2.94E-01 |
| cg17971695 | FAM178B | Body | OpenSea | 2.82E-01 | 2.55E-01 | -3.32E-02 | 1.28E-03 | 9.87E-03 | -5.19E-01 |
| cg22223709 | PPP1R2P9 | Body | Island | 2.26E-01 | 2.78E-01 | 6.04E-02 | 1.29E-03 | 9.89E-03 | 2.13E+00 |
| cg01782904 | CORO7 | Body | OpenSea | 9.89E-01 | 9.91E-01 | 6.09E-04 | 1.29E-03 | 9.89E-03 | 3.58E+01 |
| cg07366660 | UPK3A | TSS1500 | N_Shore | 2.51E-01 | 2.26E-01 | -2.11E-02 | 1.31E-03 | 9.98E-03 | -1.61E+00 |
| cg25807671 | PNPLA6 | Body | Island | 9.84E-01 | 9.87E-01 | 1.24E-03 | 1.31E-03 | 9.98E-03 | 1.87E+01 |
| cg17517566 |  |  | OpenSea | 7.90E-01 | 8.16E-01 | 3.40E-02 | 1.31E-03 | 1.00E-02 | 6.75E-01 |
| cg12871687 | C20orf112 | TSS1500 | S_Shore | 9.09E-02 | 1.13E-01 | 7.96E-03 | 1.32E-03 | 1.00E-02 | -7.22E-01 |
| cg27016163 | HOXD13 | TSS200 | Island | 4.61E-02 | 5.67E-02 | 1.60E-02 | 1.32E-03 | 1.00E-02 | 1.02E+00 |
| cg14584103 | SLC15A4 | Body | N_Shelf | 8.30E-01 | 8.52E-01 | 4.11E-02 | 1.33E-03 | 1.01E-02 | -5.14E+00 |
| cg08824129 | CDH23 | Body | OpenSea | 8.95E-01 | 9.10E-01 | 2.04E-02 | 1.33E-03 | 1.01E-02 | -3.63E+00 |
| cg01278564 | ZFPM1 | Body | S_Shelf | 8.85E-01 | 9.03E-01 | 3.76E-02 | 1.33E-03 | 1.01E-02 | -3.68E+00 |
| cg12133778 | SSFA2 | Body | OpenSea | 9.46E-01 | 9.40E-01 | -1.96E-03 | 1.33E-03 | 1.01E-02 | -2.79E+00 |
| cg26874634 |  |  | Island | 2.53E-02 | 2.28E-02 | -3.01E-03 | 1.34E-03 | 1.01E-02 | -1.77E+01 |
| cg20825446 | LINC00499 | Body | OpenSea | 8.64E-01 | 8.40E-01 | -3.13E-02 | 1.34E-03 | 1.01E-02 | 2.26E-01 |
| cg11802925 |  |  | Island | 1.49E-01 | 1.79E-01 | 6.13E-02 | 1.35E-03 | 1.01E-02 | 8.48E-01 |
| cg08369777 | GPR161 | Body | OpenSea | 8.80E-01 | 8.97E-01 | 1.39E-02 | 1.35E-03 | 1.01E-02 | -5.93E-01 |
| cg22554952 | TYK2 | ExonBnd;5'UTR | N_Shore | 9.90E-01 | 9.92E-01 | 2.65E-03 | 1.35E-03 | 1.01E-02 | 9.34E+00 |
| cg18322589 | TACC2 | Body | OpenSea | 7.81E-01 | 8.31E-01 | 7.54E-03 | 1.35E-03 | 1.02E-02 | 5.78E-02 |
| cg08351464 | GALNT9 | Body | N_Shore | 4.41E-01 | 4.23E-01 | -2.17E-02 | 1.35E-03 | 1.02E-02 | -7.67E-01 |
| cg13350744 | NAT10; | ExonBnd;Body | OpenSea | 9.77E-01 | 9.81E-01 | 6.48E-03 | 1.35E-03 | 1.02E-02 | 6.12E-02 |
| cg15518491 | HHLA3;ANKRD13C | Body;TSS1500;1stExon | S_Shore | 4.33E-02 | 3.81E-02 | -1.59E-03 | 1.36E-03 | 1.02E-02 | -2.60E+00 |
| cg16128096 | HIVEP3 | 5'UTR;5'UTR | OpenSea | 9.71E-01 | 9.65E-01 | -1.01E-02 | 1.36E-03 | 1.02E-02 | -6.30E+00 |
| cg26957855 |  |  | OpenSea | 7.71E-01 | 7.22E-01 | -3.99E-02 | 1.36E-03 | 1.02E-02 | 1.69E-01 |
| cg22453302 | TEP1 | ExonBnd;Body | OpenSea | 9.46E-01 | 9.54E-01 | 1.94E-02 | 1.36E-03 | 1.02E-02 | -2.29E-01 |
| cg07700028 | KIAA1328 | Body | OpenSea | 8.04E-01 | 7.68E-01 | -4.67E-02 | 1.37E-03 | 1.02E-02 | 2.50E-01 |
| cg07816287 | GPC3 | TSS200 | Island | 5.32E-01 | 5.77E-01 | 3.24E-02 | 1.37E-03 | 1.02E-02 | 6.06E-02 |
